# Development of fructose-1,6-bisphosphate aldolase enzyme peptide mimics as biocatalysts in direct asymmetric aldol reactions†

**DOI:** 10.1039/d1ra06616a

**Published:** 2021-11-15

**Authors:** Thabo Peme, Dean Brady, Wanyama Juma, Maya Makatini

**Affiliations:** Molecular Sciences Institute, School of Chemistry, University of the Witwatersrand Private Bag 3, PO WITS 2050 South Africa maya.makatini@wits.ac.za +27 11 717 6708

## Abstract

This study describes the design and synthesis of mimetic peptides modelled on the catalytic active site of the fructose-1,6-bisphosphate aldolase (FBPA) enzyme. The synthesized peptides consisting of the turn motifs and catalytic site amino acids of FBPA enzyme were evaluated for catalytic activity in direct asymmetric aldol reactions of ketones and aldehydes. The influence of substrate scope, catalyst loading and solvents including water, on the reaction were also investigated. Nuclear magnetic resonance (NMR) and circular dichroism (CD) were used to determine the secondary structure of the peptides to provide an understanding of the structure–activity relationship. The peptides showed catalytic activity and the aldol products were obtained in low yields (up to 44%), but excellent enantioselectivity (up to 93%) and moderate diastereoselectivity (65 : 35).

## Abstract 2

Novel asymmetric aldol reaction catalysing fructose-1,6-bisphosphate aldolase peptide mimics with secondary structural motifs.

## Graphical abstract

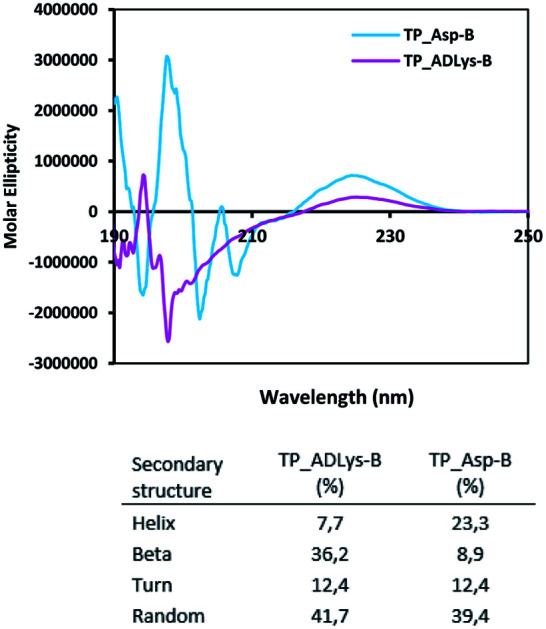

## 1. Introduction

The aldol reaction is regarded as one of the most prominent carbon–carbon bond forming organic reactions.^1–3^ In nature aldolases (Type I and Type II) are the enzymes that are used to catalyse aldol reactions. Type I aldolases follow an enamine mechanism which uses the epsilon-amino group of a lysine residue in the active site to activate the donor by forming a Schiff base as an intermediate.^4,5^ Type I aldolase, have inspired many chemists to design short peptides that mimic its natural mechanism for asymmetric catalysis. For many decades enzymes have gained considerable attention as useful catalysts for the aldol reaction, due to their fascinating properties such as their high selectivity and mild reactions conditions. Although enzymes are efficient catalysts, they still suffer from limitations such as narrow substrate tolerance, extremes of pH specificity and incompatibility with organic solvents;^5,6^ there is therefore a need for the design of catalysts that mimic enzyme activity but have fewer drawbacks.

To date, various catalysts for the aldol reactions have been reported, which include enzymes, catalytic antibodies and organocatalysts. In recent years there has been discoveries of efficient selective catalysts for a wide range of liquid- and multi-phase organic transformations.^7–9^

The majority of these advances are also in sync with attempts made by pharmaceutical and chemical industries, who have been in pursuit of rationalizing the cost of manufacturing and waste disposal in an increasing ecologically aware and economically competitive market. The area of organocatalysis, which has witnessed an immense growth over the years has received a great deal of attention owing to its green chemistry advantages such as its high efficiency, economic feasibility and ecological sustainability and its analogy to eon-perfected enzyme catalysis.^3,7^ In particular proline and its derivatives have also received wide application as organocatalysts for the asymmetric aldol reaction with the goal to improve selectivity and catalytic efficiency.^10–12^ Despite the ability of proline-related compounds to carry out a vast array of asymmetric reactions, it is still limited by poor results with certain substrates and low solubility in most organic solvents.^1^

On the other hand, the use of peptides as catalysts in organocatalytic reactions has been of interest.^4,13–15^ The structural and functional diversity of peptides provides multiple sites for modifications that can be easily attained by changing sequences of amino acids residues and acid monomers to produce optimized catalysis.^16–18^ Peptides are often regarded as simplified enzymes because of their unique structural features similar to that of enzymes, while consisting of only a fraction of the molecular mass. The catalytic function of enzyme is based on their folded states and therefore an understanding of their three dimensional folded structures presents an opportunity to design small molecules to mimic the recognition surface involved in selectivity and efficiency.^19,20^ Most of the work done on peptides as catalysts has been on simple peptide structures, usually consisting of few residues which are unlikely to adopt to a well-defined structures;^21,22^ and few studies have been conducted on well-designed peptide catalysts that incorporate secondary structures such as α-helices that may enable enzyme-like asymmetric organocatalytic reactions.

However, one major challenge in the development of these peptide catalysts is the incorporation of appropriate amino acids with suitable catalytic properties a specified peptide to mimic natural enzymes. Hence there has been some effort made in the design of peptide catalysts to mimic the fine structures of enzymes using different approaches such as rational design,^15,23^ combinatorial and library based methods.^24,25^

Akagawa *et al.*^20,26,27^ reported a series of resin supported catalytic peptides which can efficiently catalyse various asymmetric reactions, yielding products with excellent enantiomeric excess and catalytic efficiency. Their successful design incorporated a β-turn and helical motif. The turn structure consists of five terminal residues and controls the enantioselectivity, while hydrophobic helical part enhances the reactions and stabilizes the whole peptide structure under aqueous conditions. Being inspired by this approach, we became interested in applying the similar technique for the mimic of fructose-1,6-bisphosphate aldolase enzyme (EC 4.1.2.13) to afford a peptides catalyst possessing secondary structural units.

The use of water as a ‘solvent’ or co-solvent in organocatalytic reactions is of current interest due to its potential and advantages such as safety, low cost, availability, efficiency, reactivity when compared to common organic solvents.^28^ Catalytic reactions performed in aqueous systems are of interest since these systems are associated with enzymatic reactions under physiological conditions.^27^ Herein we report on the design and synthesis of peptides mimics based on the fructose-1,6-bisphosphate aldolase (FBPA) enzyme for the catalysis of direct asymmetric aldol reaction.

## 2. Results and discussion

### 2.1 Design considerations of peptide mimics

**Fig. 1:**
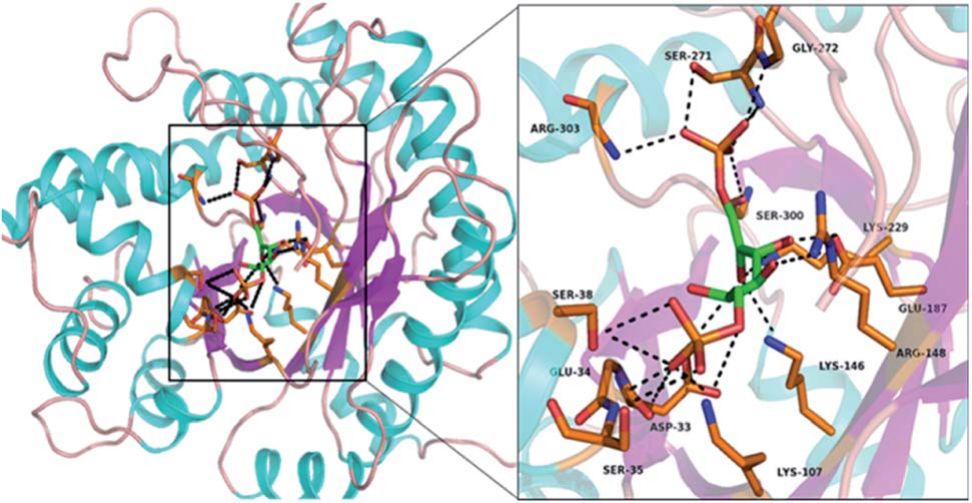
Structure depicting the FBPA enzyme's active site residues interaction with the substrate molecule (FBP), based on the sequence from: https://www.uniprot.org/uniprot/P07764; and generated using pyMOL software.

The amino acid sequence for the catalytic and turn motifs were adopted from the *Drosophila melanogaster* fructose-1,6-bisphosphate aldolase (FBPA) enzyme structure obtained from the UniProt protein database (EC 4.1.2.13; gene code: Ad1). The alignment of the selected amino acids into different peptide sequences was guided by the interactions between the active site amino acid residues with the substrate fructose-1,6-bisphosphate (FBP) (Fig. 1). In addition, the incorporated amino acids were selected based on their different functional roles in the catalytic activity of FBPA which include stabilization (Cys, Arg and Gly), steric constraints (Ala, Gly and Cys), substrate activation (Lys, Arg and Asp), proton abstraction or donation (Tyr, Asp and Glu) and covalent (nucleophilic/electrophilic) catalysis (Tyr and Asp). The turn motif consists of glycine, alanine and serine amino acids which are known to have a high turn formation propensity.^29,30^ The l-lysines were also substituted with d-lysines; introduction of unnatural amino acid residues (d-amino acid) into the peptide sequences can impose local conformational restrictions and improve selectivity. Studies have also shown that a peptide sequence consisting of both l- and d-amino form ambidextrous helical structures.^31^

We hypothesize that the peptide sequence (CRYKDGASGKDYRC) with β-turn, and α-helix motifs from the active site of the FBPA enzyme will yield a peptide with a secondary structure that can interact with most organic substrates in an aqueous media to yield aldol products.

### 2.2 Peptide synthesis

The peptides were synthesized manually and automatically using the Fmoc solid phase peptide synthesis strategy. Peptides were synthesized on Rink amide resin, and the sequential deprotection of the Fmoc protecting group from the amine to allow for the coupling of the next amino acid was performed until the full peptide was completed. Double coupling was successfully carried out for all the amino acids in the peptide sequence for 45 minutes per coupling. However, it was observed that the 6^th^ and 9^th^ amino acids (AA) were not completely coupled. Studies have shown that the coupling of glycine (a hydrophobic amino acid) into a peptide sequence as the peptide chain grows can be problematic, due to its small structural nature which induces agglomeration.^32,33^ At the end of the synthesis, the peptides were cleaved from the resin using cocktail mixtures of trifluoroacetic acid (95%), ethanedithiol (2.5%), triisopropylsilane (1.0%) and water (2.5%). The peptides were further purified by preparative RP-HPLC prior to aldol reaction catalysis screening, and were >85% pure as confirmed by LC-MS. The results of the two synthesized peptides are outlined in Table 1.

**Table 1:** Detailed summary of the two synthesized (TP_Asp and TP_ADLys) peptides

Peptide	Sequence	Calculated mass	Half mass found [M + 2H]^2+^	*R*_t_/min	Av. yield%
TP_Asp	CRYKDGASGKDYRC	C_66_H_102_N_22_O_23_S_2_, 1618.70	810.7915	*P* = 5.49	55.7
TP_ADLys	CRYKDGASGKDYRC	C_66_H_102_N_22_O_23_S_2_, 1618.70	809.8573	*P* = 6.50	62.40

Initially, the catalytic activity of the two selected peptides denoted by TP_Asp and TP_ADLys; having the sequence CYRKDGSAGDKYC, were examined for the aldol reaction of acetone and substituted *p*-nitrobenzaldehyde (Table 2). Both selected peptide sequences have the same amino acids residues except that the l-lysines in the 4^th^ and 10^th^ positions are replaced with d-lysine in the TP_ADLys peptide. The introduction of unnatural amino acid (d-amino acid) residues into the peptide sequences is also postulated to impose conformational restrictions,^31^ and to possibly improve the activity of the peptides. Taking into account the solubility of the peptide in 60% water/40% acetonitrile, the effect of water (addition) on the reaction medium and its influence on the catalyst was also investigated.

**Table 2:** Peptide-catalysed aldol reaction between acetone and substituted aromatic aldehydes^a^

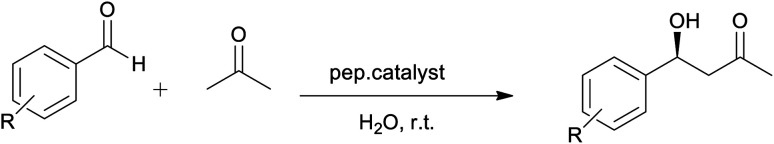						
Entry	Catalyst	R	Solvent	Time (h)	Yield^b^ (%)	ee^c^ (%)
1	TP_Asp	4-NO_2_	Acetone/H_2_O	48	16	70
2		2-NO_2_	Acetone/H_2_O	48	13	12
3		4-Cl	Acetone/H_2_O	48	14	79
4	TP_ADLys	4-NO_2_	Acetone/H_2_O	48	15	82
5		2-NO_2_	Acetone/H_2_O	48	10	7
6		4-Cl	Acetone/H_2_O	48	14	85
7^d^	TP_Asp	4-NO_2_	DMSO/H_2_O	72	Trace	—
8^d^		2-NO_2_	DMSO/H_2_O	72	Trace	—
9^d^		4-Cl	DMSO/H_2_O	72	Trace	
10^d^	TP_ADLys	4-NO_2_	DMSO/H_2_O	72	Trace	—
11^d^		2-NO_2_	DMSO/H_2_O	72	Trace	
12^d^		4-Cl	DMSO/H_2_O	72	Trace	—

a The reaction was performed using aldehyde (0.25 mmol), pep. catalyst (4 mol%), dissolved in 0.75 mL of 3 : 1 acetone/water (molar ratio 30 : 42; acetone/water), at r.t. for 48 h.

b Isolated yield.

c ee values determined by chiral-phase HPLC analysis.

d Reaction was performed using acetone (0.628 mmol, 50 μL), 0.75 mL DMSO/H_2_O (0.5173 mL DMSO, 0.237 mL water).

The aldol reaction between acetone and *p*-nitrobenzaldehyde was selected in the initial test using 4 mol% of the peptide catalyst. The results revealed poor conversions when reactions were conducted in acetone/water (entries 1 and 4, respectively), using both TP_Asp and TP_ADLys peptides. However, good enantioselectivities of up to 70 and 80% were achieved under these conditions.

Similar results are reported in literature,^18,51^ of aldol reactions proceeding slowly affording low yields and excellent ee's in acetone. Córdova *et al.* also reported low yields (25%) with good ee's of up to (65%) for the same reaction conditions.^34^ When *o*-nitrobenzaldehyde was used as substrate (entries 2 and 5), there was a notable decrease in enantioselectivity with low yields still being observed. The aldol reactions performed using *p*-chlorobenzaldehyde (entries 3 and 6) as a donor, demonstrated low reactivity but good ee's of up 85%. In another attempt to improve the reaction conditions while using the same catalysts, acetone/water was replaced with DMSO/H_2_O (entries 7–12). Unfortunately, only trace amounts of the product were detected by TLC and increasing the reaction time did not improve the yield. Although DMSO is commonly used for studies on aldol reactions involving acyclic ketones,^35,36^ studies have shown that acyclic ketones may be poor substrates in DMSO.^37,38^ This indicates inefficient formation of active enamine intermediates by acyclic ketones.^7^ There was no formation of product in the catalyst free control experiments under the same reaction conditions reported in Table 2, thus confirming the catalytic activity of the peptides.

The scope of the catalytic activity of the peptides was further investigated using cyclohexanone and substituted aromatic aldehydes (Table 3). Cyclic ketones generally have low solubility in water, and therefore their reactions are normally performed in a biphasic water/ketone system or heterogenous mixtures that comprises of a water emulsion or a suspension.^6,28^ The experimental results in Table 3 below, indicate that high ratio of water to acetone (entries 1–3), is not favourable for the reaction, as the mixture becomes immiscible and this results in the increase in the reaction time and decrease in the overall catalytic activity of the peptides.^39^ Another attempt was made with reduced amount of water and a positive outcome resulted from this modification (entries 9 and 10). The beneficial influence of reducing water, allows the dissolution of cyclohexanone consequently creating a monophasic system and a homogenous aldol reaction.^39,40^ In addition, this also proved that the partition coefficient has an influence in establishing the optimal reaction conditions. Even though this attempt gave considerably better results, the products were obtained in low yields with poor selectivities for both aldehydes. Cheng and Luo did similar work whereby the ketone was used in excess as a co-solvent and they obtained low selectivities. They further revealed that an acid additive is needed to attain good stereoselectivity.^41^ Furthermore, it was also observed that aromatic aldehydes bearing a halogen groups (entries 6–7 and 11–12) required longer reaction time or more catalyst loading as only trace amounts of the product was observed for both peptides. Similar results were also observed in literature,^7,37^ where the halogenated aldehydes yielded no product or required longer reaction time. Between the two nitrobenzaldehydes (*para*-nitrobenzaldehyde and *ortho*-nitrobenzaldehyde), it can be seen that *p*-nitrobenzaldehyde was higher than *o*-nitrobenzaldehyde. This suggests that due to steric hindrance, the nitro-group which is located at the *ortho* position might be unfavourable during the nucleophilic attack.^42^ The effect of various organic solvents was examined on peptide catalyzed aldol reaction between *p*-nitrobenzaldehyde and cyclohexanone and the results are summarized in Table 4. The use of polar aprotic solvents such as chloroform (CHCl_3_) and acetonitrile (MeCN) were not suitable for the peptide catalyzed aldol reactions (Table 4, entries 1, 2, 10 and 11) as no reaction occurred even after 96 hours. Some enzyme catalysts are sensitive towards several organic solvents, in such a way that they are more unstable in polar water miscible-solvents than in water-immiscible solvents.^43,44^ For entries 1, 2, 10, and 11 the outcome is attributed to low solubility of the peptide in CHCl_3_, causing a decrease in the reaction rate, whereas with acetonitrile the decrease could be caused by the weak hydrogen bonds that it forms with both the peptide and substrate. The reactions in polar wet protic solvent, particularly isopropanol (IPA), proceeded at a slow rate as only trace amounts of the product were observed after several days (entries 3 and 12). This outcome may suggest longer reaction times are required for the formation of the product. The aforementioned solvents were selected in this study because they are readily available in the lab and they have been reported in literature,^3,45^ to yield aldol products with high yields and good selectivity. Although high-boiling organic solvents such as DMSO and DMF are commonly used in aldol reactions studies they are often considered problematic for large-scale synthesis due to inconvenient work-up, solvent removal and recovery steps.^15^ The influence of these solvents on a small-scale reaction synthesis was then investigated. As shown in Table 4, (entries 5 and 14) when plain DMF was used there was no reaction even after 96 hours, however when a small amount of water was added (entries 7 and 16) a trace amount of the product was observed. When plain DMSO was used (entries 4 and 13), only a small amount of product was isolated. This is presumably due to high viscosity of DMSO or even DMF, it might hinder free efficient interaction between the peptide and substrates. Although the use of DMSO for proline-catalysed reactions often yields better results,^46,47^ previous studies also demonstrated no reaction or longer reaction time when anhydrous DMSO was applied.^37^ When the same reaction was performed in hydrous/wet DMSO, good yields and high selectivities (with both yield and ee) were obtained as reported in literature.^7,15,34,46^ Such findings would imply the mode of action of the peptide in this system is quite different. Moreover, it can be noted that some promising results were observed when DMSO/H_2_O was used (entries 6 and 15), with the yields of 16 and 15% and it was also intriguing to observe a considerable improvement in the ee's, to 14 and 20% respectively and moderate diastereoselectivities.

**Table 3:** Aldol reaction between cyclohexanone and various aromatic aldehydes catalysed by peptides^a^

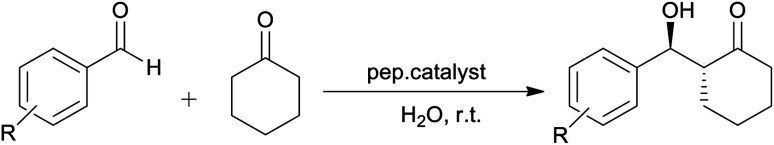						
Entry	Catalyst	R	Time (h)	Yield^b^ (%)	ee^c^ (%)	dr^d^ (*anti* : *syn*)
1^e^	TP_ADLys	4-NO_2_	48	NR	—	—
2^e^		2-NO_2_	48	NR	—	—
3^e^		4-Cl	48	NR	—	—
4	TP_Asp	4-NO_2_	48	17	7	43 : 57
5		2-NO_2_	48	11	9	47 : 53
6		4-Cl	48	Trace	—	—
7		4-Br	48	Trace	—	—
8		4-H	48	Trace	—	—
9	TP_ADLys	4-NO_2_	48	15	5	49 : 51
10		2-NO_2_	48	13	14	22 : 78
11		4-Cl	48	Trace	—	—
12		4-Br	48	Trace	—	—
13		4-H	48	Trace	—	—

a The reaction was performed using aldehyde (0.157 mmol), pep. catalyst (4 mol%), 0.45 mL of solvent, ketone/water 20 : 1 (v : v) at r.t. for 48 h.

b Isolated yield.

c ee values determined by chiral-phase HPLC analysis.

d Determine by ^1^H NMR analysis of the crude product.

e The reaction was performed in ketone/water 10 : 1 (v : v).

**Table 4:** The effect of solvent for peptide-catalysed aldol reaction using 4 mol%^a^

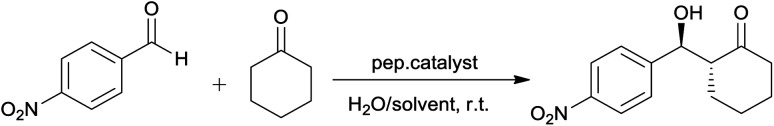						
Entry	Catalyst	Solvent	Time (h)	Yield^b^ (%)	ee^c^ (%)	dr^d^ (*anti* : *syn*)
1	TP_ADLys	MeCN/H_2_O	96	NR	—	—
2		CHCl_3_/H_2_O	96	NR	—	—
3		IPA/H_2_O	72	<10^e^	—	—
4		DMSO	72	12	5	48 : 52
5		DMF	96	NR	—	
6		DMSO/H_2_O	72	16	14	57 : 43
7		DMF/H_2_O	96	<9^e^	—	—
8		H_2_O/DMSO	72	<8^e^	—	—
9		Brine	72	13	39	31 : 69
10	TP_Asp	MeCN/H_2_O	96	NR	—	—
11		CHCl_3_/H_2_O	96	NR	—	—
12		IPA/H_2_O	72	<9^e^	—	—
13		DMSO	72	11	8	45 : 55
14		DMF	96	NR	—	—
15		DMSO/H_2_O	72	13	20	63 : 37
16		DMF/H_2_O	96	Traces	—	—
17		H_2_O/DMSO	72	<7^e^	—	—
18		Brine	72	14	12	37 : 63

a The reaction was performed using *p*-nitrobenzaldehyde (0.157 mmol, 24 mg), cyclohexanone (0.628 mmol, 70 μL), pep. catalyst (4 mol%) and dissolved in 0.75 mL (0.5173 mL solvent and 0.237 mL water) at r.t.

b Isolated yield.

c ee values determined by chiral-phase HPLC analysis.

d Determine by ^1^H NMR analysis of the crude product.

e Estimated by TLC.

These observations also illustrated that the addition of water to peptide promoted aldol reaction has some effect on influencing both the yields and stereoselectivities. The presence of small amount of water is proposed to influence the hydrogen bonding, which then alters the reaction mechanism and stereochemistry.^48^ Certain enzyme catalysts are reported to be less reactive in anhydrous solvents than in water due to constrained conformational stability.^44^ For this reason, water is essential in binding to the surface of the enzyme, to exhibit both conformational flexibility and enzymatic activity. It is also believed that water affects the hydrogen bonding of the acidic moiety and the backbone of the peptide transition state, which facilitate highly ordered transition states in aqueous conditions.^34,44^ Therefore, we postulate that the role of water in this reaction medium is of significant importance in influencing the reaction rate, and therefore improving the hydrogen bonding for the stabilization of the transition state and ground state of the substrate.^8,49^

The reactions were then carried out using higher equivalence of water in DMSO (entries 8 and 17). The reaction still proceeded slowly, causing a decrease in both the reaction rate and yields, as only trace amounts of the product was obtained. Brine is often applied as an alternative to MilliQ water when investigating the aqueous environment for reaction optimizations, and to also explore tolerance of catalyst towards the complex aqueous environment. It is reported that brine influences the reaction to proceed faster than in concentrated organic phase due to salting-out-effect.^50,51^ Gryko and co-workers also demonstrated that the use of NaCl can accelerate the organocatalysed aldol reaction in water by accelerating the aggregation of the organic material in concentrated hydrophobic pockets.^52^

To examine these hypotheses, the reactions were performed in brine solution in order to further optimize the reactions conditions to improve the yield and the influence of catalyst loading was then investigated and results are represented in Table 5 below. Compared to the results obtained in Table 4; an increase in the yields was observed when cyclohexanone with small amount of water was used (entries 1 and 5), yields of 40 and 42% for both peptide catalysts in 48 hours were obtained. The reactions further afforded much improved enantioselectivity and diastereoselectivity for the TP_Asp catalyst, whereas with TP_ADLys an ee of 6% was obtained with moderate diastereoselectivity. There were some notable results in entries 2, 6, when plain DMSO was used, although the reaction still proceeded slowly it gave moderate yields. In addition, when small amount of water was added to DMSO (entries 3 and 7), there was a significant improvement of the yields (up to 31% ee) and diastereoselectivity (65 : 45). A further increase in the yields was observed when the reaction was performed in a brine solution (entries 4 and 8), with quite reasonable ee (up to 65%) and dr (64 : 36) values were obtained. The effect of the pH was also investigated. At neutral pH both peptides (TP_Asp and TP_ADLys) have a charge of 2.9 and the calculated isoelectric point (pI_calculated_) which is the pH where the net charge of the peptide is zero found to be 9.6 when determined using a peptide calculator. It is also understood that most enzymatic catalytic reactions are dependent upon the pH of the reaction medium to function efficiently and for obtaining optimal enantioselectivity.^4,53^ Based on this, the aldol reaction in buffered aqueous media, was performed using 200 mM phosphate buffer at a pH of 8.0.^54^

**Table 5:** Effect of catalyst loading in the peptide-catalysed aldol reaction^a^

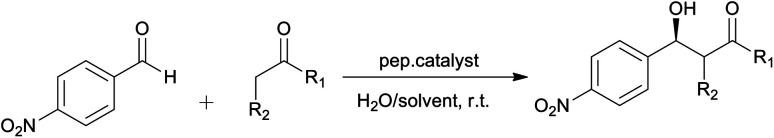							
Entry	Catalyst	R^1^, R^2^	Solvent	Time (h)	Yield^b^ (%)	ee^c^ (%)	dr^d^ (*anti* : *syn*)
1^e^	TP_Asp	(CH_2_)_4_	Ketone	48	42	20	32 : 68
2		(CH_2_)_4_	DMSO	72	21	8	54 : 44
3		(CH_2_)_4_	DMSO/H_2_O	72	25	16	65 : 35
4		(CH_2_)_4_	Brine	72	24	37	43 : 57
5^e^	TP_ADLys	(CH_2_)_4_	Ketone	48	40	6	59 : 41
6		(CH_2_)_4_	DMSO	72	20	6	58 : 42
7		(CH_2_)_4_	DMSO/H_2_O	72	27	31	64 : 36
8		(CH_2_)_4_	Brine	72	23	65	64 : 36
9^f^		(CH_2_)_4_	Buffer	72	38	80	57 : 43
10	Plain buffer	(CH_2_)_4_	Buffer	120	Trace	—	—
11^g^	TP_Asp	CH_3_, H	Ketone	48	15	93	—
12^g^	TP_ADLys	CH_3_, H	Ketone	48	13	91	—

a The reaction was performed using *p*-nitrobenzaldehyde (0.157 mmol, 24 mg), cyclohexanone (0.628 mmol, 70 μL), pep. catalyst (8 mol%) and dissolved in 0.75 mL (0.5173 mL solvent and 0.237 mL water) at r.t.

b Isolated yield.

c ee values determined by chiral-phase HPLC analysis.

d Determine by ^1^H NMR analysis of the crude product.

e The reaction was performed in ketone/water 20 : 1 (v : v).

f The reaction was performed in a phosphate buffer (pH 8.0).

g Reaction was performed in 0.75 mL of 3 : 1 acetone/water (molar ratio 30 : 42; acetone/water).

Although the buffering capacity of the phosphate buffer is reported to be maximal near 7, some reasonable results were observed, a 38% conversion, a enantiomeric excess (ee) of 80% and a diastereomeric ratio of 57 : 43. High stereoselectivity has been previously reported for biocatalysts facilitating asymmetric aldol reactions in buffer solutions (pH = 4.0–8.0).^4^ A pH of 8 was selected because previous studies have shown that for the best results the pH must be kept above the peptide's pI for better stability. In this study the use of a buffer solutions greater than a pH of 9 is restricted because the aldol reaction may become base-catalysed around this pH. In order to ascertain the influence of the buffer, a reaction with the buffer in the absence of the peptide (Table 5, entry 10) was conducted and trace amount of the product was observed after 120 hours. Thus the activity observed (entry 9) could be attributed to the structural conformation assumed by the peptide in the buffer. Lower pHs were not investigated because the catalytic lysine residue will be protonated and less nucleophilic. The low yield and longer reaction time observed for entry 9 can be accounted for by poor dissolution of the substrates, since the reaction was only carried out in an aqueous buffer. When reactions were performed using acetone as co-reactant (entries 11 and 12), the aldol products were still obtained with low yields and slight increase in enantioselectivities.

Finally, we next explored the reusability of the catalyst for the aldol reaction between cyclohexanone and *p*-nitrobenzaldehyde, as illustrated in Table 6. The catalyst (TP_ADLys) was easily recovered at the end of each reaction, through precipitation by adding diethyl ether (Et_2_O) to the combined water layers.

**Table 6:** Recovery and reuse of catalyst TP_ADLys in aldol reaction^a^

Entry	Solvent	Time (h)	Yield^b^ (%)	ee^c^ (%)	dr^d^ (*anti* : *syn*)
1	DMSO	72	Trace	—	—
2	DMSO/H_2_O	72	Trace	—	—
3	Brine	72	Trace	—	—
4^e^	Ketone	72	Trace	—	—
5^f^	Buffer	72	13	—	—
6	Buffer plain	72	13	81	81/19

a The reaction was performed using *p*-nitrobenzaldehyde (0.157 mmol, 24 mg), cyclohexanone (0.628 mmol, 70 μL), and dissolved in 0.75 mL (0.5173 mL DMSO and 0.237 mL water) at r.t.

b Isolated yield.

c ee values determined by chiral-phase HPLC analysis.

d Determine by ^1^H NMR of the crude product.

e The reaction was performed in ketone/water 20 : 1 (v : v).

f The reaction was performed in phosphate buffer (pH 8.0).

The catalyst recovery studies were performed for the TP_ADLys peptide selected aldol reactions. The results indicate that the reusability of catalyst was unsatisfactory in DMSO, brine or ketone, as only a trace amount of the product was observed after 72 hours (entries 1–2 and 3–4). This suggests the peptide cannot be efficiently reused as there is loss of catalytic activity. However, when the reaction was performed in a buffered solution, promising results were observed as yield of 13% was obtained (entry 5), and as already mentioned above, the buffer at this pH might have an effect in influencing the favourable structural conformation. Despite a longer reaction time and low yield when compared to the 38% that was obtained from the first use (Table 4, entry 9), the reaction was still achieved in high selectivities of 81% ee and dr of 81 : 19.

## 3. Peptide structural analyses and mechanism of action

**Fig. 2:**
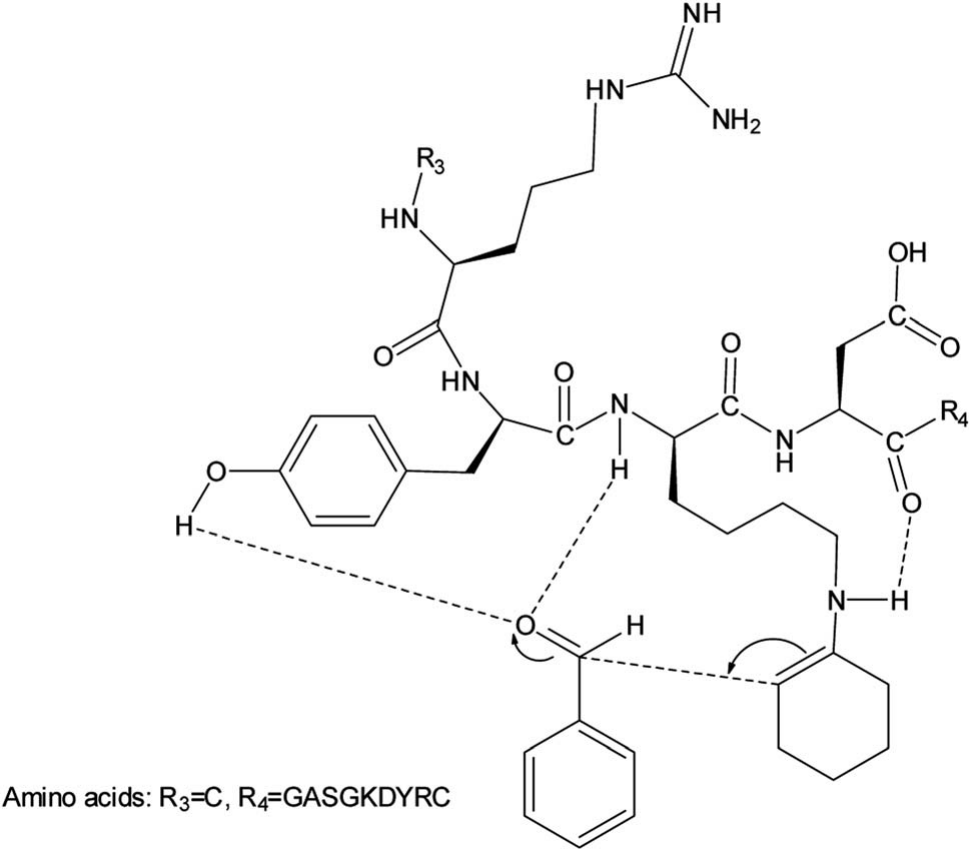
Proposed aldol reaction mechanism catalysed by peptides.

To account for the stereochemical outcome of how the new secondary peptide structures are envisaged to interact with the aldol substrates to yield aldol products, we propose a transition state similar to that suggested by the groups Jiang *et al.*^7^ and Hernández *et al.*^55^ (Fig. 2). As seen in Fig. 2, the ε-amino group of lysine residue reacts with cyclohexanone to form nucleophilic carbinolamine, followed by the dehydration to form Schiff base (imine) in the acid–base exchange reaction from aspartic acid or arginine. The arginine residue is also thought to facilitate the hydrogen bonding and cause the lysine to be in proximity with the substrates. The aldehyde is activated by the hydrogen bonding with the tyrosine's hydroxyl group along with NH hydrogen interactions of the peptide backbone.

**Fig. 3:**
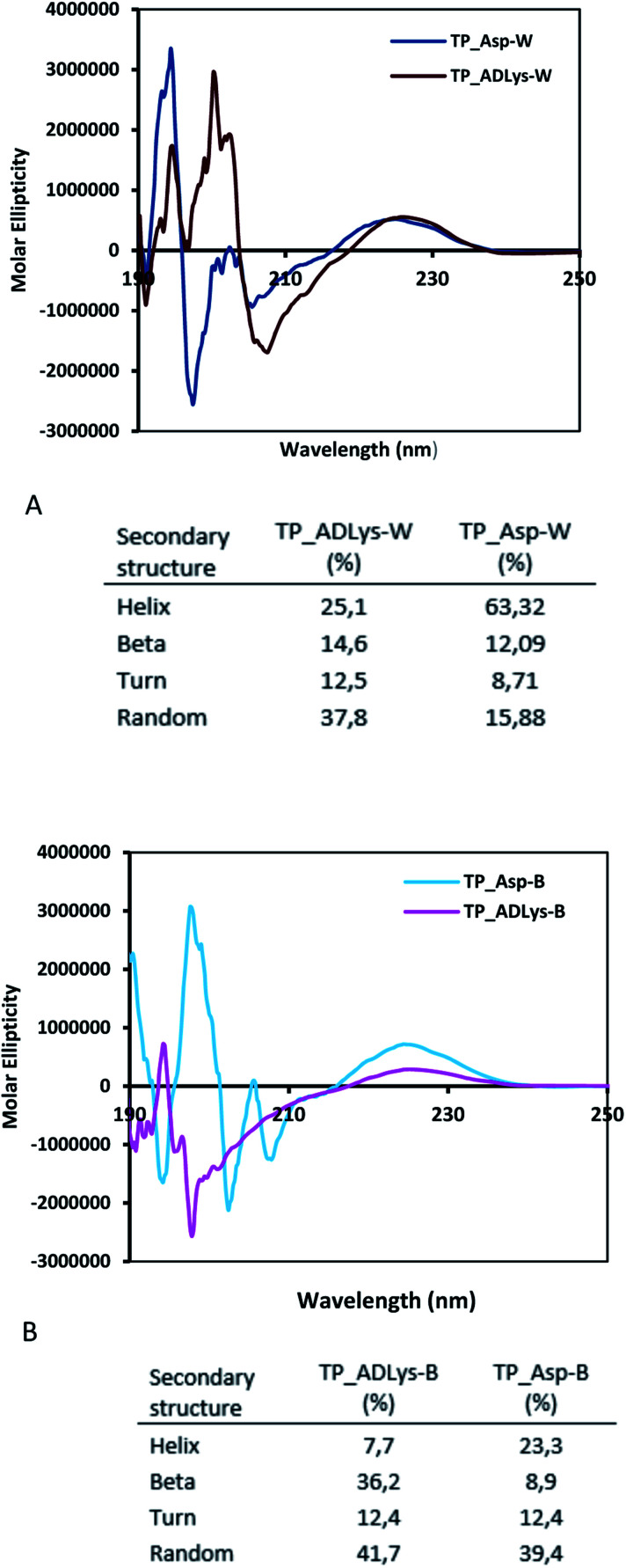
Circular dichroism studies for (A) TP_ADLys and TP_Asp peptides in water (W) and B-200 mM phosphate buffer solution (B) the percentage of secondary structure.

**Fig. 4:**
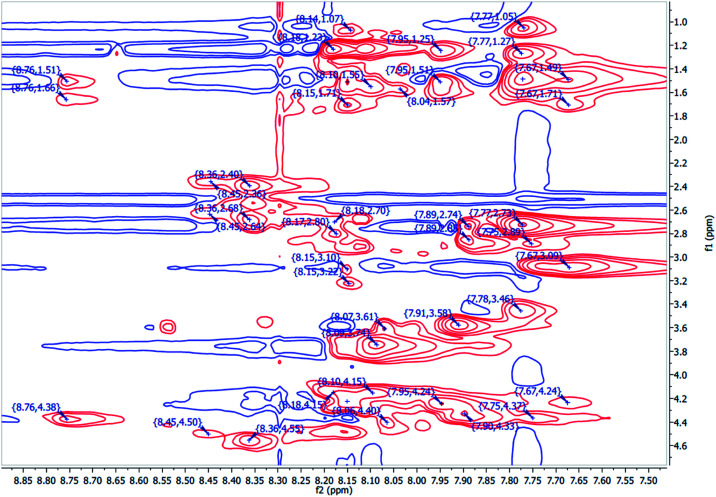
The partial ^1^HN–Hα fingerprint region of 2D the TOCSY spectrum TP_Asp of the peptide, showing individual spin system patterns.

The spectroscopic analyses were conducted using circular dichroism (CD) and nuclear magnetic resonance (NMR), to acquire information about the secondary structures of the peptide catalysts. The CD experiments were performed both in water and phosphate buffer solution. The obtained results in water for TP_ADLys, depict random coil structures going to a helix conformation (Fig. 3A). A relatively strong absorption maxima around 194 and 204 nm was also observed, suggesting β-sheets and α-helix conformation respectively with a minimum peak at 208 nm.^56^ The results for TP_Asp in water display a minimum absorption with two negative peaks around 204 and 197 nm, which is a characteristic of an α-helix structure.^57^ The CD data was further analysed using the online program to estimate peptide secondary structure formation, available at http://perry.freeshell.org/raussens.html. The results revealed that the most dominant percentage of the peptide structure for TP_ADLys was 31.8% random, followed by 25.1% helix conformation; and 14.6 and 12.5% beta and turn conformation respectively. The analysis for TP_Asp indicates that the majority of the structural elements were α-helix (63.32%), with nearly equal percentages of random (15.88%) and beta structures (12.09%). A peptide structure is proposed to adopt an extended conformation in water due to extensive solvation.^58,59^ In addition, studies suggest that helices are preferred when CD experiments are conducted in water, since hydrogen bonding promotes favourable chain–solvent interactions.^58^ In buffered solution the CD experiment for TP_ADLys shows an absorption minima around 202 and 194 nm (Fig. 3B), and a maximum band at 198 nm, which is a characteristic of random coil that deviates to a β-sheet conformation. The secondary structure estimate shows that the highest percentages of the structure were 41.7% random 36.2% beta and with almost equal amount of turn and helix structures. The result for TP_Asp spectrum differs in that it resembles a helix conformation as still being part of the dominant structure with the negative absorption maxima at 198 nm. In contrast to when the experiment was performed in water where the majority of the structure was helix (63.32%), the dominant percentage is 39.4% random, with almost equal amount of beta and turn respectively. These results demonstrate that the ability of the peptides to assume secondary structure with mixtures of conformations is influenced by the solvents. CD measurements reveals that TP_Asp possesses an alpha-helix conformation followed by random coil; whereas with TP_ADLys, adopts a beta sheet that deviates to a random coil pattern. The influence of replacing l-lysine with unnatural d-lysine was proved to alter the peptide conformation as they adopt different conformations in both solvents. To further gain complementary evidence of the secondary structure of the peptides, the 2D NMR experiments were measured. The 2D NMR experiments including TOCSY, HSCQC, COSY, and ROESY were recorded at 300 K on 500 MHz NMR spectrometer. ^1^H NMR experiments were recorded at different temperatures and mixing times for better resolution and dispersion of peaks for 2D data. The analysis was initiated by assigning the individual spin systems of amino acid residues using the fingerprint region of the amide protons of TOCSY spectrum (Fig. 4). This technique was developed by Wüthrich *et al.*,^60^ and relies on the differences in the amino acid side chains (fingerprint region) that results in different spectra patterns. Each specific residue possesses a unique pattern connecting an amide to its backbone C^α^ proton and the remaining sidechain protons. The long spin system residues such as Arg and Lys; which consists of γ and δ protons in their side chains, were unambiguously identified from TOCSY spectra. Moreover, Gly residues, which have only two α-protons whose chemical shift appears around 3.5–4 ppm, were also not difficult to identify. However, it was challenging to identify the residues corresponding to an AMX spin system (such as Tyr, Cys, Asp, Ser) based on TOCSY spectra only, since their backbone consist of one α-protons and two β-protons.

**Fig. 5:**
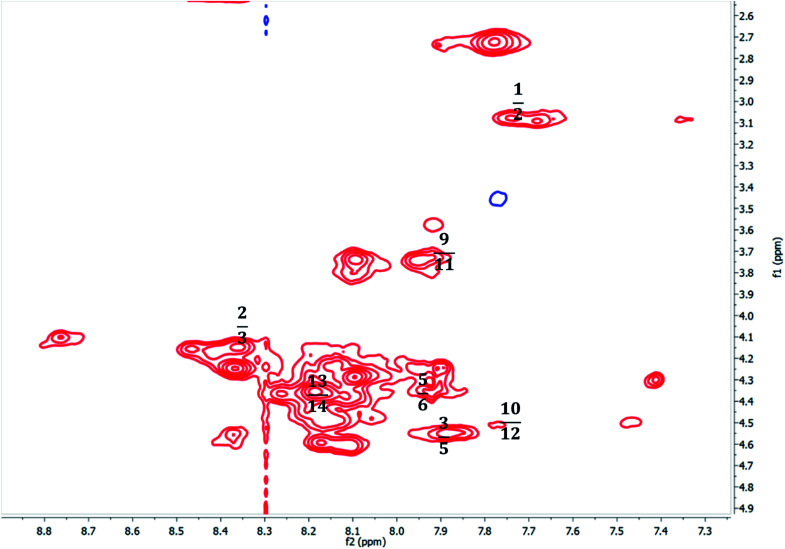
TP_Asp partial ROESY spectrum showing NH–NH and Hα–NH NOE connectivities from residues 1–14.

Furthermore, due to severe overlaps and some missing cross peaks in the fingerprint region, which is attributed to low intensity resulting from small NH–αCH coupling constant or large line width;^61^ some residues could not be unambiguously located on the TOCSY. Based on these difficulties, ROESY spectrum was then used for identifying neighbouring amino acids and to complete sequential spin specific assignment. The NOEs of the observed neighbouring amino acid residues, (for example, ^1^Hi^N^–^1^H^N^i+1 and ^1^Hi^α^–^1^H^N^i+1) in the 2D ^1^H-ROESY spectrum were utilized as an alternative to “sequential walk” technique to complete assignment (Fig. 5). All the long side chain residues (Lys and Arg) in the peptide sequence, and the two glycines were initially identified as discussed earlier and the rest on the residues were assigned based on the neighbouring NOEs connectivities in the ROESY spectrum. Since there are two glycines (6 and 9) in the peptide sequence, the NH resonance at 7.91 ppm was assumed to represent Gly6 and was used an initial starting reference point for the sequential analysis. A ROESY correlation between the peak 7.91 ppm and the alpha proton 4.33 ppm suggest a dαN(i,i+1) connectivity between these crosspeaks and the alpha proton was attributed to the aspartic acid (Asp) residue located at position 5, which is in close proximity to the Gly amide proton. On that basis, another sequential connectivity between 7.89 (Asp5–HN) resonance peak and 4.55 ppm (7.89; 4.55) was also observed, and 4.55 ppm was assigned to the alpha proton of tyrosine (Tyr3), a dαN(i,i+2) relationship. Identification of Arg2 was based on the sequential connectivity, dαN(i,i+1), between 8.36 ppm (Tyr3–HN) and 4.24 ppm, which is the alpha proton of Arg with the HN resonance peak at 7.68 ppm. There is only one alanine and serine in the peptide sequence with unique backbone spin pattern respectively and were therefore identified unambiguously from the TOCSY spectrum. A correlation, (i,i+1), between Arg2 (Hβ – 3.08) and 7.75 ppm was observed in the ROESY spectrum and the resonance, 7.75 ppm was therefore assigned as the amide proton of Cys1. Asp11 was assigned using a correlation between (Asp11HN – 7.95; Gly9Hα – 3.74) cross-peaks, a dαN(i,i−2) connectivity. All other corresponding residues were also identified based on the sequential dαN, dαβ connectivities observed throughout the sequence. The 2D ^1^H–^13^C HSQC (heteronuclear single quantum coherence) spectrum (aliphatic region) was also used to assign Cα of the corresponding spin system.

The complete sequential assignment of the amino acids residues is summarized in Table 7. The similar approach was applied for the structural elucidation of TP_ADLys peptide and the corresponding data is available in ESI.† The above results presented the qualitative picture of the primary structure of the peptide, and the secondary structure was determined using long or medium range NOE correlation. The secondary structure or backbone interactions that stabilize the peptide, can be characterized by the presence of long-range Hα–Hα or NH–Hα interactions.^62,63^ Of particular interest, a long-range correlation NOEs between the amide proton of Ala8, Ala8(HN – 8.10 ppm) and Arg13(Hβ – 1.23) was observed for TP_Asp. The presence of a medium range correlation between Ala8(HN – 8.10) and Asp11(Hβ – 1.24) NOE crosspeaks was observed for TP_ADLys These findings suggests that the structures are not linear but adopt a certain conformation. Unfortunately, due difficulties mentioned above, such as severe overlaps and missing cross-peaks, as well as finding virtually identical chemical shifts on the ROESY spectrum, other long/medium correlation could not be resolved.

**Table 7:** NMR chemical shifts (ppm) of individual spin assignment for TP_Asp

Residue	H–NH	^1^Hα	Cα	^1^Hβ	^1^Hγ	^1^Hδ	^1^Hε	ROESY
Cys1	7.75	4.36	55.08	2.89				
Argr2	7.69	4.24	48.94	3.08	1.71	1.5		(3.08; 7.75)
Tyr3	8.37	4.55	49.67	2.69, 2.40				(8.37; 4.24)
Lys4	8.15	4.23	52.86	3.11, 3.23	1.72	1.53	1.06	
Asp5	7.91	4.33	53.02	2.83, 2.89				(7.91; 4.55)
Gly6	7.92	3.58	64.63					(7.92; 4.33)
Ser7	8.06	4.41	49.87	3.61				
Ala8	8.10	4.15	52.83	1.56				
Gly9	8.09	3.74	48.58					
Lys10	7.77	4.36	43.84	3.52, 2.73	1.5	1.27	1.06	(7.77; 4.50)
Asp11	7.94	4.24	45.63	1.51, 1.24				(7.94; 3.74)
Tyr12	8.45	4.50	49.87	2.63, 2.36				
Arg13	8.18	4.48	52.85	2.8	1.23			(8.18; 4.37)
Cys14	8.75	4.37	52.84	1.69, 1.52				

## 4. Conclusions

In conclusion, the aldol addition catalytic peptides were designed and successfully synthesised using the solid phase peptide synthesis strategy. The peptides were tested for asymmetric aldol reaction catalytic activity, and they showed activity towards a selected range of substrates including aliphatic and aromatic ketone/aldehydes. The reaction between acetone and aromatic aldehydes afforded aldol products with low yields but good enantioselectivities (85–93%). Aldol products between aromatic aldehydes with cyclohexanone were obtained with low yields and selectivities. Selected organic solvents were found to provide aldol products with low yields, poor enantioselectivities and moderate diastereoselectivities. The addition of water proved to significantly improve the reaction rate and selectivities, and it was further demonstrated that a buffered water solution achieved aldol products with very high ee and moderate diastereoselectivity. Both the CD and NMR results provided summative confirmation that the peptides are not linear but possesses the evidence of secondary structural elements. The used approach for the design of the peptides might provide further advancement of the next generation of organocatalysts with improved catalytic properties. Further studies involving more detailed mechanistic studies and a broader scope of the catalyst-aqueous media systems are currently under investigation in our laboratory.

## Author contributions

MM was involved in the conceptualization, formal analysis, funding acquisition, investigation, methodology, project administration, supervision, writing-review & editing and making available resources needed for the chemistry part of the project. TP acquired and curated all the data, he wrote the original draft, performed the formal analysis, investigation, and methodology in the lab. DB was involved in funding acquisition, supervision, the writing-review and editing and provided the resources needed for the biocatalysis part of the project. WJ was involved in the determination of selectivity using liquid chromatography.

## Conflicts of interest

There are no conflicts to declare.

## Supplementary Material

RA-011-D1RA06616A-s001
