# Supplementary material for: Development of fructose-1,6-bisphosphate aldolase enzyme peptide mimics as biocatalysts in direct asymmetric aldol reactions
Source: RSC Adv. 2021 Nov 15;11(58):36670–81. doi: 10.1039/d1ra06616a (PMC9043830; doi:10.1039/d1ra06616a)
Supplement: RA-011-D1RA06616A-s001 [file RA-011-D1RA06616A-s001.pdf]

Electronic Supplementary Material (ESI) for RSC Advances.
This journal is © The Royal Society of Chemistry 2021

Electronic Supplementary Information

Development of Fructose-1,6-bisphosphate aldolase enzyme peptide mimics as biocatalysts in direct asymmetric aldol reactions

Thabo Peme,[a] Brady Dean,[a] Wanyama Juma, [a] Maya Makatini*[a]

[a]*Molecular Sciences Institute, School of Chemistry, University of the Witwatersrand, Private Bag 3, PO WITS, 2050, South Africa. Email: maya.makatini@wits.ac.za, Tel: +27 11 717 6708*

Table of contents

1. General information and materials 2

2. Synthesis of peptides catalysts 2

2.1 General procedure for the automated and manual SPPS synthesis 2

2.2 Purification of the peptides 3

3. Typical procedures for aldol reactions 3

3.1 Aldol reactions in acetone in the presence of water 3

3.2 Procedures for the aldol reactions between aromatic aldehydes and cyclohexanone ...4

3.3 General procedure for aldol reaction between cyclohexanone and aromatic aldehydes in organic solvent 4

3.4 Catalyst recyclability for peptide catalyzed aldol reaction 5

4. Analytical data for Aldol products 5

5. LC-MS spectra for TP_Asp and T_ADLys peptide catalysts 7

6. NMR spectra for peptide structures 15

7. Selected NMR spectra for aldol products 27

8. Selected chiral HPLC chromatograms for aldol products 32

8.1 Table A: Analytical data and Chiral-phase HPLC spectra analysis for selected aldol products using Lux 5μm Cellulose-1 chiral column 52

9. Selected NMR spectra for determination of the syn/anti (dr) for aldol products by ^{1}H NMR 54

1. General information and materials

All solvents and reagents were obtained from commercial sources. Solvents for column chromatography (ethyl acetate and hexanes) were purchased from Protea Chemicals (South Africa) and distilled before use to remove non-volatile components. Aldol reactions were monitored using thin layer chromatography (TLC) plates (0.2 mm silica gel 60 with fluorescent indicator UV254) were obtained from Sigma-Aldrich and further visualization was done by staining with potassium permanganate (KMnO4) solution followed by heating. Purification of aldol products was conducted on Merk normal silica gel (particle size 0.063-0.200 mm) and flash silica gel (particle size 0.040-0.063).

^{1}H NMR and ^{13}C NMR spectra were recorded on either a Bruker AVANCE 300 MHz, Bruker AVANCE 400 MHz or on a Bruker AVANCE III 500 MHz spectrometer. 2D NMR spectra which include COSY, 13C-HSQC, TOCSY, NOESY and ROESY experiments, were recorded at 293, 300, and 308 K on a 500 MHz NMR Bruker III 500 MHz spectrometer. All 2D spectra were recorded at the phase sensitive mode using time proportional phase increment (TPPI). The residual water peak of DMSO-d6 was suppressed by a presaturation pulse of 2 s duration. The first NOESY experiments were recorded with mixing time of 150, 200 and 250 ms; ROESY spectrum were recorded with mixing times of 100, 150, 200, 250 and 300 ms; while TOCSY was recorded with mixing times of 48, 64, 70, 80 and 100 ms; each increment was the sum of 32 scans with a relaxation delay of 2.0 s; 2048 data point were collected per experiment.

The enantiomeric excess (ee) was determined by chiral high performance liquid chromatography (HPLC) analysis on a Dionex HPLC Ultimate 3000 instrument (CHROMELEON version 6.80 software); coupled to a pump and photodiode array detector. A Lux 5µ cellulose-2 column was used for the analysis with hexane and isopropyl alcohol (IPA) as the mobile phase**.** CD spectra were recorded on a JASCO J-18 spectropolarimeter between the range of 190 to 250 nm in the specified solvents (water and phosphate buffer), with 10 scans at 20°C. Analytical LC-MS analysis was carried out on an Ultra High Performance Liquid Chromatography (Thermo Scientific Ultimate 3000, RS diode array detectors)-High Resolution Mass Spectrometer (Bruker Compact quadruple time of-flight) coupled to Diode Array (215 and 254 nm).

2. Synthesis of peptides catalysts.

Peptide were synthesized on an automated Protein Technologies, Inc PS-3TM peptide synthesizer and manually following the general Fmoc solid phase synthesis.

2.1 General procedure for the automated and manual SPPS synthesis

2.1.1-Swelling, activation and coupling of the first amino acid

The Fmoc-rink amide resin, 600 mg, (0, 160 mmol/g) was swelled in DMF (10.0 mL) for 20 minutes in a 70 mL glass reaction vessel with fritted filters. The DMF was removed by suction and a 20% piperidine solution in DMF (5 mL) was added and mixed by bubbling for 5 minutes with inert nitrogen gas (N2). The piperidine solution was then filtered out and the

resin was washed with DMF (5×5 mL) for 30 sec second per each wash. The first amino acid cysteine (1.20 mmol, 0.2 M), and coupling reagent HBTU (1.14 mmol, 0.19 M) were dissolved in a solution of 1.0 M DIPEA in DMF (6 mL) and added into the reaction vessel. An additional 6 mL of DMF was also added. The reaction mixture was allowed to react for 45 minutes while a gentle flow of N_2 was bubbled into the reaction. For double coupling, the resin was washed with (3×5 mL) DMF, and the coupling was repeated using the same mixture for 60 minutes. The resin was then washed with (3×5 mL) DMF, and (2×5mL) DCM.

After the first coupling, Fmoc protecting group was removed using (2×10 mL) 20% piperidine solution and the resin was washed with DMF (5×5 mL) before the second amino acid was coupled. A solution containing 6 mL of 1.0 M DIPEA, (1.14 mmol, 0.19 M) HBTU, and (1.02 mmol, 0.2 M) arginine was added to the resin. The reaction mixture was mixed gently by bubbling nitrogen gas for 45 minutes. The remaining amino acids in the sequence were also coupled using the same procedure until the full peptide was synthesized.

2.1.2 General procedure for cleaving peptides from the resin

The resin bound peptide was washed with (3 ×5 mL) DMF and then (3×5 mL) DCM and dried by suction. A cleavage cocktail (10 mL) containing 94% TFA: 2.5%EDT:2.5%H_2O:1%TIS was then reacted with the resin bound peptide for 3 hrs. The cleavage solution was filtered off, and the resin was washed with 5 mL of TFA and the filtrated was divided into portions in and poured into 50 ml centrifuged tubes. Cold diethyl ether was added to the filtered solution upon which a white precipitate of the crude peptide was formed. The two samples were centrifuged at 5000 rpm for 10 minutes. The step was repeated 3 times with cold diethyl ether. The obtained white precipitate was then dissolved in 10 mL (60/40; H_2O/MeCN) for further analysis and purification.

2.2 Purification of the peptides

Peptides were purified via an Agilent 1260 Infinity semi-preparative HPLC system with a UV/VIS detector and an automated fraction collector on a Kinetix® 5 µm B-C18, 100 Å (250 ×230 nm) column. A two-buffer system was employed; Buffer A consisted of 0.1% formic acid in H_2O and buffer B consisted of 0.1% formic acid in CH_3CN. A flow rate of 20 ml/min or 15 ml/min and UV wavelengths of 215 and 254 nm were utilized

3. Typical procedures for Aldol reaction

3.1 Aldol reactions in acetone in the presence of water

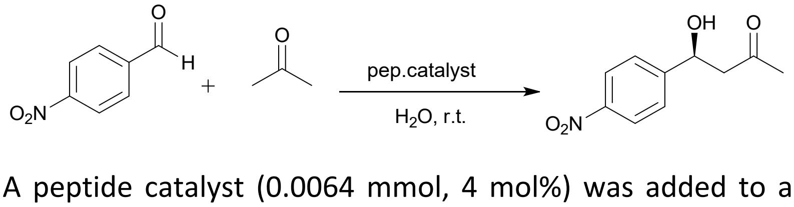

A peptide catalyst (0.0064 mmol, 4 mol%) was added to a vial containing 0.75 mL of 3:1 acetone/water and the mixture was stirred for 15 min. An acceptor aldehyde (0.157 mmol, 24 mg) was then added, and the resulting reaction mixture was stirred vigorously at room temperature for 24-72 hours. The reaction was stopped upon completion as indicated by

TLC and acetone was evaporated under reduced pressure. The crude product was extracted using (3 ×10 mL) ethyl acetate (EtOAc) and 2 mL water. The combined organic phases were dried over Na_2SO_4 and concentrated under reduced pressure. The crude product was then purified by flash silica gel column chromatography using ethyl acetate/hexane (1:3). The pure product was then subjected to chiral-phase HPLC analysis to determine the ee.

3.2 Procedures for the aldol reactions between aromatic aldehydes and cyclohexanone

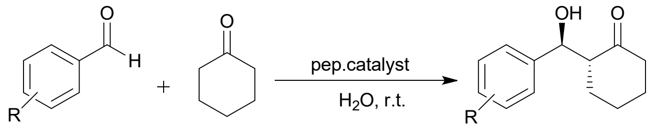

3.2.1 Aldol reaction between cyclohexanone and aromatic aldehydes (homogenous)

A peptide catalyst (0.0064 mmol, 4 mol%) was added to the ketone (0.45 mL) in water (40µL) and stirred for 15 minutes. An acceptor aldehyde was then added, and the resulting reaction mixture was left to stir for 24-72 hours at room temperature. The reaction was stopped upon completion as indicated by TLC. The reaction was quenched by extraction with (3×10 mL) EtOAc and brine solution (2 mL). The combined organic extract was washed with brine and dried with Na_2SO_4, filtered and concentrated in vacuo. Diastereomeric ratio of the crude product was determined by 1H NMR analysis. The crude aldol product was purified by flash silica-gel column chromatography using EtOAc/Hexane (1:3) and the desired aldol product was subjected to chiral-phase HPLC analysis to determine the ee.

3.2.2 Aldol reaction between cyclohexanone and aromatic aldehydes (heterogenous)

A peptide catalyst (0.0064 mmol, 4 mol%) was added to a solution of a ketone (0.450 mL) and 60 µL of water and stirred for 15 minutes. An acceptor aldehyde (0.157 mmol, 24 mg) was then added to reaction mixture which was stirred for 24-72 hours. The reaction was monitored by TLC and the reaction was quenched by extraction with (3×10 mL) EtOAc and (1×2 mL) brine solution. The combined organic layers were dried with Na2SO4, filtered and concentrated in vacuo. The crude product was purified by flash chromatography on silica gel using EtOAc: hexane (1:3).

3.3 General procedure for aldol reaction between cyclohexanone and aromatic aldehydes in organic solvent

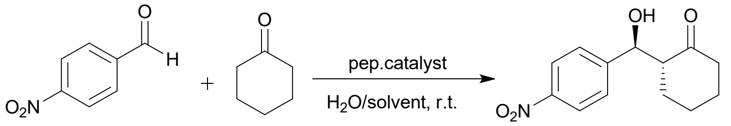

A peptide catalyst (0.0064 mmol, 4 mol%) was added to a vial containing a 0.75 mL solvent mixture (0.5173 mL solvent and 0.237 mL water) and cyclohexanone (0.628 mmol, 70 µL) and the mixture was stirred for 15 minutes. An acceptor aldehyde (0.157 mmol, 24 mg) was then added to the reaction mixture which was stirred for 24 -72 hours. The reaction progress was monitored by TLC and was extracted with EtOAc (3×10 mL) and brine solution

(1×2.0 mL). The combined organic layers were dried over Na_2SO_4 and concentrated in vacuo. Diastereomeric ratio of the crude product was determined by ^1H NMR analysis. The crude product was purified by flash chromatography on silica-gel with hexane/ethyl acetate (3:1). The pure products were then analyzed by the chiral HPLC and the ee values were determined

3.4 Catalyst recyclability for peptide catalyzed aldol reaction

The filtered aqueous layers obtained after extraction of the crude product, were combined and washed with Et_2O (3 mL) and C_2H_5O (4 mL). The peptide was catalyst obtained was after drying and decantation of the resuspended mixture. The recycled peptide was added to a vial containing a 0.75 mL solvent mixture (0.5173 mL solvent and 0.237 mL water) and

4. Analytical data for Aldol products

4-Hydroxy-4-(4'-nitrophenyl)-butan-2-one (TP_A1N4ACE)

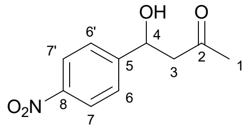

Yellow solid: R_f = 0.27 (30% ethyl acetate/hexane). IR (Vmax/cm-1): 3459 (O-H), 3118 (=C-H), 3068 (C-H), 1574 (C=C), 1706(C=O), 1323(C-O). ^1H NMR (500 MHz, CDCl3) δ: 8.22 (d, 2H, H-7& H-7'), 7.54 (d, 2H, H-6 and H-6'), 5.30 – 5.21 (m, 1H, H-4), 3.55 (brs, J = 3.3 Hz, 1H, OH), 2.87 – 2.77 (m, 2H, H-3), 2.23 (s, 3H, H-1).^{13}C NMR (126 MHz, CDCl3) δ: 208.44(C-2), 126.45(C-6), 126.34(C-6'), 123.80(C-7) , 123.72(C-7') , 68.62(C-4) , 50.83(C-3) , 30.77(C-1).HRMS (ESI) m/z: Calculated for $C_{10}H_{11}NO_4$:209.068, found: 232.0910[M+23]$^+$

4-Hydroxy-4-(2'-nitrophenyl)-butan-2-one (TP_A2N2ACE)

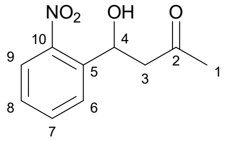

Colourless solid: R_f = 0.32 (30% ethyl acetate/hexane). IR (Vmax/cm-1): 3461 (O-H), 3068(=C-H), 2922 (C-H), 1609 (C=O), 1518 (C=C), 1333(C-O). ^1H NMR (500 MHz, CDCl3) δ 8.04 – 7.80 (m, 2H, H-9 and H-8), 7.77 – 7.58 (m, 1H, H-6), 7.50 – 7.38 (m, 1H, H-7), 5.68 (dd, 1H, H-4), 3.79 (brs, 1H, OH), 3.11 (d, 1H, H-3), 2.74 (dd, J = 17.8, 9.4 Hz, 1H, H-3), 2.24 (s, 2H H-1).^{13}C NMR (126 MHz, CDCl3) δ: 208.76 (C-2), 147.12(C-10), 138.47(C-5), 133.82(C-7), 128.28(C-6), 128.19(C-9), 124.43(C-8), 65.60(C-4), 51.12(C-3), 30.43(C-1). HRMS (ESI) m/z: Calculated for $C_{10}H_{11}NO_4$:209.07, found: 232.0583 [M+23]$^+$

4-Hydroxy-4-(4'-chloropheny)-butan-2-one (TP_A3ClACE)

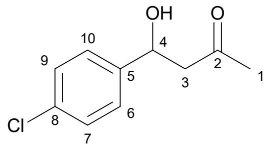

Colourless oil: R_f = 0.28 (30% ethyl acetate/hexane). IR (Vmax/cm-1): 3421 (O-H), 3117(=C-H), 2967 (C-H), 1701 (C=O), 1519 (C=C), 1315(C-O). 1H NMR (500 MHz, CDCl3) δ: 7.33 – 7.26 (m, 4H, Ar-H), 5.12 (d, J = 9.0, 3.4 Hz, 1H, H-4), 3.47 (brs, 1H, OH), 2.89 – 2.72 (m, 2H, H-3), 2.19 (s, 3H, H-1). 13C NMR (126 MHz,) δ: 208.86, 141.25, 133.30, 128.65, 127.03, 69.16, 51.82, 30.76. HRMS (ESI) m/z: Calculated for $C_{10}H_{11}ClO_4$:198.0488, found: 221.0334 $[M+23]^+$

2-(Hydroxy(4-nitrophenyl)methyl)cyclohexanone (TP_A4N4CY)

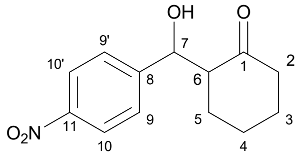

Yellowish solid: Rf = 0.26 (30% ethyl acetate/hexane). IR (Vmax/cm-1): 3426(O-H), 3077(=C-H), 2949 (C-H), 1698 (C=O), 1524 (C=C), 1024(C-O). ^{1}H NMR (500 MHz, CDCl3) δ: 8.21 (d, J = 8.9, 2.3 Hz, 2H,H-10 and H-10'), 7.50 (d, J = 7.5 Hz, 2H, H-9 and H-9'), 4.90 (d, J = 8.3, 3.0 Hz, 1H, H-7), 4.07 (brs, 1H, OH), 2.63 – 2.55 (m, 1H, H-6), 2.54 – 2.45 (m, 1H, H-2a), 2.41 – 2.31 (m, 1H, H-2b), 2.16 – 2.08 (m, 1H-H-3a), 1.72 – 1.64 (m, 1H-H-3b), 1.63 – 1.48 (m, 4H, H-4 and H-3). ^{13}C NMR (126 MHz, CDCl3) δ: 214.73 (C-1), 147.96 (C-11), 127.87 (C-8), 126.59 (C-9 and C-9'), 123.52 (C-10 and C-10'), 74.05 (C-7), 70.14 (C-6), 57.20 (C-2), 42.69 (C-5), 30.77 (27.64 , 24.75 HRMS (ESI) m/z: Calculated for $C_{13}H_{15}NO_4$:249.1001, found 272.0890 $[M+23]^+$.

2-Hydroxy(2-nitrophenyl)methyl)cyclohexanone (TP_A5N2CY)

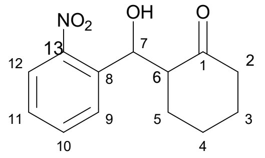

Yellow oil: Rf = 0.29 (30% ethyl acetate/hexane). IR (Vmax/cm-1): 3454(O-H), 2922 (=C-H), 2853 (C-H), 1693 (C=O), 1512 (C=C), 1199(C-O).1H NMR (500 MHz, CDCl3) δ: 7.94 – 7.72 (m, 2H), 7.64 (t, J = 7.7 Hz, 1H), 7.43 (t, J = 7.8 Hz, 1H), 5.45 (d, J = 7.0 Hz, 1H), 4.07 (d, J = 37.1 Hz, 0H), 2.82 – 2.68 (m, 1H), 2.51 – 2.28 (m, 2H), 1.92 – 1.52 (m, 5H), 1.26 (d, 2H). 13C NMR (126 MHz, CDCl3) δ 214.96 (C-1), 148.73 (C-13), 136.63 (C-8), 133.07 (C-10), 129.00 (C-9), 128.40 (C-12), 124.10 (C-11), 69.81 (C-7), 57.31 (C-6), 42.85 (C-2), 31.14 (C-5), 27.77 (C-3), 25.00 (C-4). Calculated for C13H15NO4 :249.1001, found 272.0890 [M+23]+.

5. LC-MS spectra for TP_Asp and T_ADLys peptide catalysts

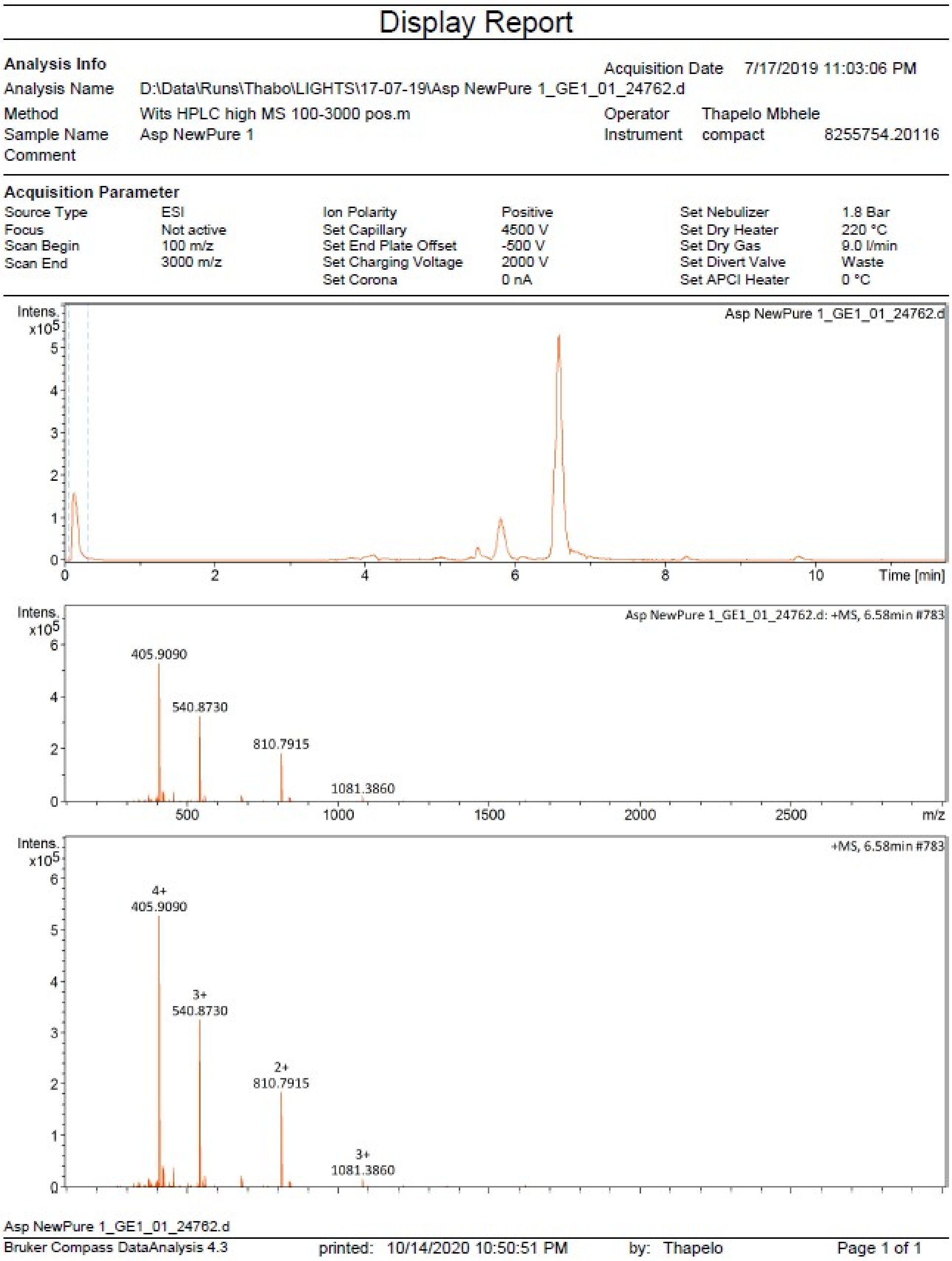

Figure S1: LC-MS spectrum of TP_Asp conformation 1 (peak 1)

Display Report

Analysis Info

Analysis Name	D:\Data\Runs\Thabo\LIGHTS\17-07-19\Asp NewPure 2_GE2_01_24764.d	Acquisition Date	7/17/2019 11:30:38 PM
Method	Wits HPLC high MS 100-3000 pos.m	Operator	Thapelo Mbhele
Sample Name	Asp NewPure 2	Instrument	compact 8255754.20116
Comment			

Acquisition Parameter

Source Type	ESI	Ion Polarity	Positive	Set Nebulizer	1.8 Bar
Focus	Not active	Set Capillary	4500 V	Set Dry Heater	220 °C
Scan Begin	100 m/z	Set End Plate Offset	-500 V	Set Dry Gas	9.0 l/min
Scan End	3000 m/z	Set Charging Voltage	2000 V	Set Divert Valve	Waste
		Set Corona	0 nA	Set APCI Heater	0 °C

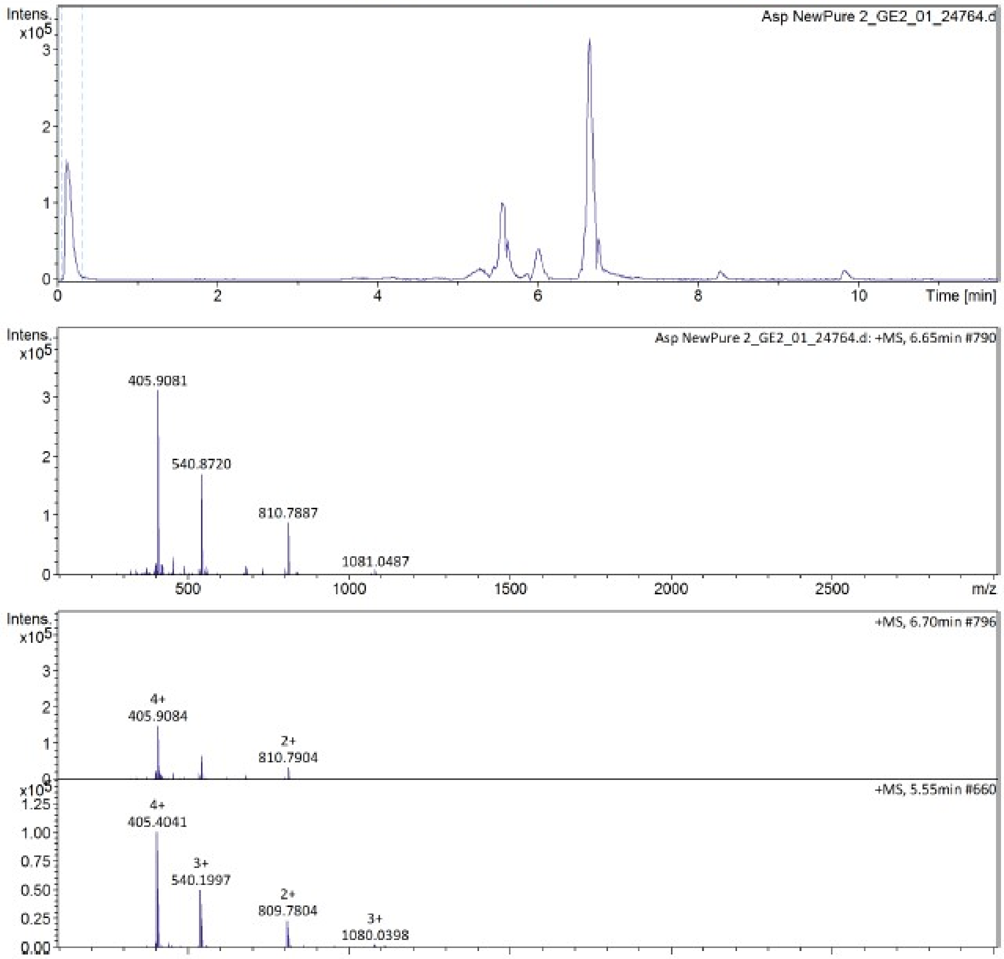

Asp NewPure 2_GE2_01_24764.d

Bruker Compass DataAnalysis 4.3 printed: 10/14/2020 10:55:24 PM by: Thapelo Page 1 of 1

Figure S2: LC-MS spectrum of TP_Asp conformation 2 (peak 2)

Display Report

Analysis Info

Analysis Name	D:\Data\Runs\Thabo\LIGHTS\17-07-19\Asp Pure 3 real_GE3_01_24782.d	Acquisition Date	7/18/2019 12:20:20 PM
Method	Wits HPLC high MS 100-3000 pos.m	Operator	Thapelo Mbhele
Sample Name	Asp Pure 3 real	Instrument	compact 8255754.20116
Comment			

Acquisition Parameter

Source Type	ESI	Ion Polarity	Positive	Set Nebulizer	1.8 Bar
Focus	Not active	Set Capillary	4500 V	Set Dry Heater	220 °C
Scan Begin	100 m/z	Set End Plate Offset	-500 V	Set Dry Gas	9.0 l/min
Scan End	3000 m/z	Set Charging Voltage	2000 V	Set Divert Valve	Waste
		Set Corona	0 nA	Set APCI Heater	0 °C

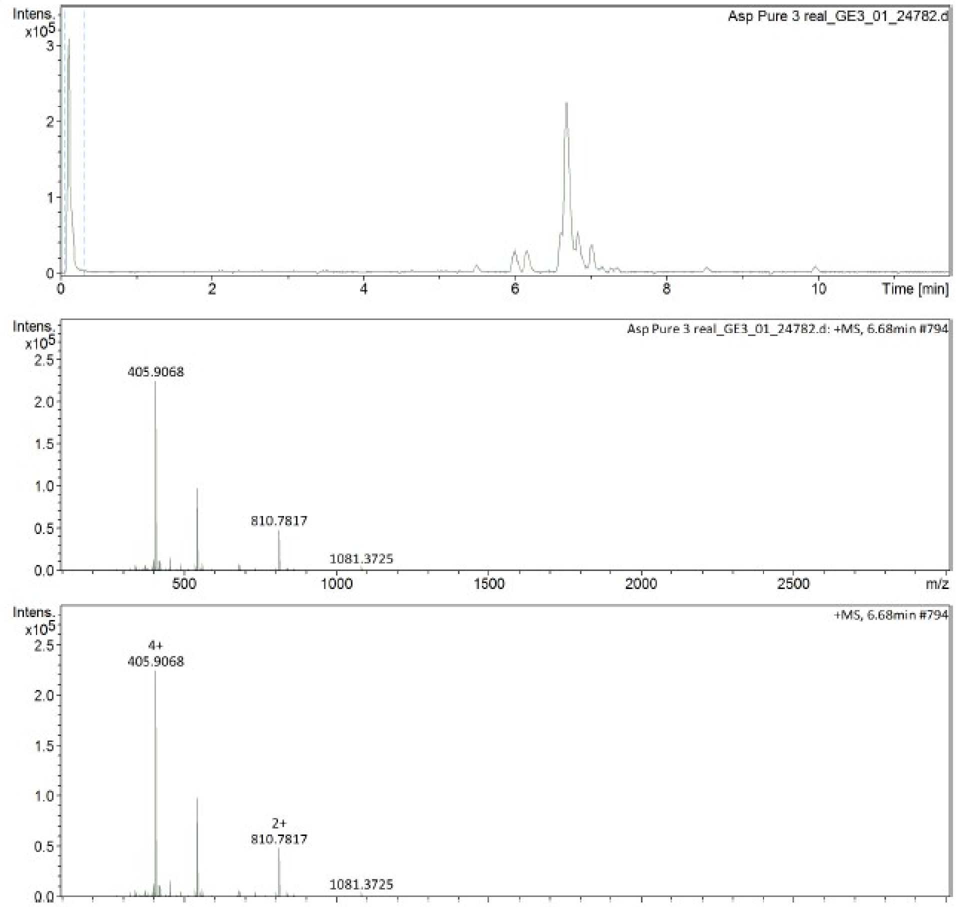

Asp Pure 3 real_GE3_01_24782.d

Bruker Compass DataAnalysis 4.3 printed: 10/14/2020 10:58:32 PM by: Thapelo Page 1 of 1

Figure S3: LC-MS spectrum of TP_Asp conformation 3 (peak 3)

Display Report

Analysis Info

Analysis Name	D:\Data\Runs\Thabo\LIGHTS\17-07-19\Asp NewPure 4_GE4_01_24769.d	Acquisition Date	7/18/2019 12:37:19 AM
Method	Wits HPLC high MS 100-3000 pos.m	Operator	Thapelo Mbhele
Sample Name	Asp NewPure 4	Instrument	compact 8255754.20116
Comment			

Acquisition Parameter

Source Type	ESI	Ion Polarity	Positive	Set Nebulizer	1.8 Bar
Focus	Not active	Set Capillary	4500 V	Set Dry Heater	220 °C
Scan Begin	100 m/z	Set End Plate Offset	-500 V	Set Dry Gas	9.0 l/min
Scan End	3000 m/z	Set Charging Voltage	2000 V	Set Divert Valve	Waste
		Set Corona	0 nA	Set APCI Heater	0 °C

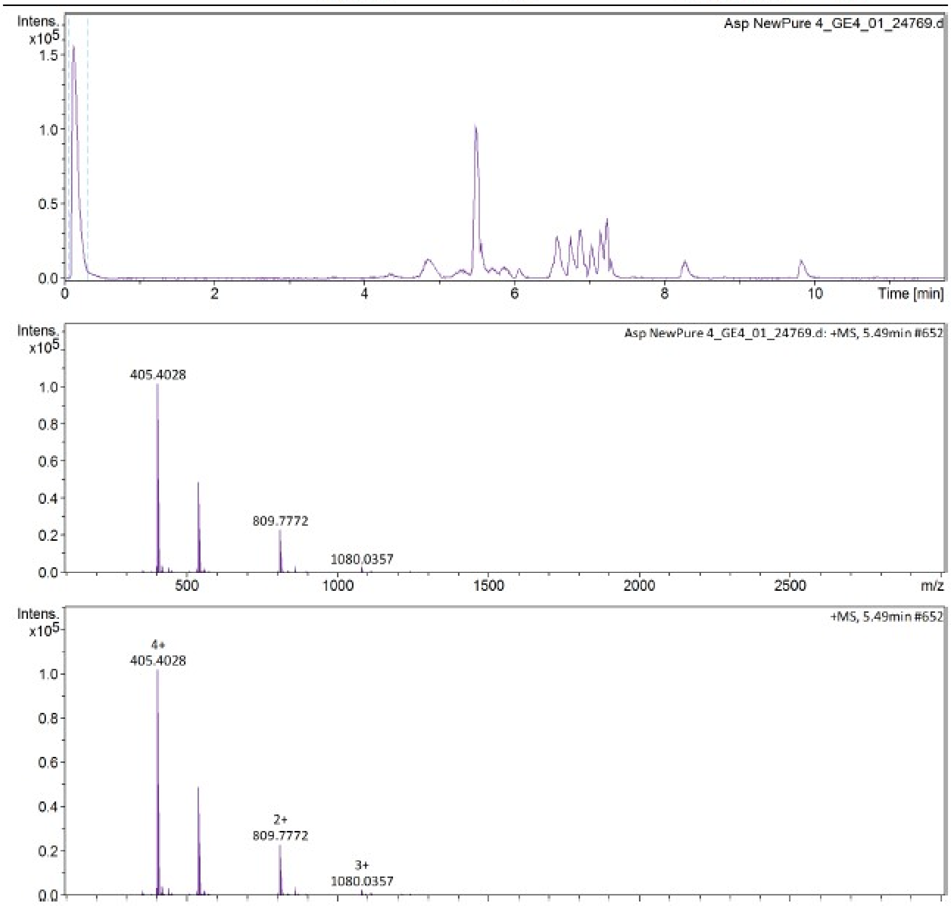

Asp NewPure 4_GE4_01_24769.d

Bruker Compass DataAnalysis 4.3 printed: 10/14/2020 11:06:28 PM by: Thapelo Page 1 of 1

Figure S4: LC-MS spectrum of TP_Asp conformation 4 (peak 4)

Display Report

Analysis Info

Analysis Name	D:\Data\runs\thabo\lights\07-10-19\D Lys final Pure 1 re_GB3_01_27540.d	Acquisition Date	10/7/2019 1:10:33 PM
Method	Wits HPLC high MS 100-3000 pos.m	Operator	Thapelo Mbhele
Sample Name	D Lys final Pure 1 re	Instrument	compact 8255754.20116
Comment			

Acquisition Parameter

Source Type	ESI	Ion Polarity	Positive	Set Nebulizer	1.8 Bar
Focus	Not active	Set Capillary	4500 V	Set Dry Heater	220 °C
Scan Begin	100 m/z	Set End Plate Offset	-500 V	Set Dry Gas	9.0 l/min
Scan End	3000 m/z	Set Charging Voltage	2000 V	Set Divert Valve	Waste
		Set Corona	0 nA	Set APCI Heater	0 °C

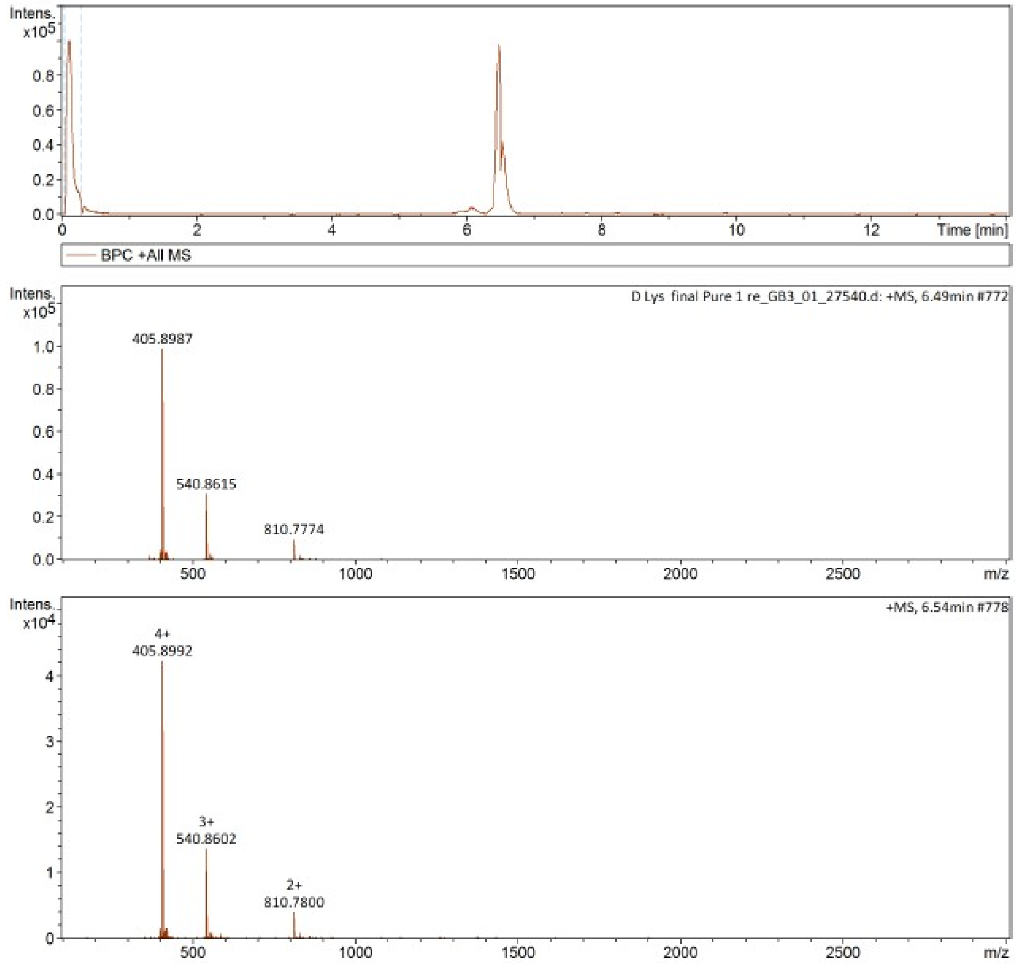

D Lys final Pure 1 re_GB3_01_27540.d

Bruker Compass DataAnalysis 4.3 printed: 6/24/2020 2:39:30 PM by: Thapelo Page 1 of 1

Figure S5: LC-MS spectrum of TP_ADLys conformation 1 (peak 1)

Display Report

Analysis Info		Acquisition Date	10/4/2019 6:10:52 PM	
Analysis Name	D:\Data\runs\thabo\lights\04-10-19\D Lys final Pure 2_GB3_01_27403.d			
Method	Wits HPLC high MS 100-3000 pos.m	Operator	Thapelo Mbhele	
Sample Name	D Lys final Pure 2	Instrument	compact	8255754.20116
Comment				

Acquisition Parameter

Source Type	ESI	Ion Polarity	Positive	Set Nebulizer	1.8 Bar
Focus	Not active	Set Capillary	4500 V	Set Dry Heater	220 °C
Scan Begin	100 m/z	Set End Plate Offset	-500 V	Set Dry Gas	9.0 l/min
Scan End	3000 m/z	Set Charging Voltage	2000 V	Set Divert Valve	Waste
		Set Corona	0 nA	Set APCI Heater	0 °C

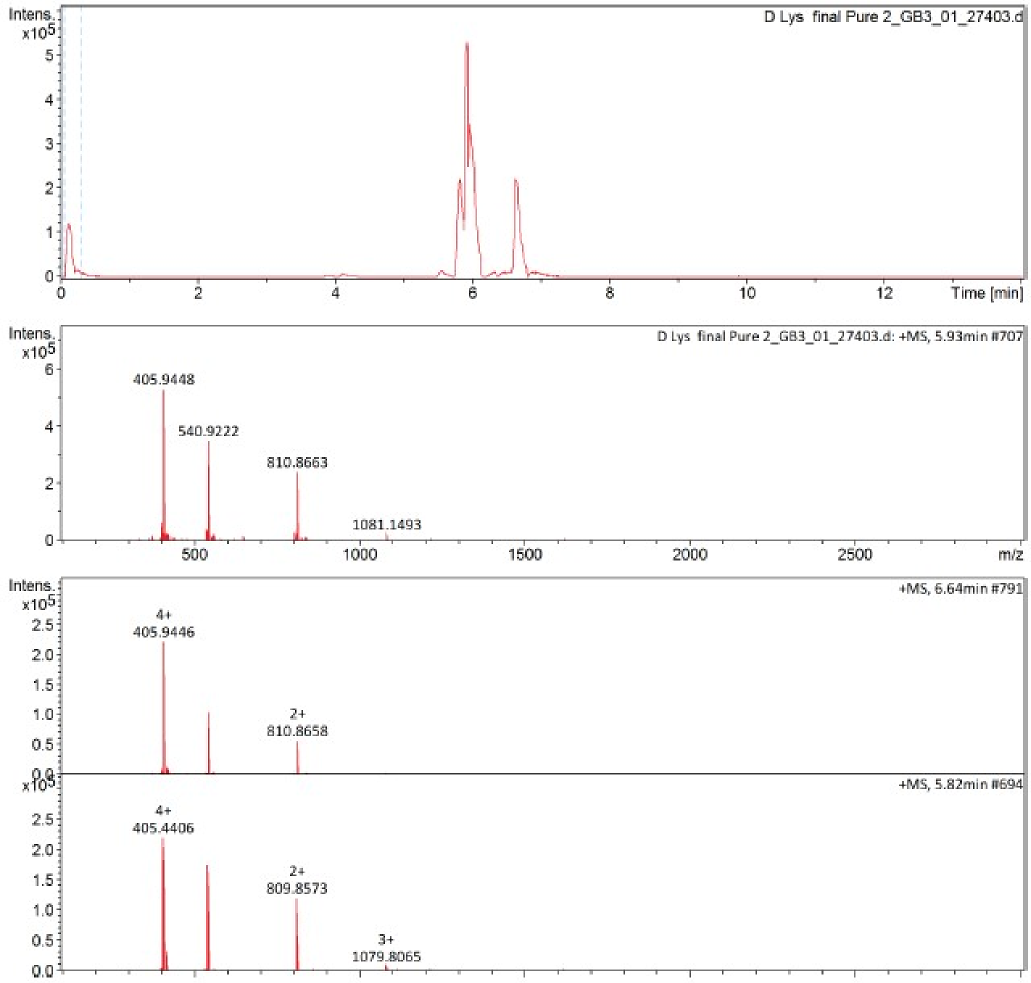

D Lys final Pure 2_GB3_01_27403.d

Bruker Compass DataAnalysis 4.3 printed: 6/24/2020 2:44:46 PM by: Thapelo Page 1 of 1

Figure S6: LC-MS spectrum of TP_ADLys conformation 2 (peak 2)

Display Report

Analysis Info

Analysis Name	D:\Data\runs\thabo\lights\04-10-19\D Lys final Pure 3_GB2_01_27394.d	Acquisition Date	10/4/2019 2:50:04 PM
Method	Wits HPLC high MS 100-3000 pos.m	Operator	Thapelo Mbhele
Sample Name	D Lys final Pure 3	Instrument	compact 8255754.20116
Comment			

Acquisition Parameter

Source Type	ESI	Ion Polarity	Positive	Set Nebulizer	1.8 Bar
Focus	Not active	Set Capillary	4500 V	Set Dry Heater	220 °C
Scan Begin	100 m/z	Set End Plate Offset	-500 V	Set Dry Gas	9.0 l/min
Scan End	3000 m/z	Set Charging Voltage	2000 V	Set Divert Valve	Waste
		Set Corona	0 nA	Set APCI Heater	0 °C

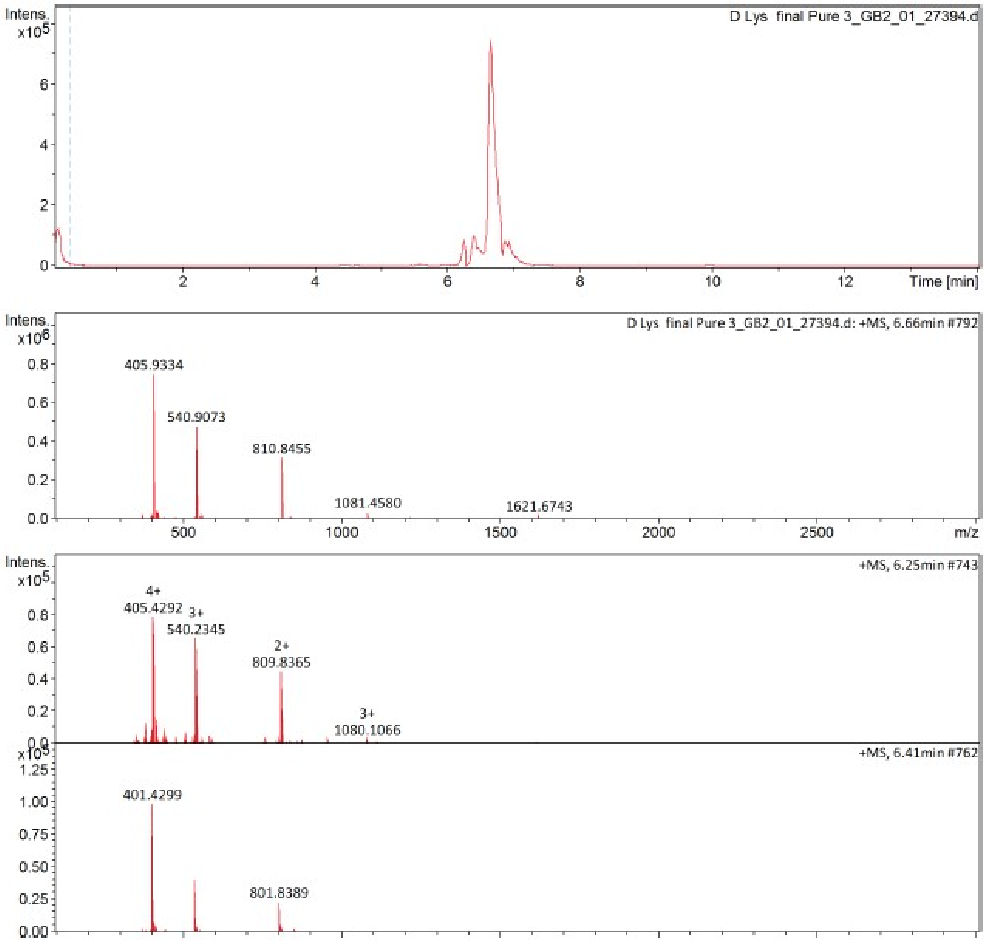

D Lys final Pure 3_GB2_01_27394.d

Bruker Compass DataAnalysis 4.3 printed: 6/24/2020 3:11:22 PM by: Thapelo Page 1 of 1

Figure S7: LC-MS spectrum of TP_ADLys conformation 3 (peak 3)

Display Report

Analysis Info

		Acquisition Date	10/4/2019 6:38:23 PM
Analysis Name	D:\Data\runs\thabo\lights\04-10-19\D Lys final Pure 4_GB4_01_27405.d		
Method	Wits HPLC high MS 100-3000 pos.m	Operator	Thapelo Mbhele
Sample Name	D Lys final Pure 4	Instrument	compact 8255754.20116
Comment			

Acquisition Parameter

Source Type	ESI	Ion Polarity	Positive	Set Nebulizer	1.8 Bar
Focus	Not active	Set Capillary	4500 V	Set Dry Heater	220 °C
Scan Begin	100 m/z	Set End Plate Offset	-500 V	Set Dry Gas	9.0 l/min
Scan End	3000 m/z	Set Charging Voltage	2000 V	Set Divert Valve	Waste
		Set Corona	0 nA	Set APCI Heater	0 °C

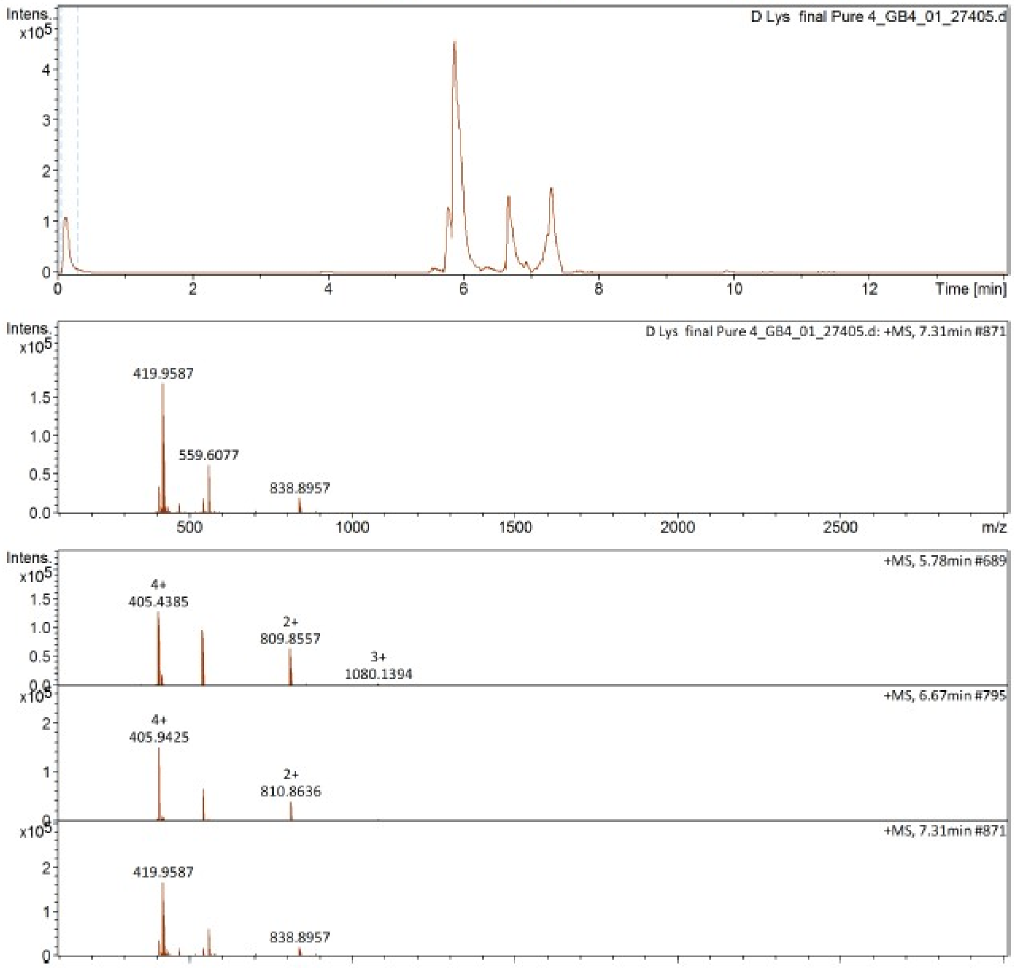

D Lys final Pure 4_GB4_01_27405.d

Bruker Compass DataAnalysis 4.3 printed: 6/24/2020 3:19:39 PM by: Thapelo Page 1 of 1

Figure S8: LC-MS spectrum of TP_ADLys conformation 3 (peak 3)

6. NMR spectra for peptide structures

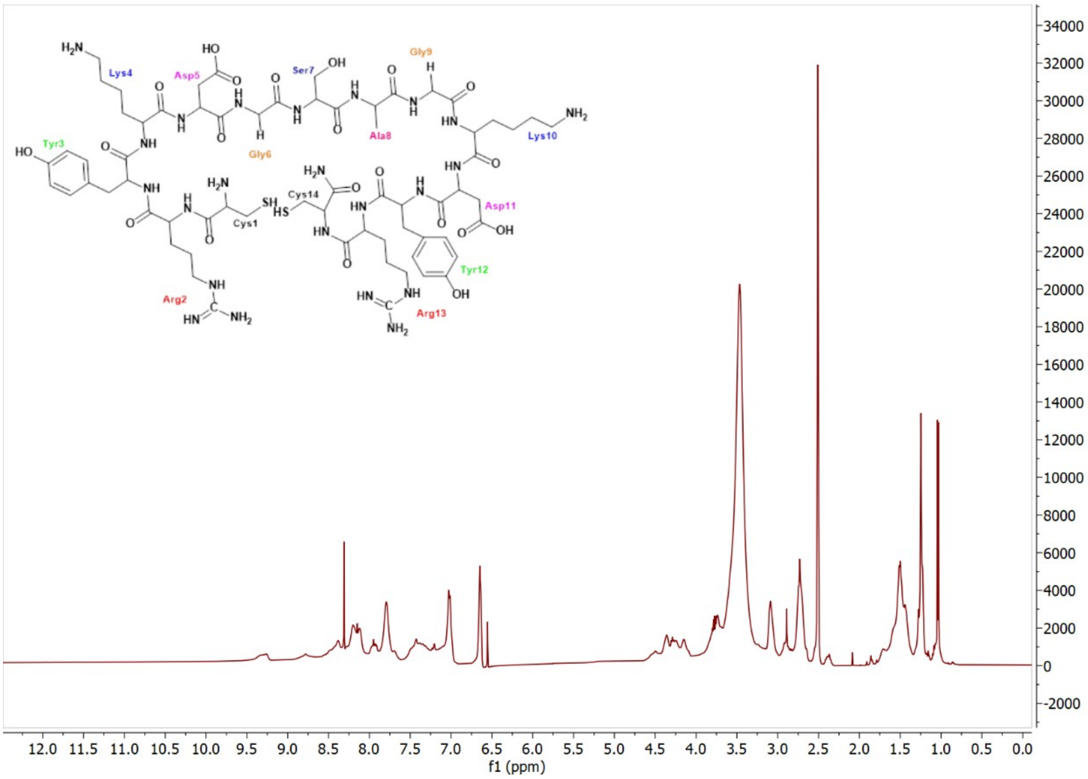

Figure S9: ^{1}H NMR spectrum of TP_Asp peptide

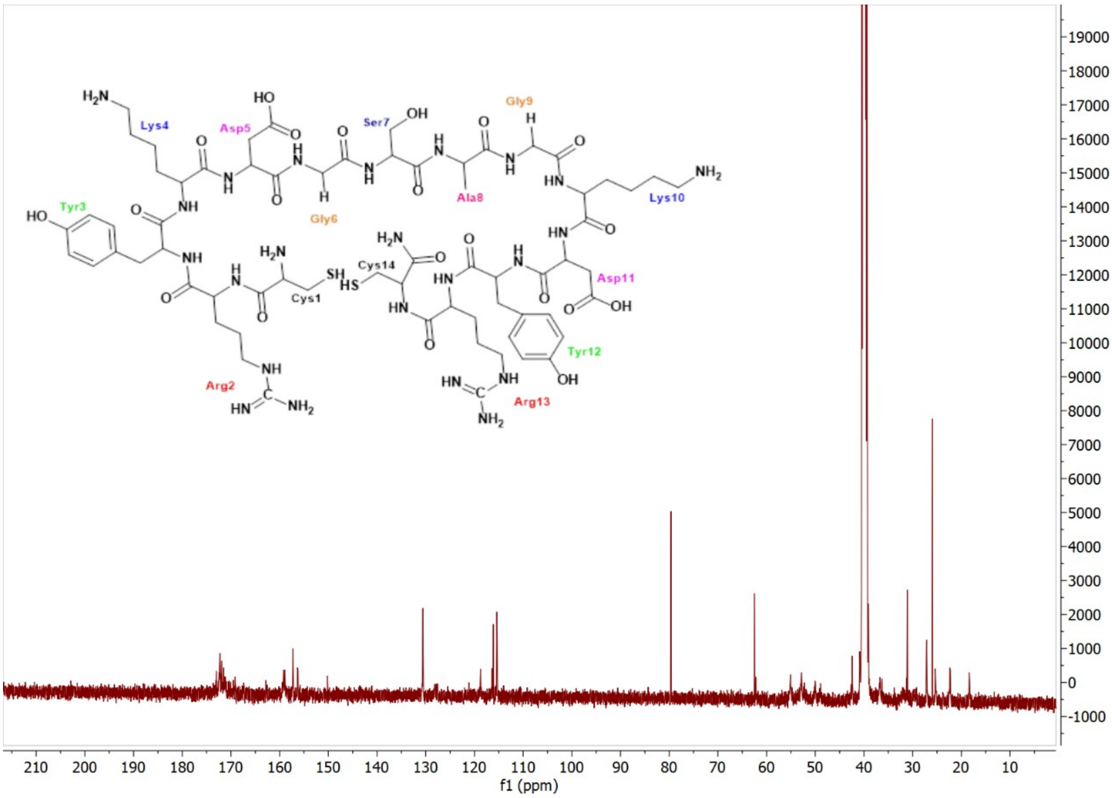

Figure S.2-9: ^{13}C NMR spectrum of TP_Asp peptide

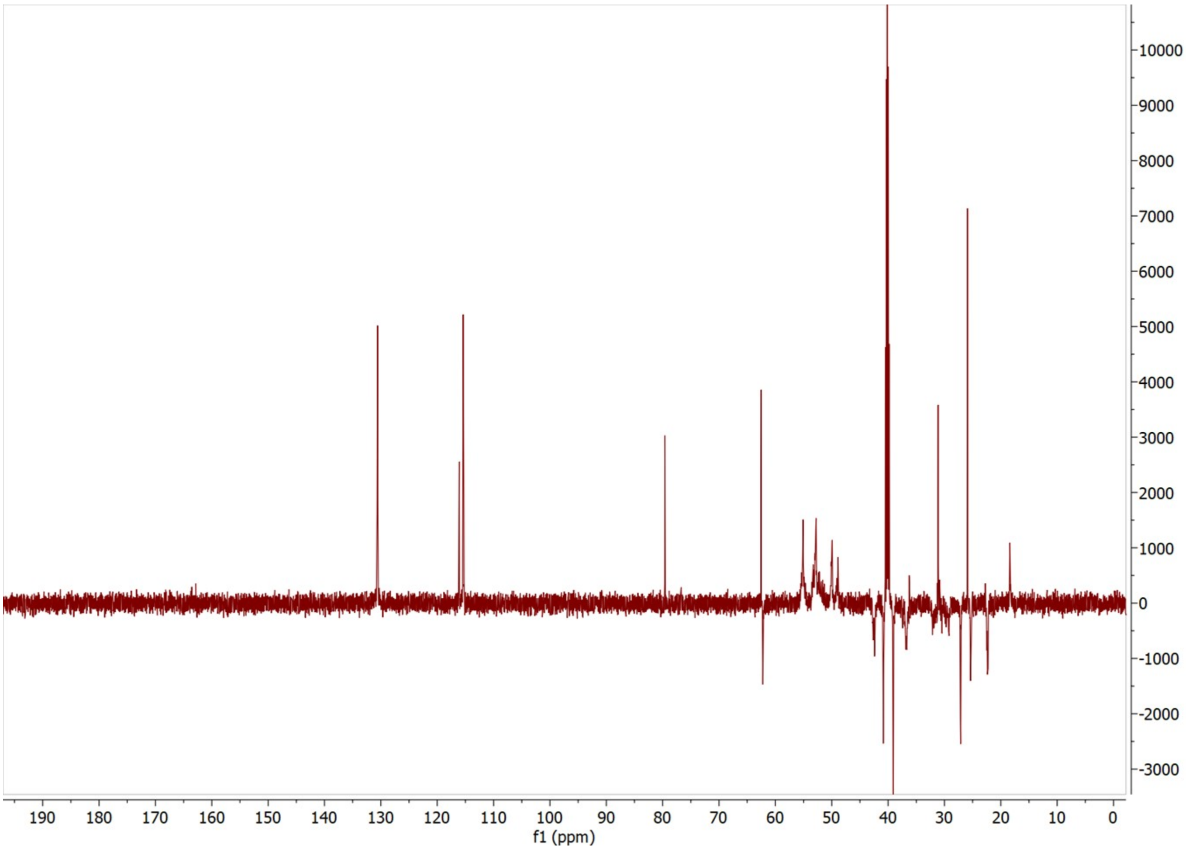

Figure S10: DEPT 135 NMR spectrum of TP_Asp peptide

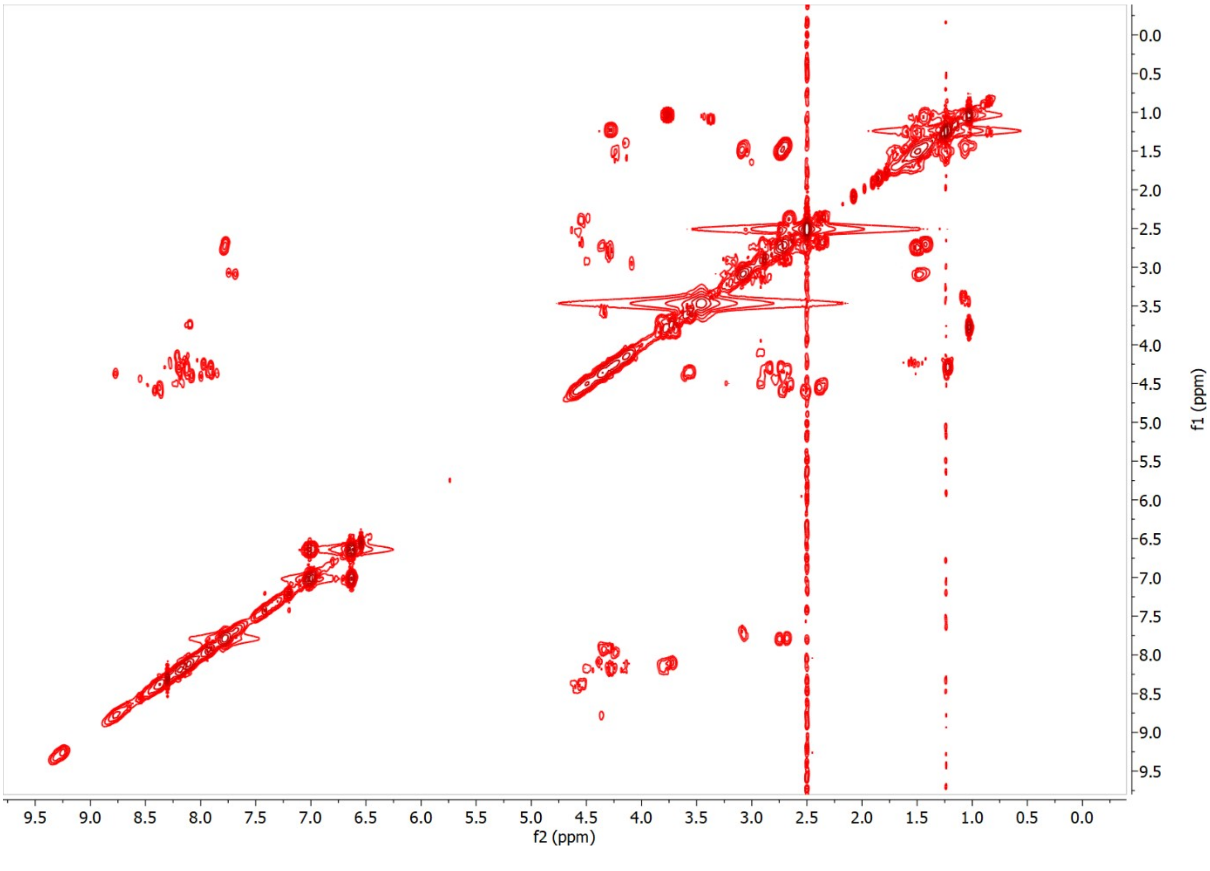

Figure S11: 2D ^{1}H COSY NMR spectrum of TP_Asp peptide

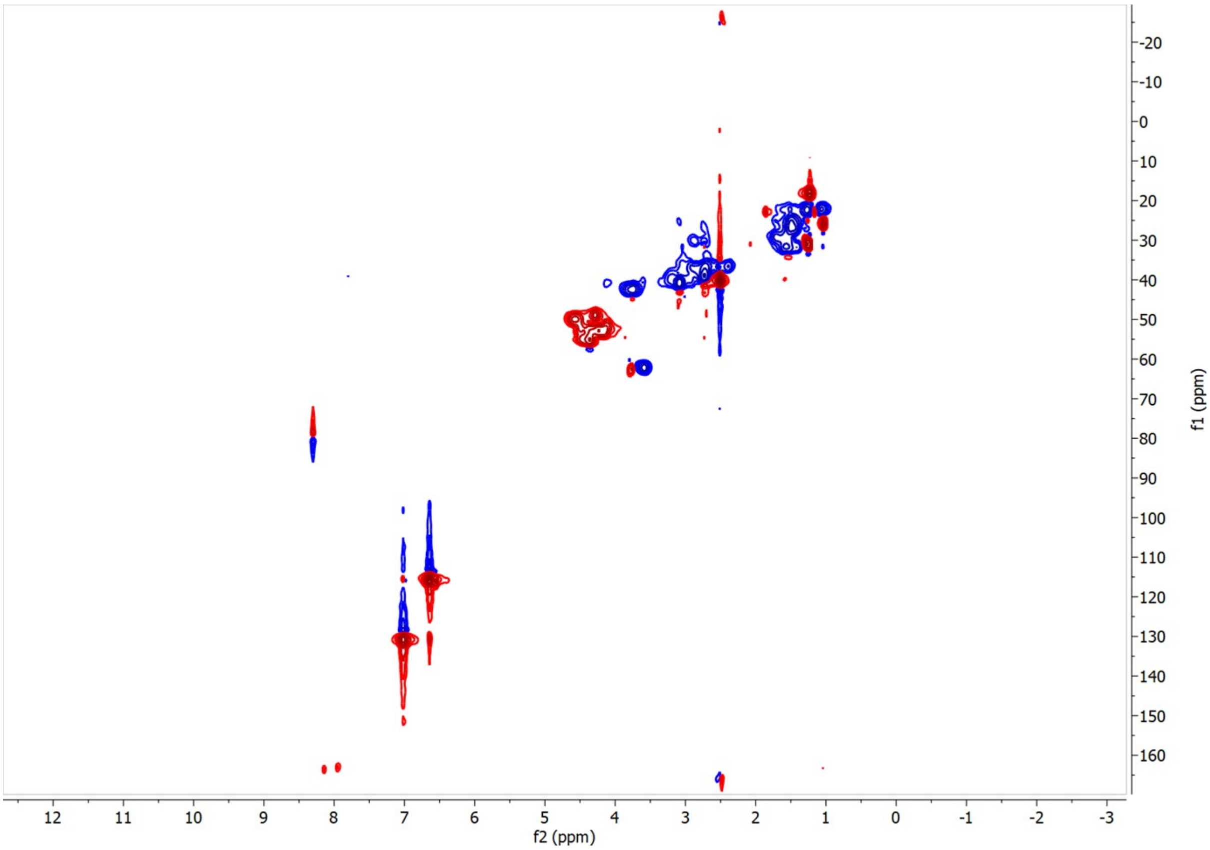

Figure S12: HSQC NMR spectrum of TP_Asp peptide

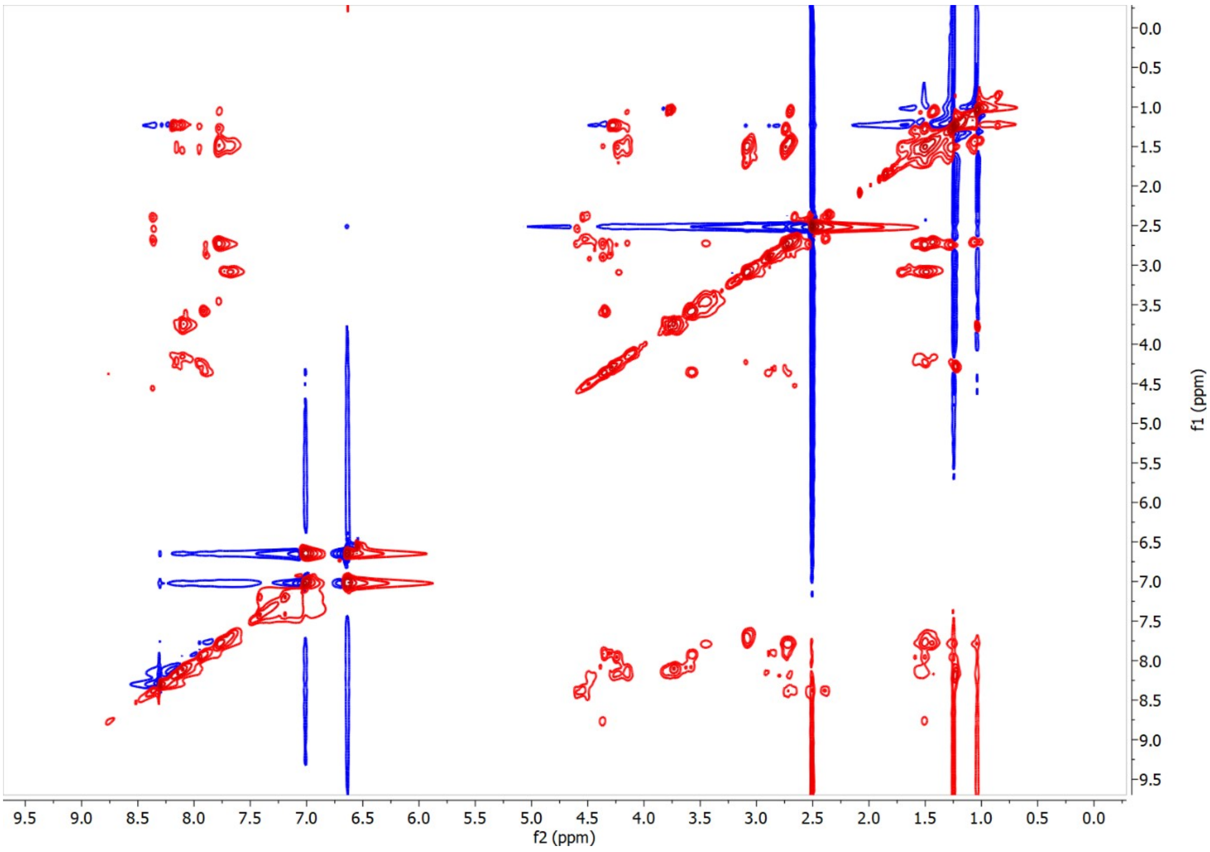

Figure S13: 2D ^{1}H TOCSY NMR spectrum of TP_Asp peptide

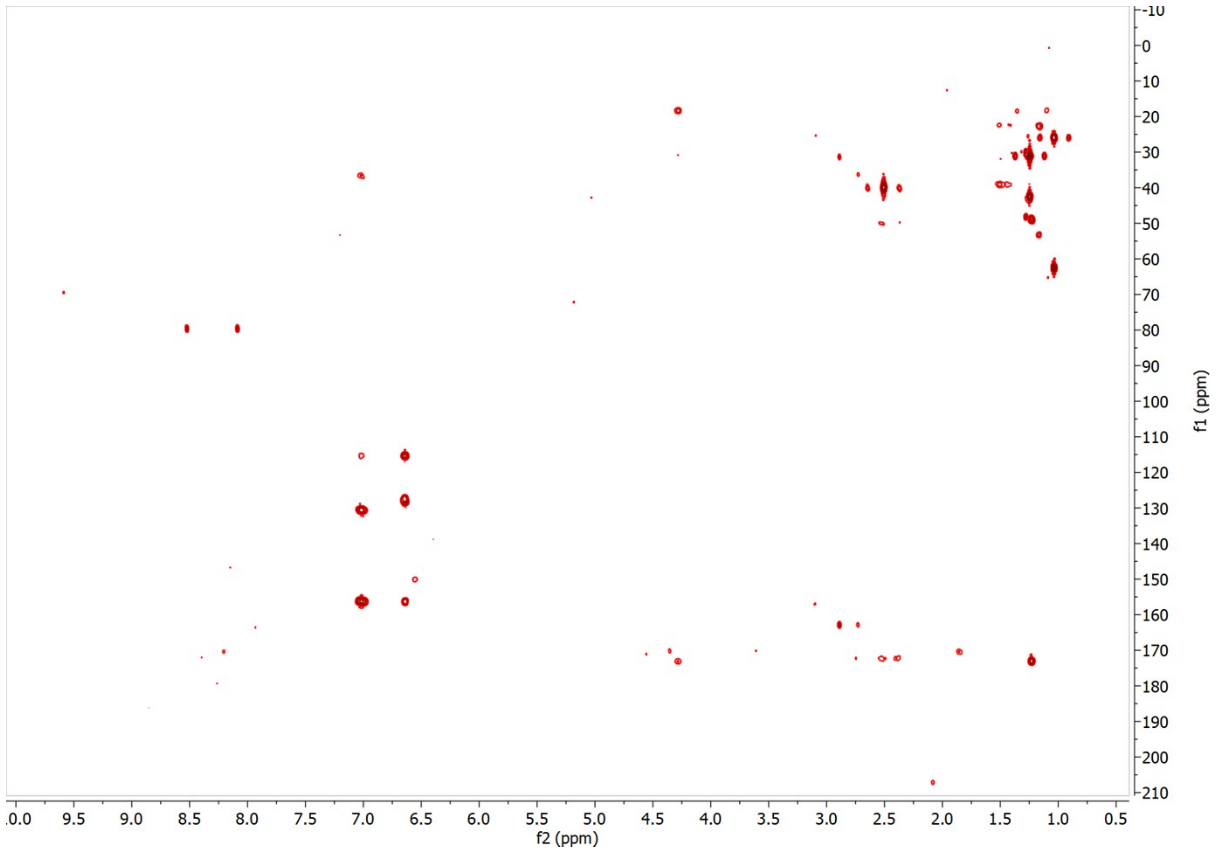

Figure S14: HMBC NMR spectrum of TP_Asp peptide

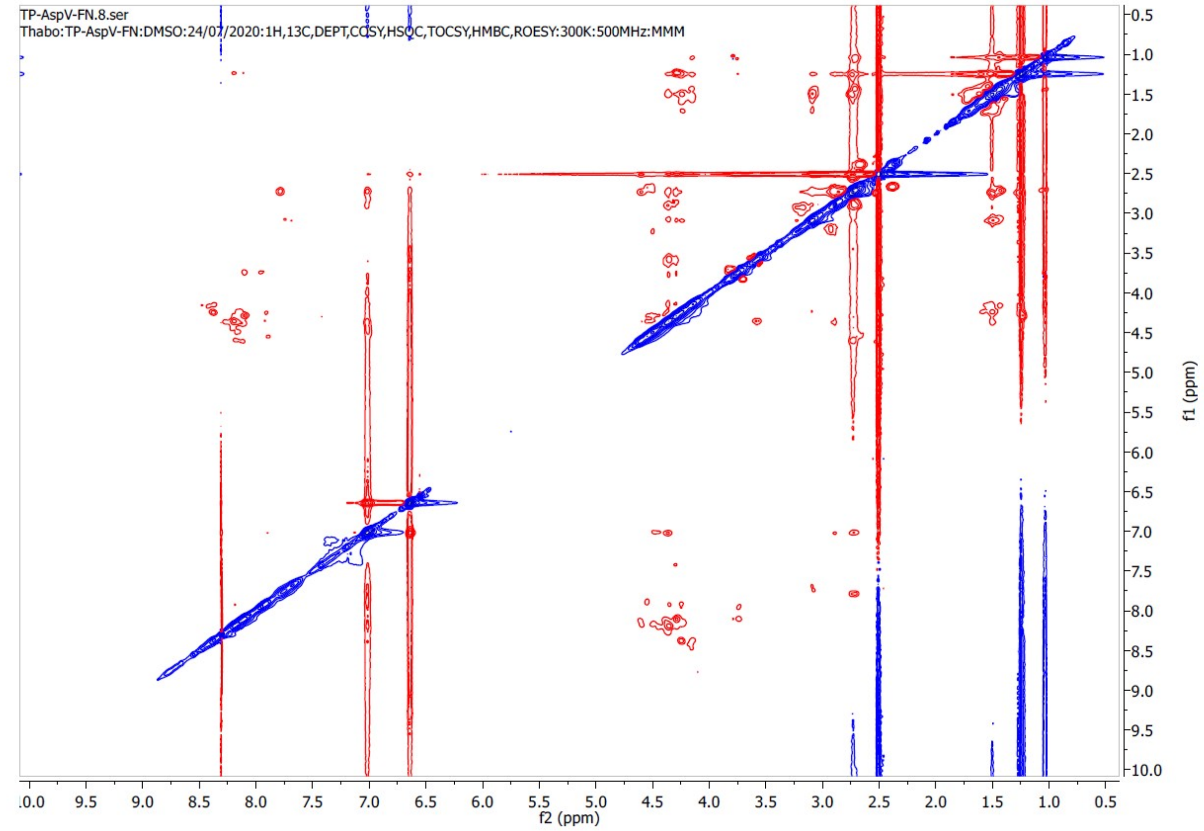

Figure S15: 2D ^{1}H ROESY NMR spectrum of TP_Asp peptide

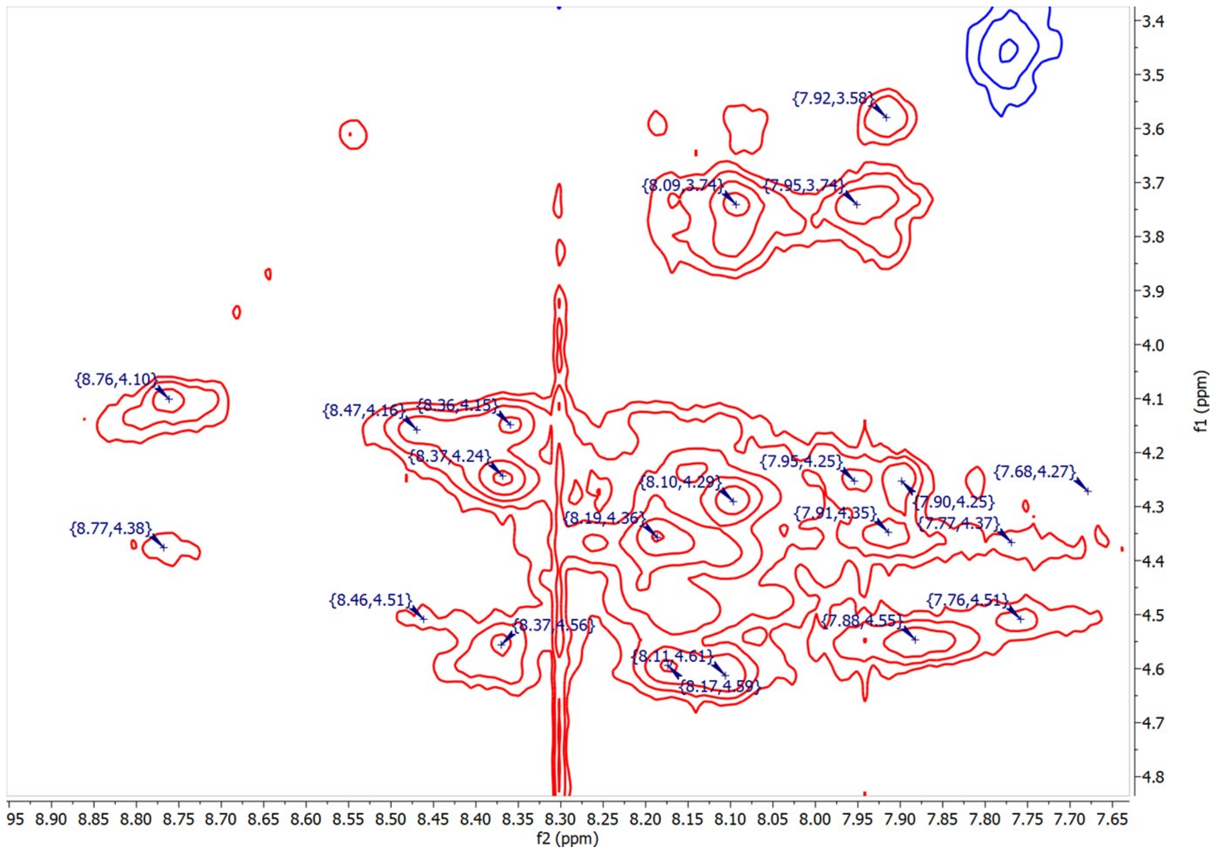

Figure S16: 2D ^{1}H ROESY NMR spectrum of TP_ADLys peptide, ^{1}HN region

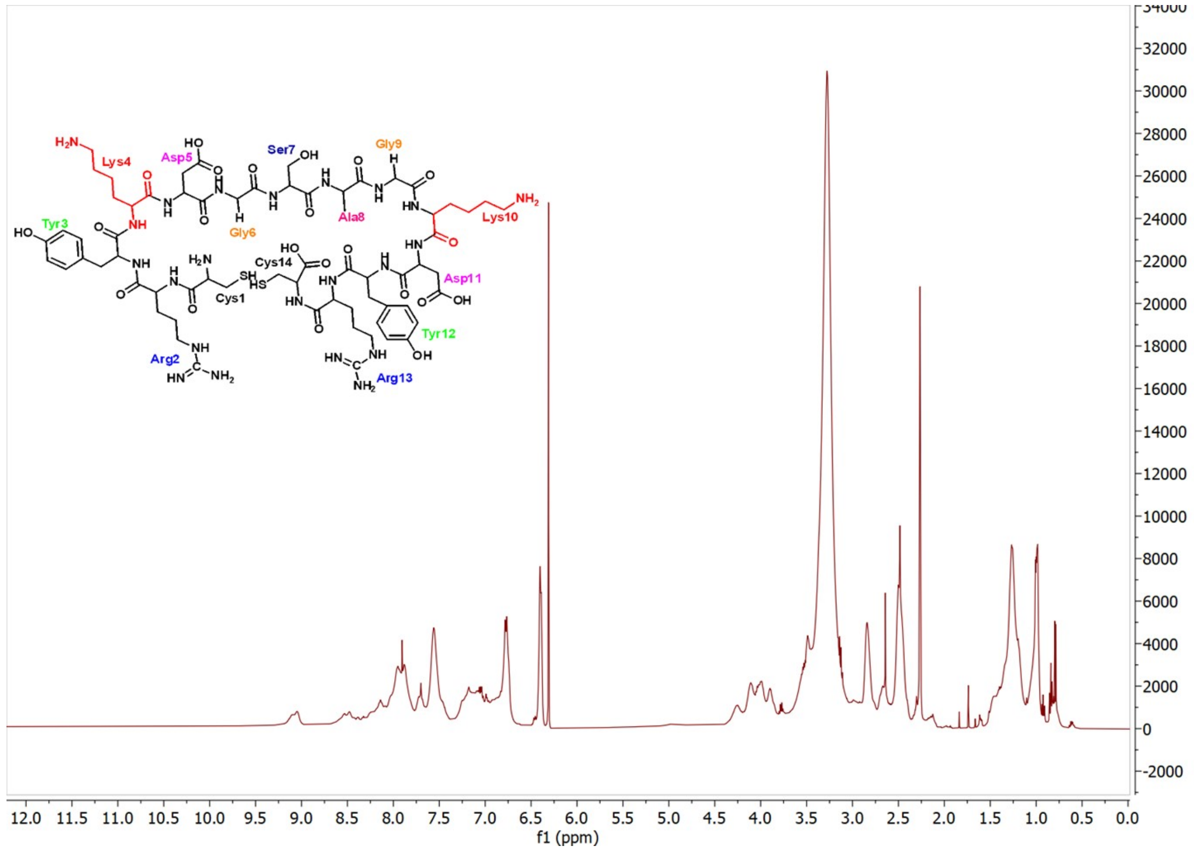

Figure S17: ^{1}H NMR spectrum of TP_ADLys peptide

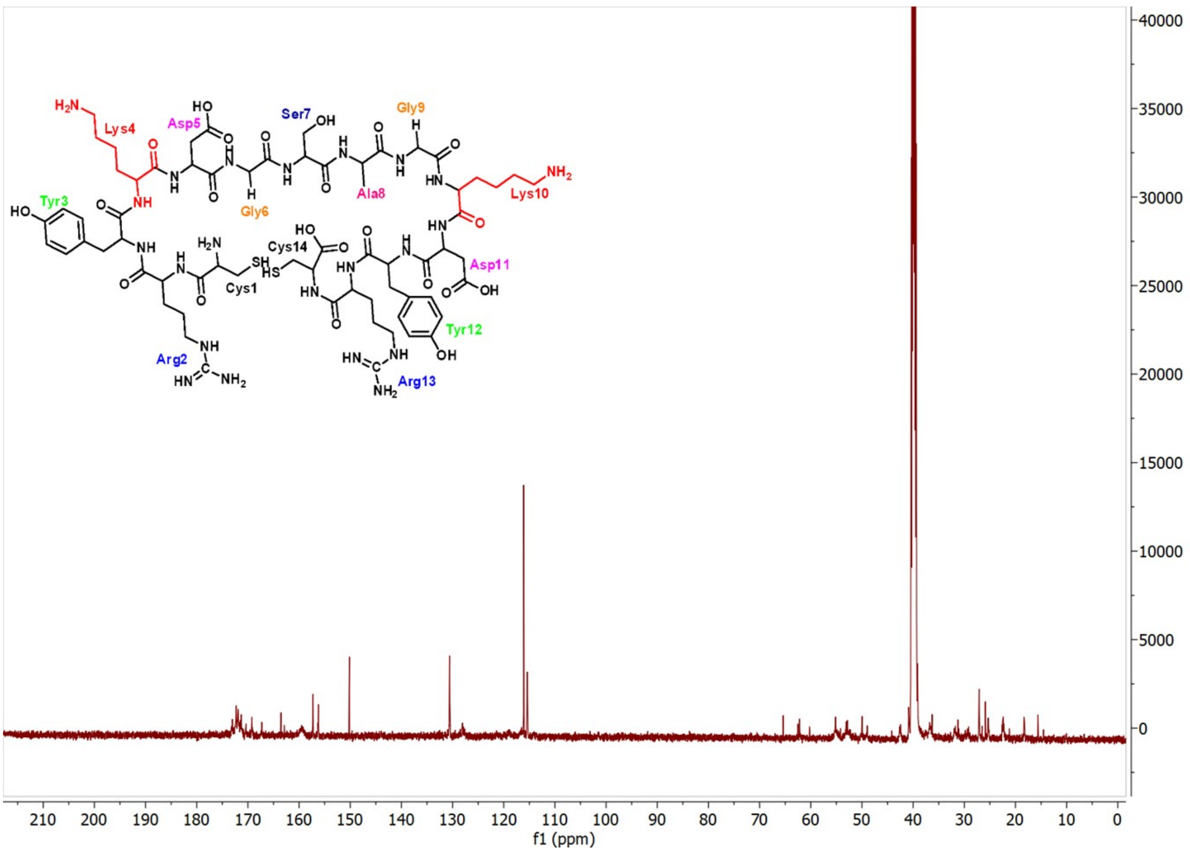

Figure S18: ^{13}C NMR spectrum of TP_ADLys peptide

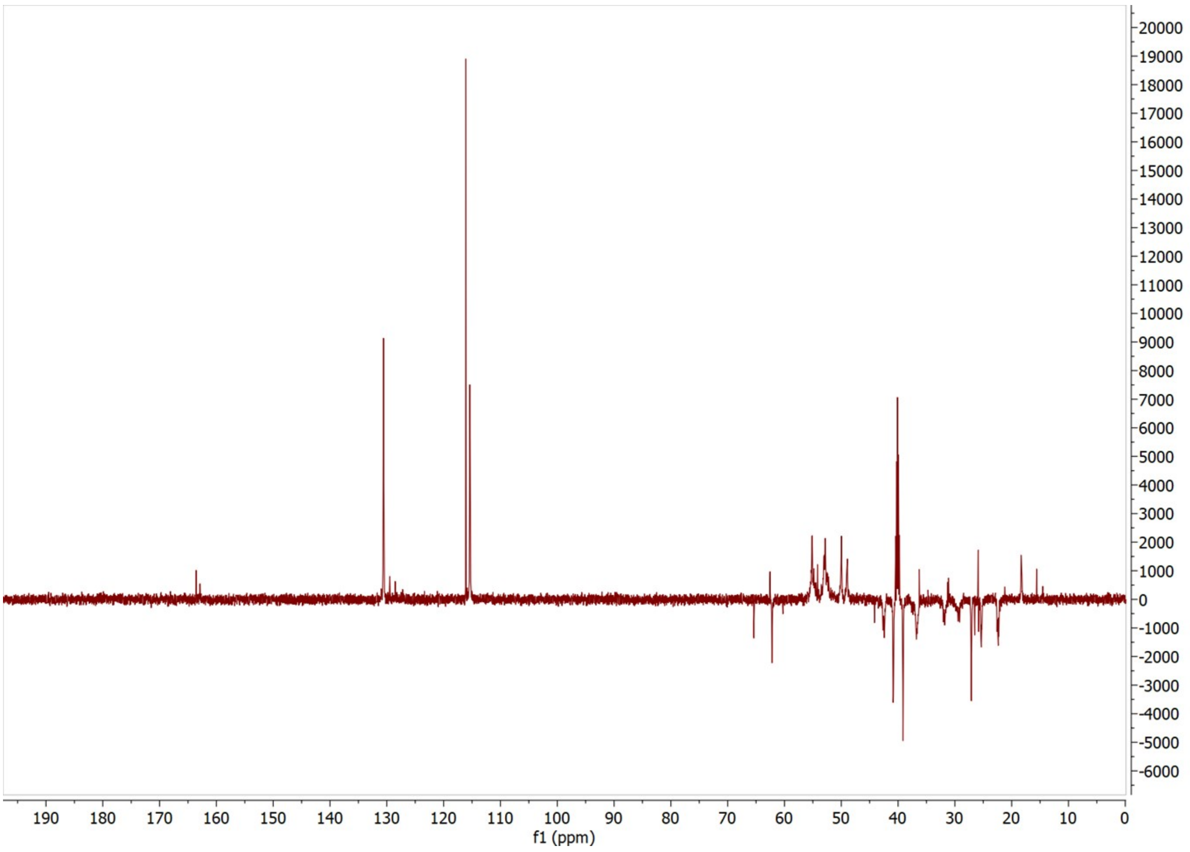

Figure S19: DEPT 135 NMR spectrum of TP_ADLys peptide

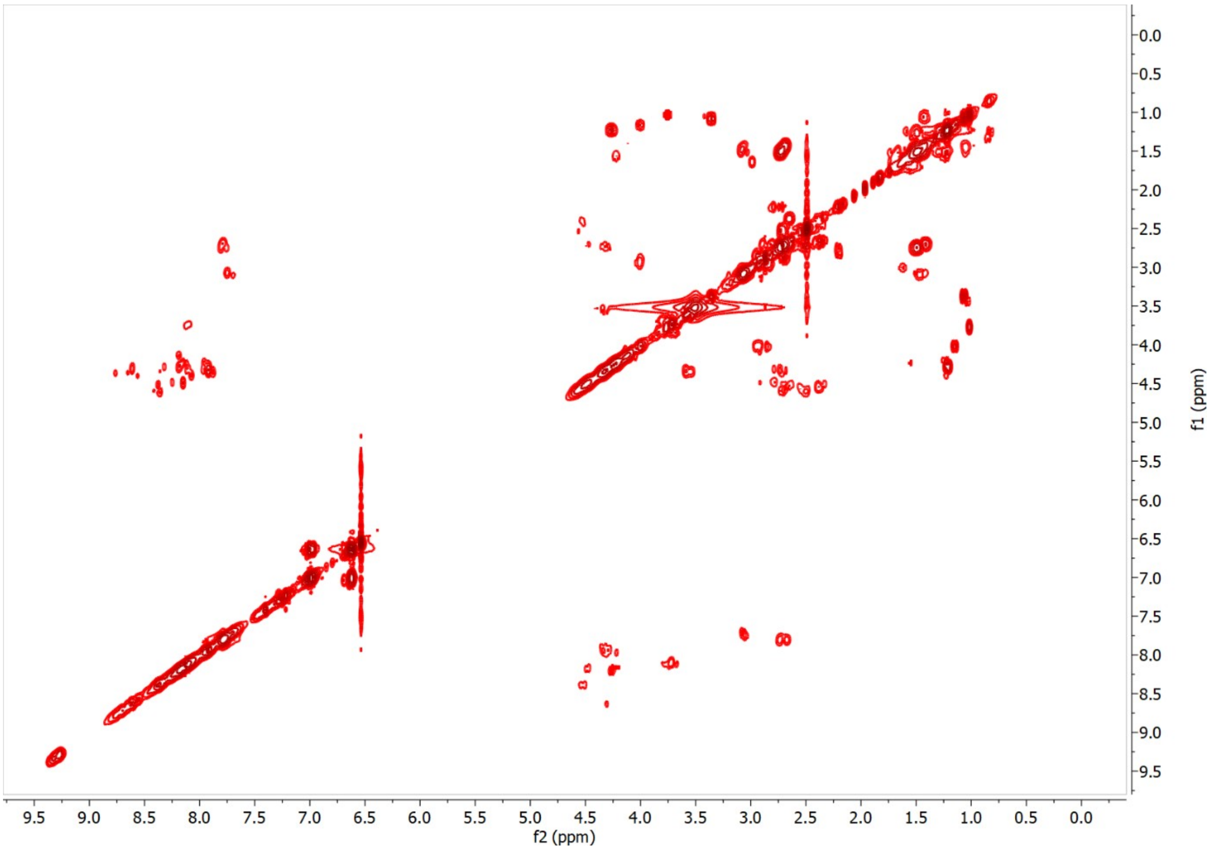

Figure S20: 2D ^{1}H COSY NMR spectrum of TP_ADLys peptide

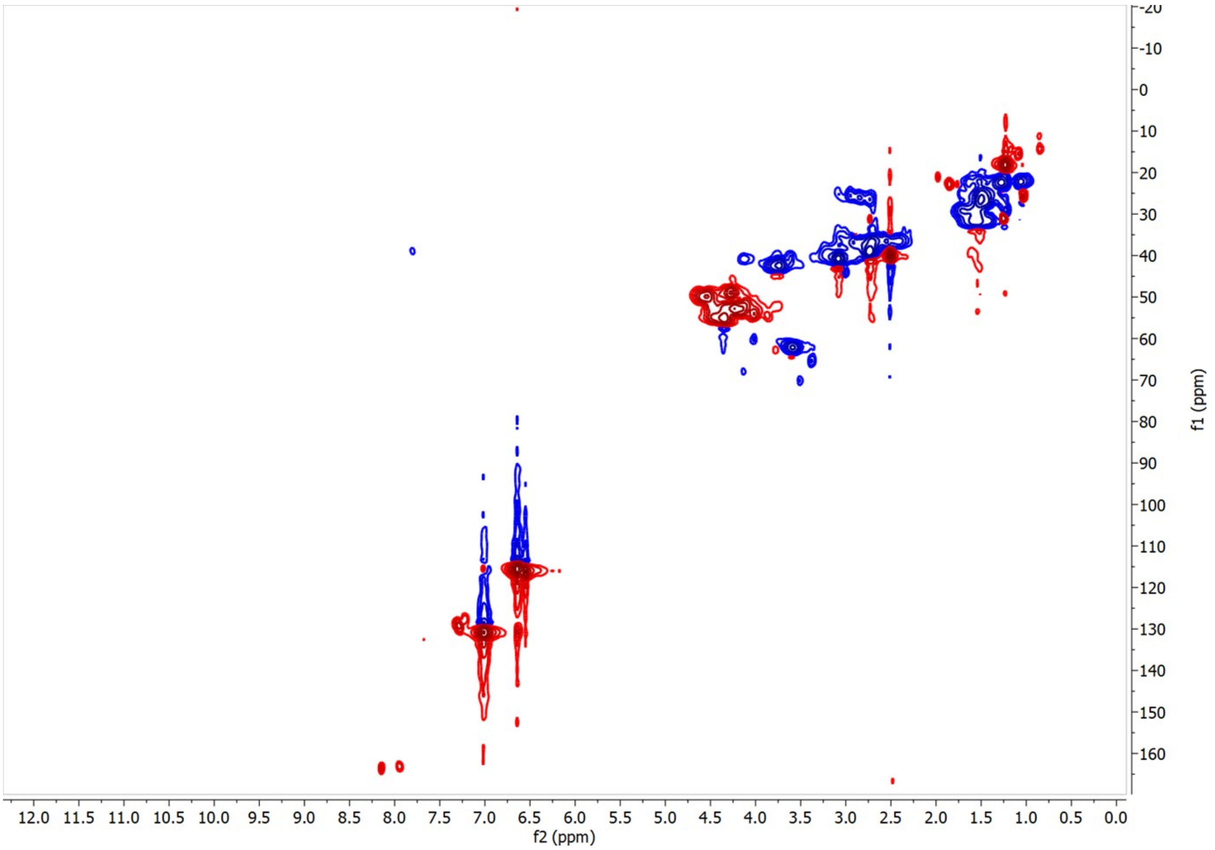

Figure S21: HSQC NMR spectrum of TP_ADLys peptide

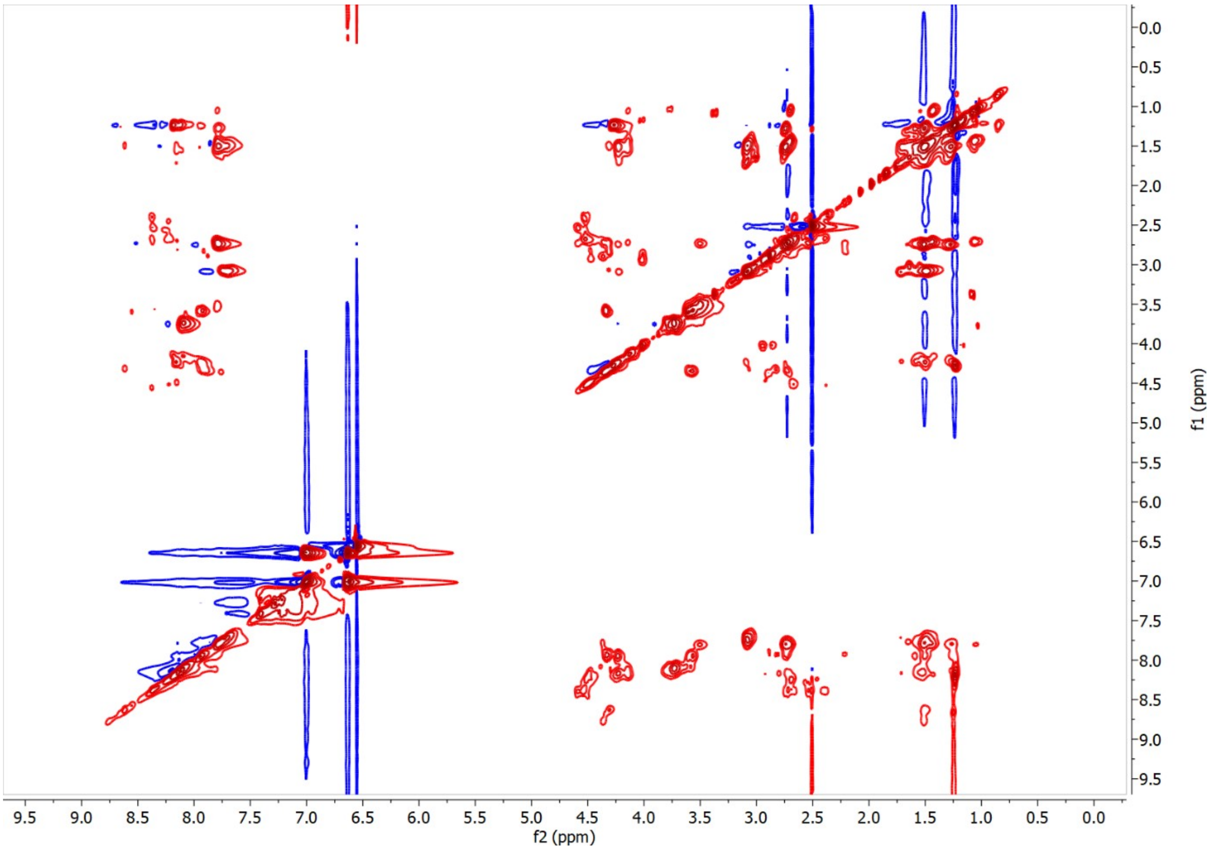

Figure S22: 2D ^{1}H TOCSY spectrum of TP_ADLys peptide

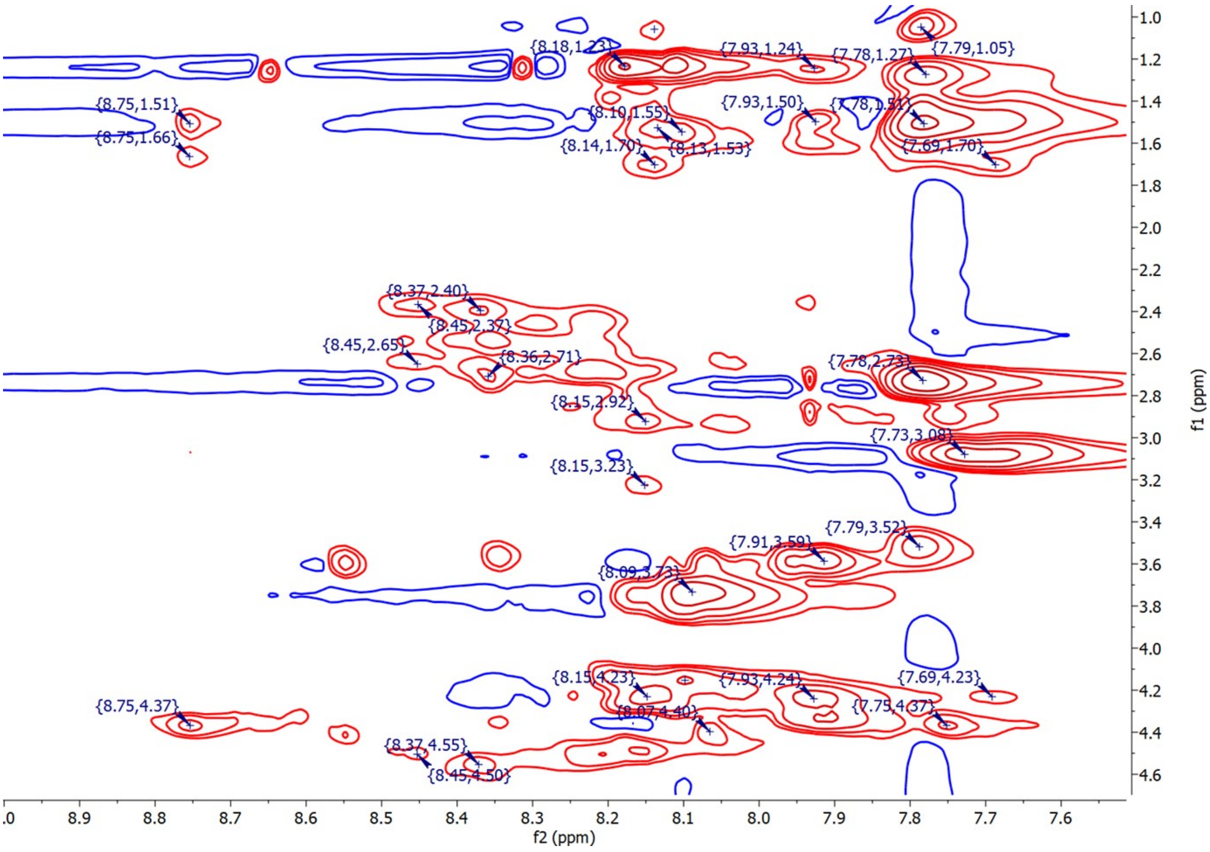

Figure S23: 2D ^{1}H TOCSY spectrum of TP_ADLys peptide, ^{1}HN fingerprint region

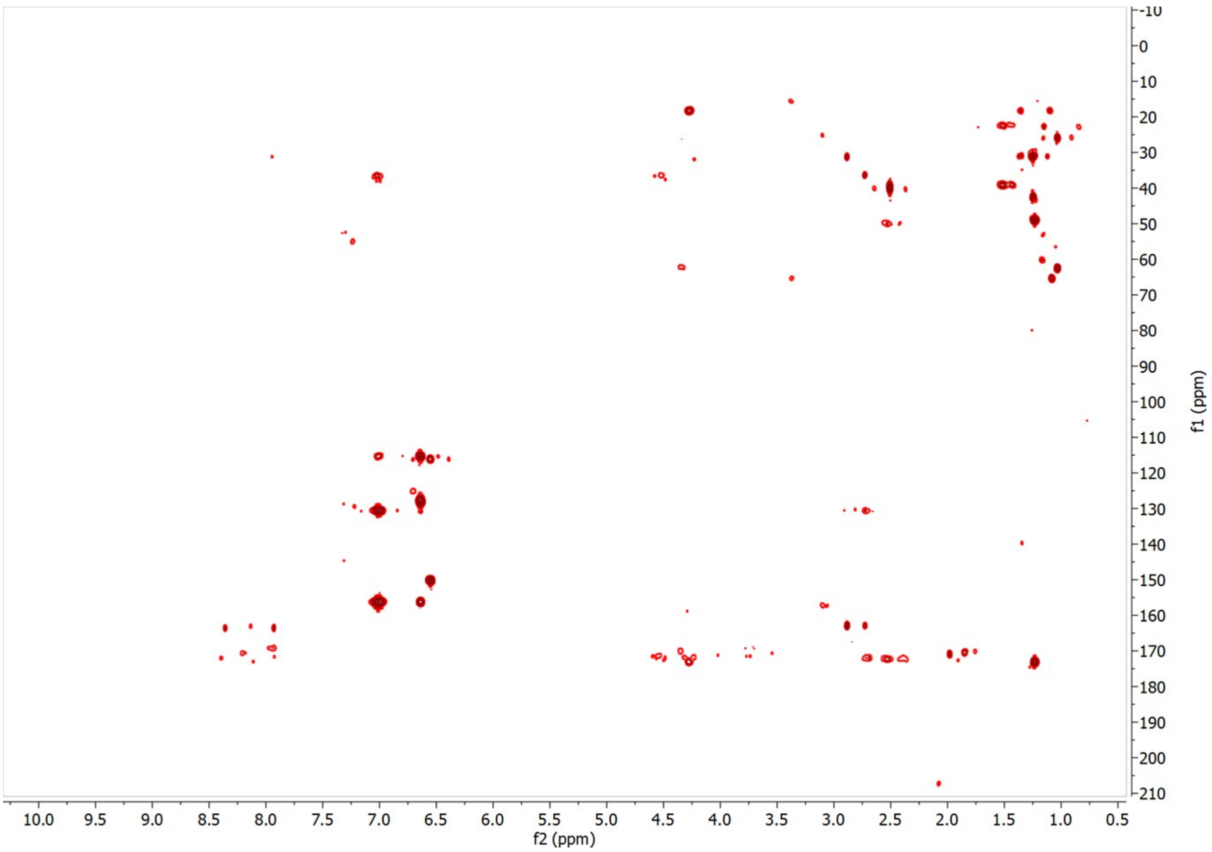

Figure S24: HMBC NMR spectrum of TP_ADLys peptide

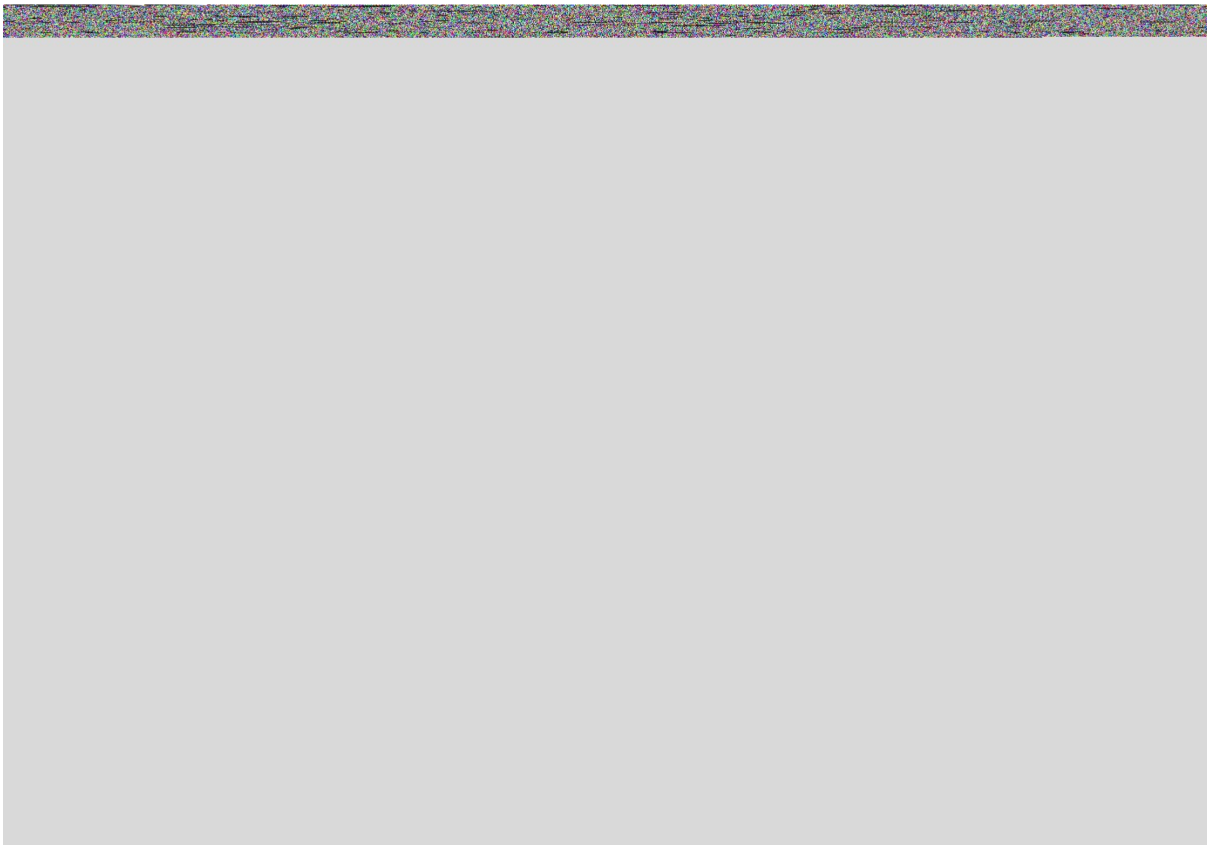

Figure S25: 2D ^{1}H ROESY NMR spectrum of TP_ADLys peptide

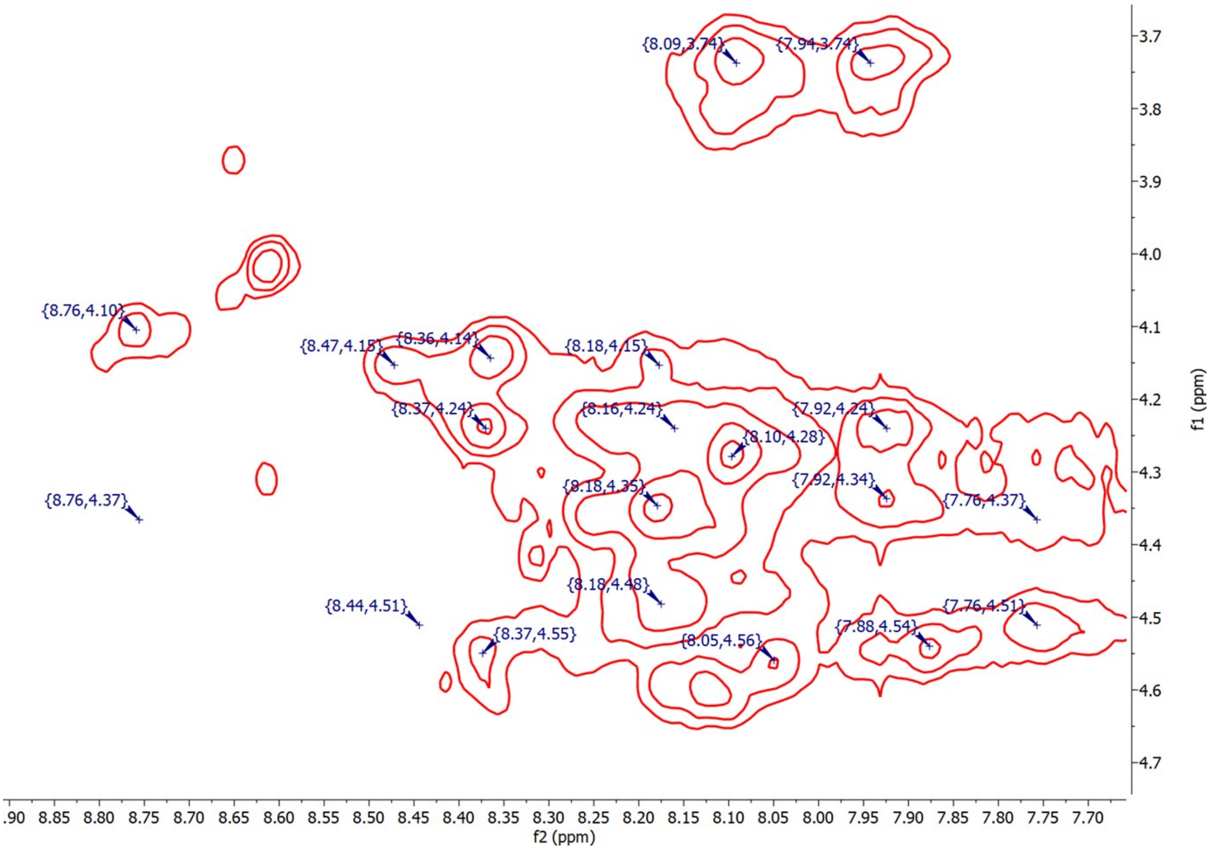

Figure S26: 2D ^{1}H ROESY NMR spectrum of TP_ADLys peptide, ^{1}HN region

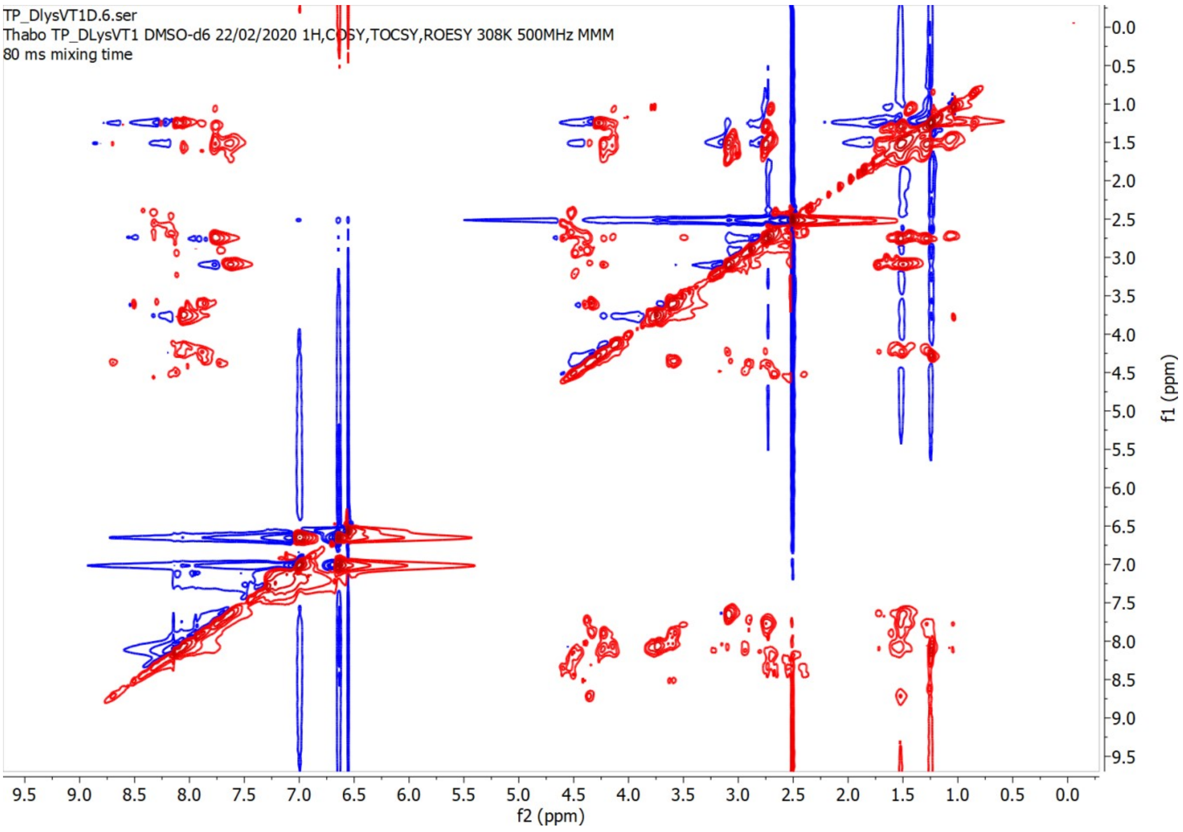

Figure S27: 2D ^{1}H TOCSY spectrum of TP_ADLys peptide at **308K**

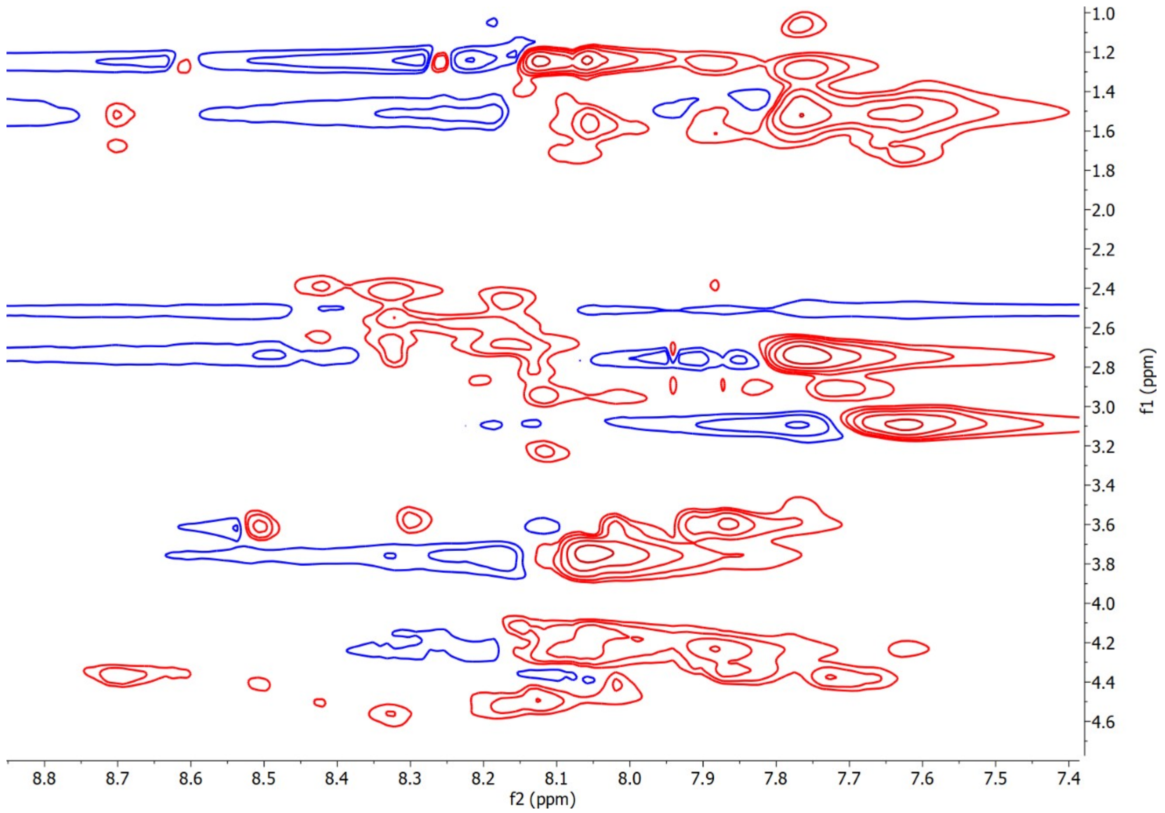

Figure S28: 2D ^{1}H TOCSY spectrum of TP_ADLys peptide, ^{1}HN fingerprint region at 308K

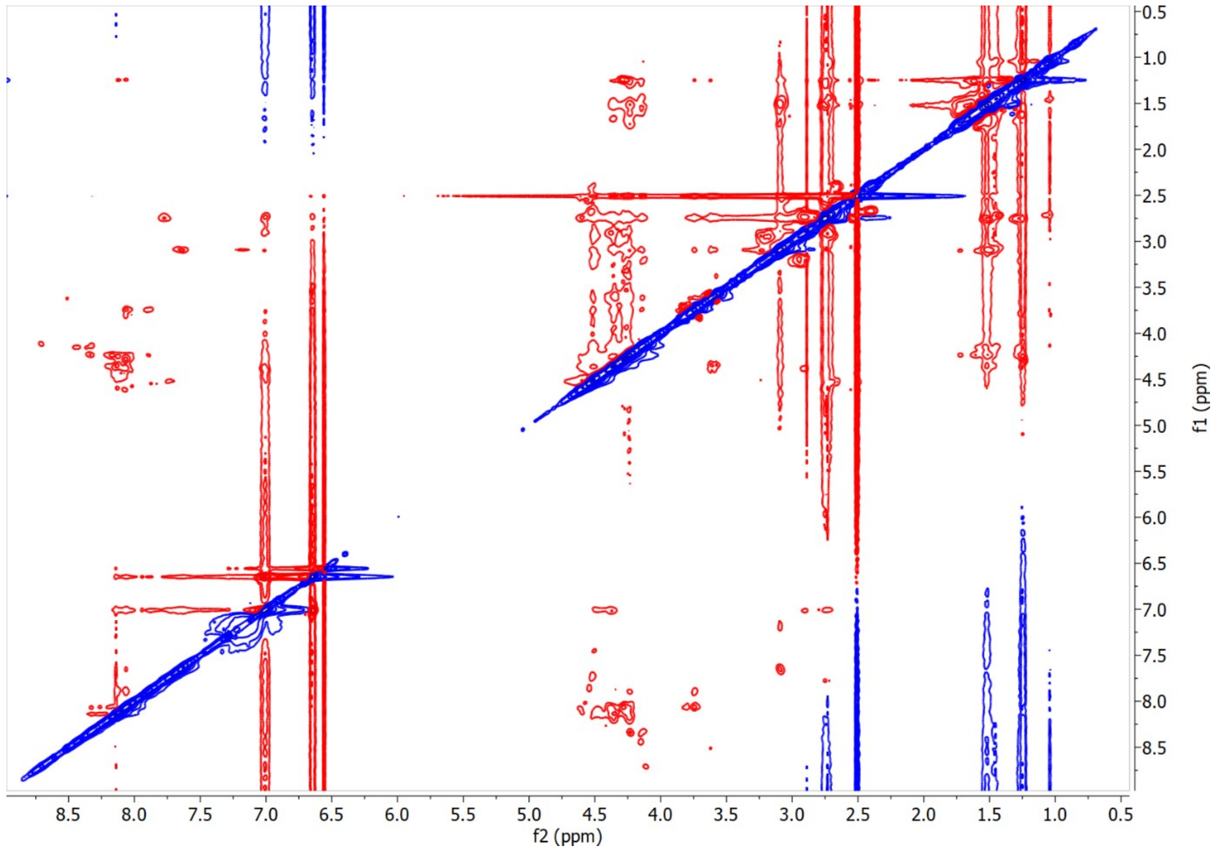

Figure-S29: 2D ^{1}H ROESY spectrum of TP_ADLys peptide at **308K**

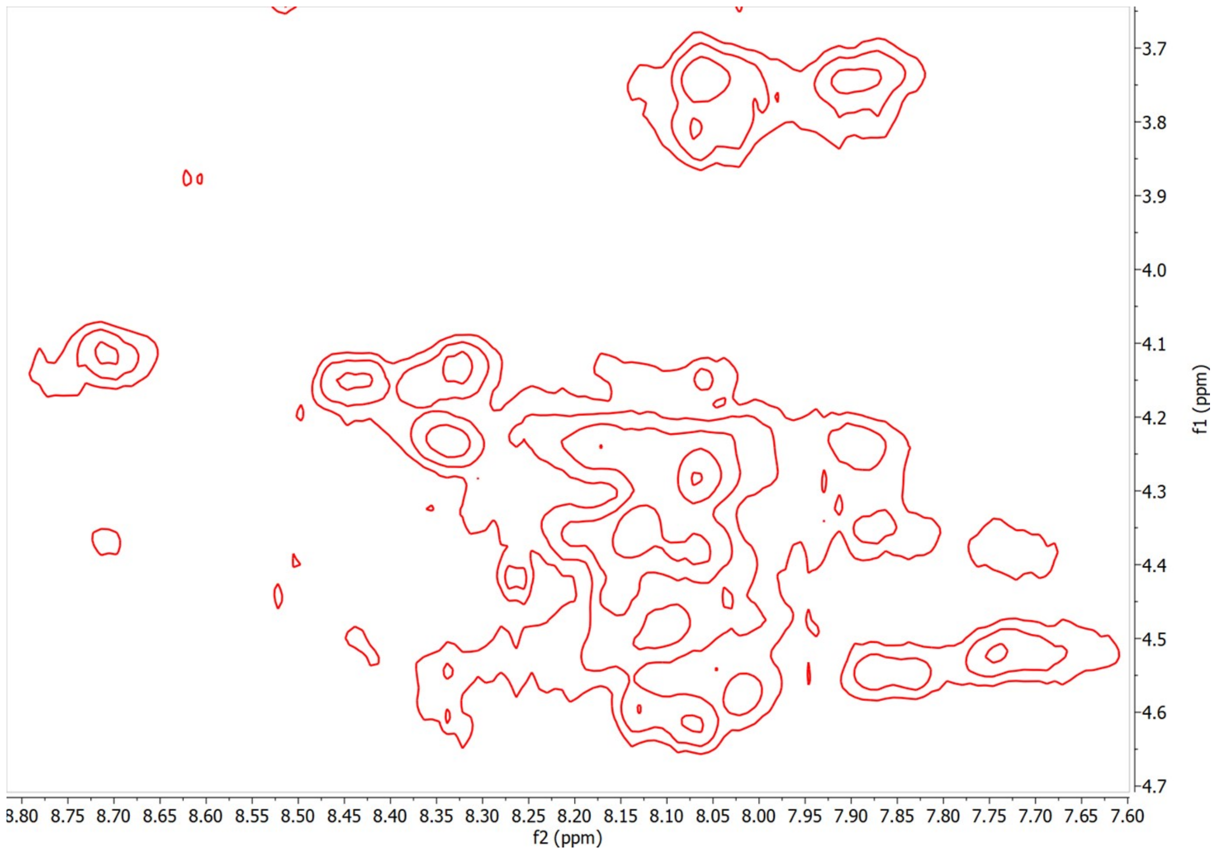

Figure S30: 2D ^{1}H ROESY spectrum of TP_ADLys peptide, ^{1}HN fingerprint region at **308K.**

Selected NMR spectra for aldol products

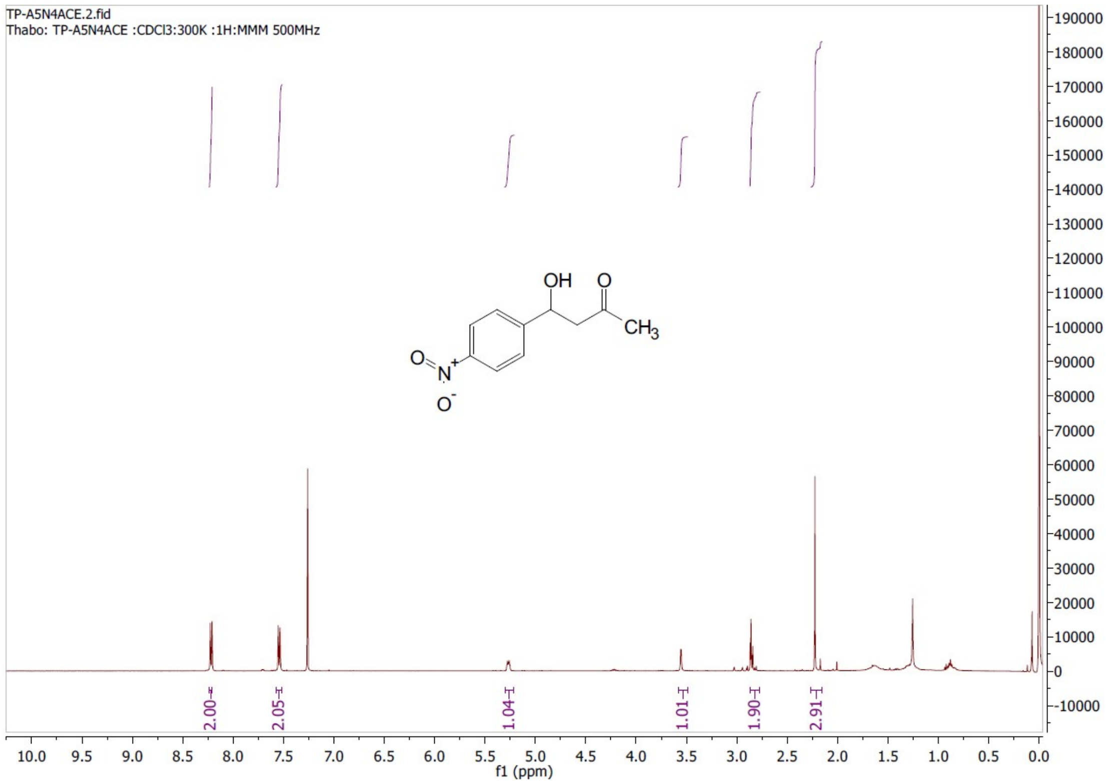

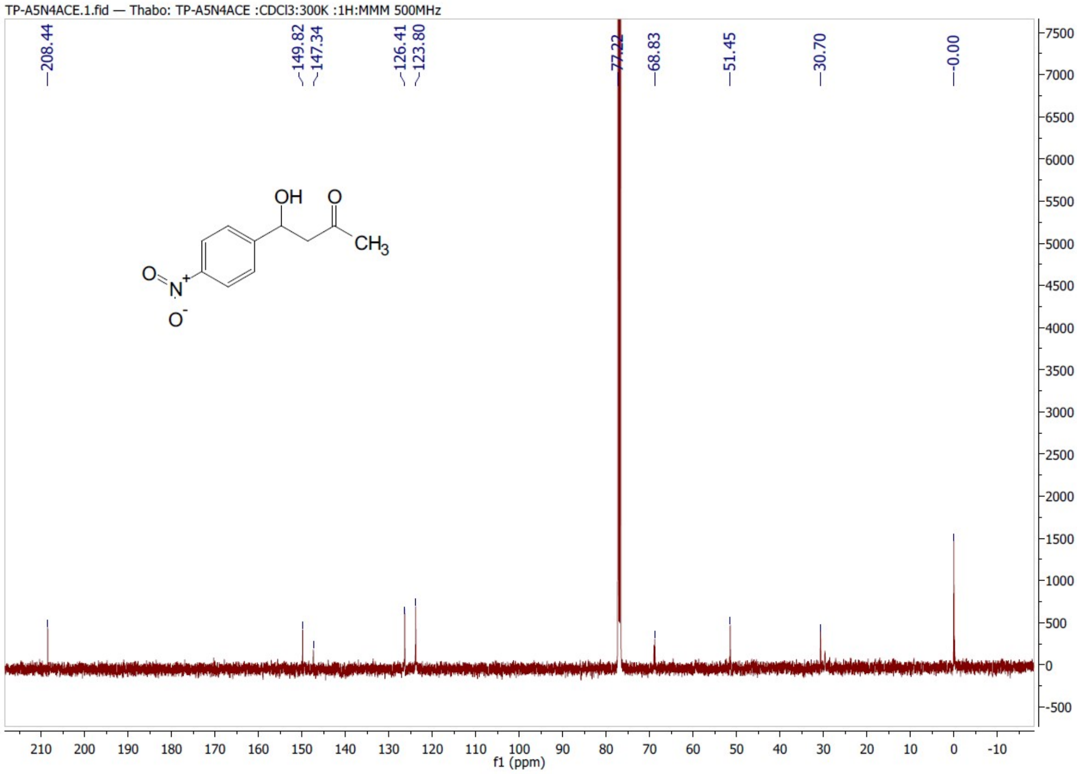

Figure S31: ^{1}H and ^{13}C NMR spectra of the aldol product **TP_A1N4ACE**

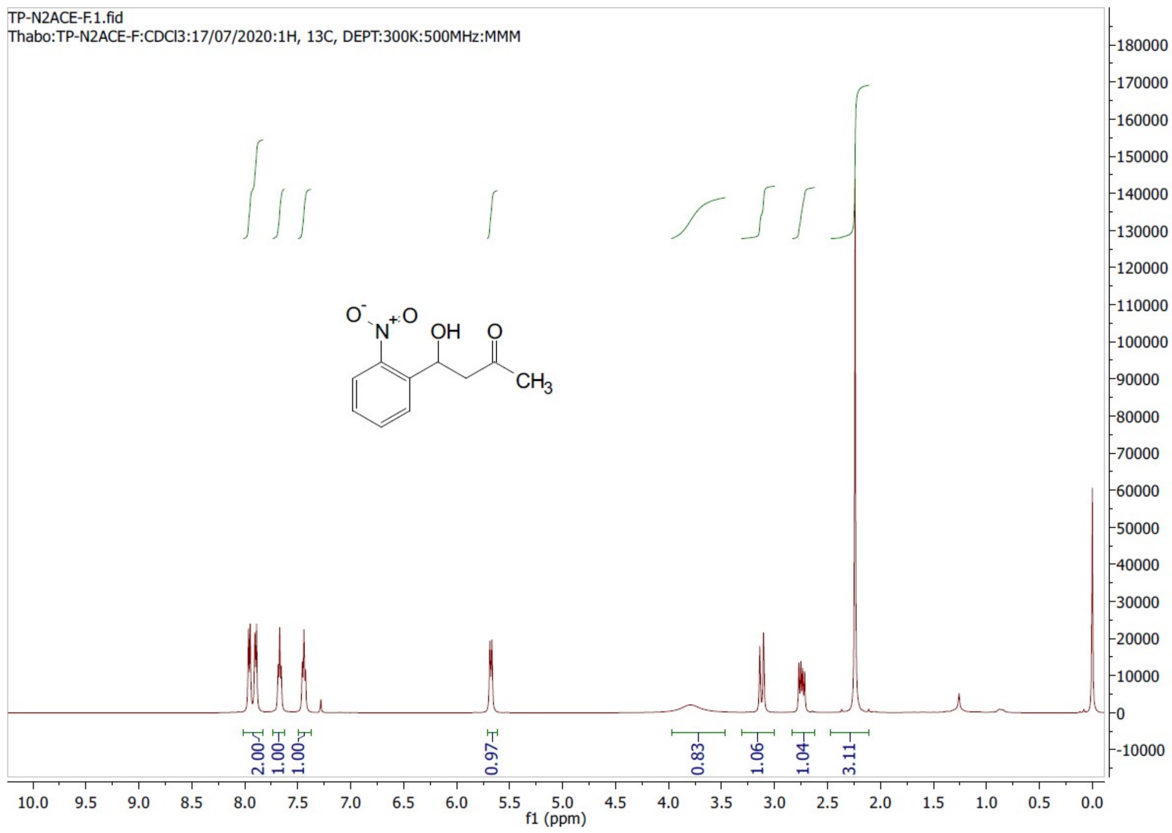

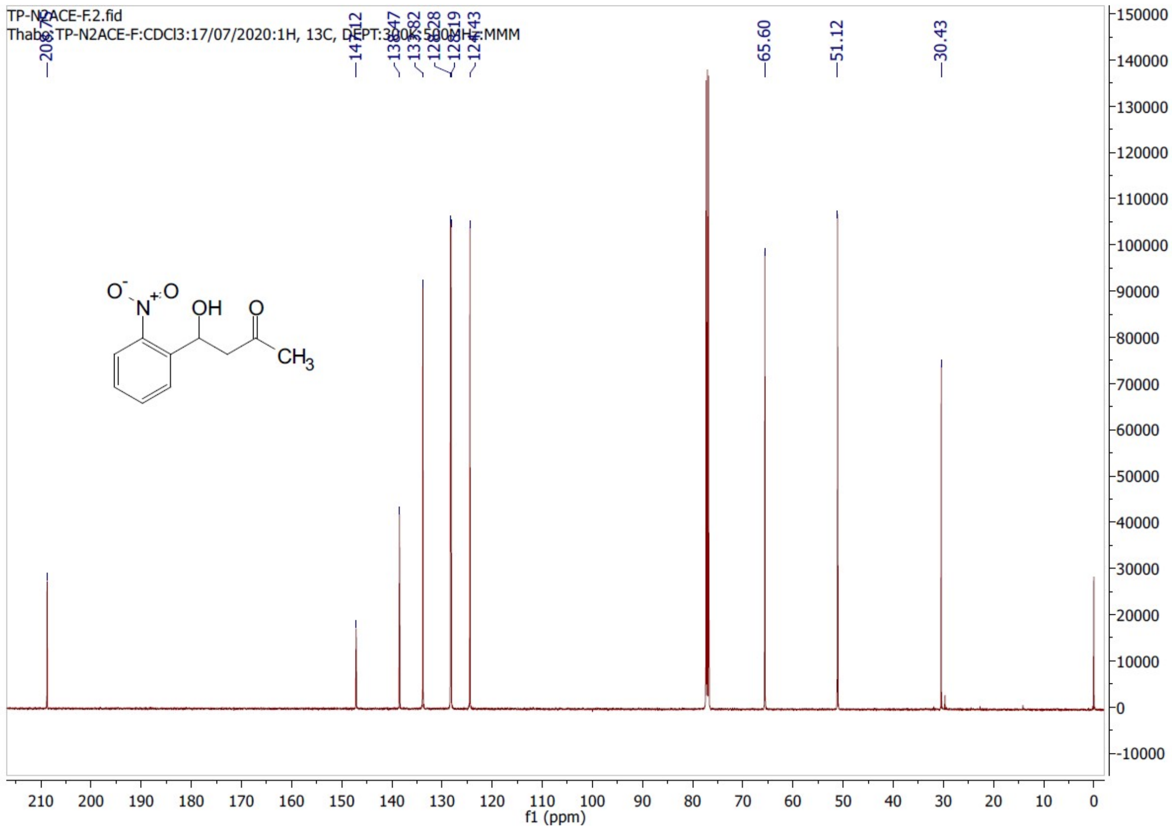

Figure S32: ^{1}H and ^{13}C NMR spectra of the aldol product **TP_A2N2ACE**

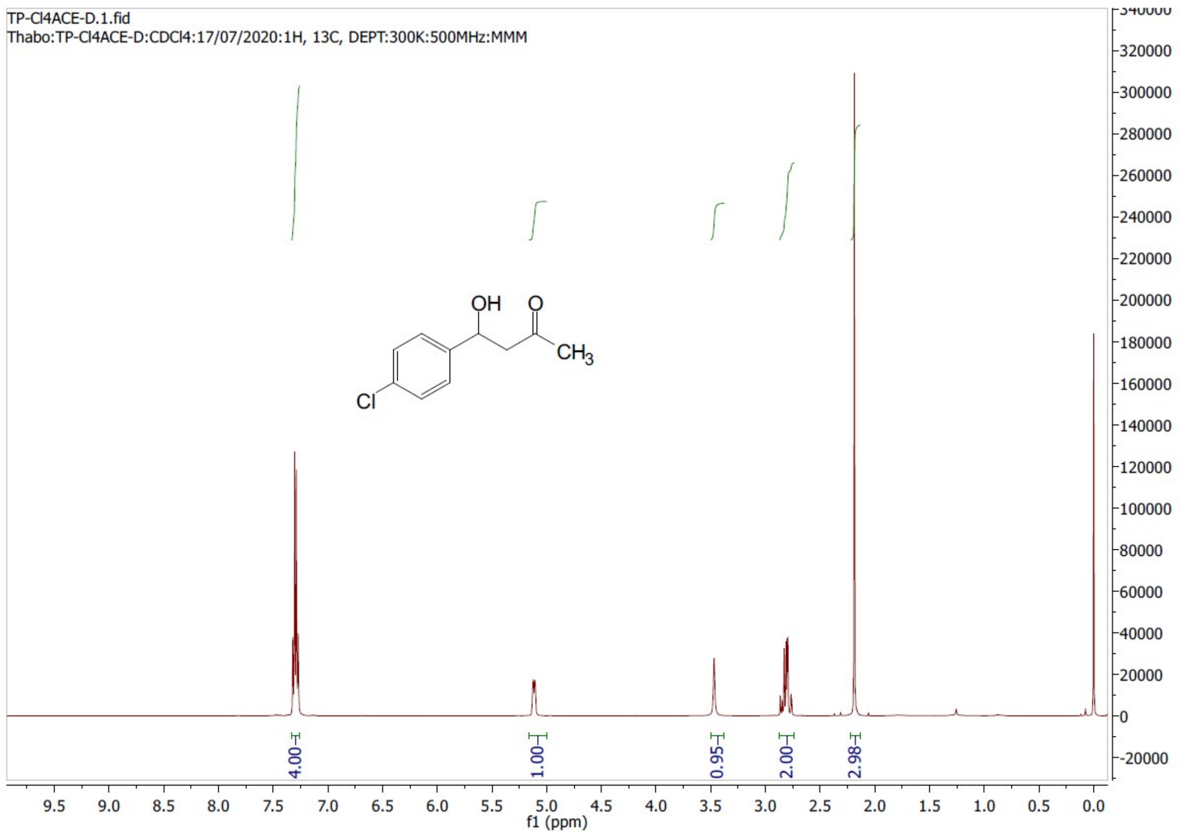

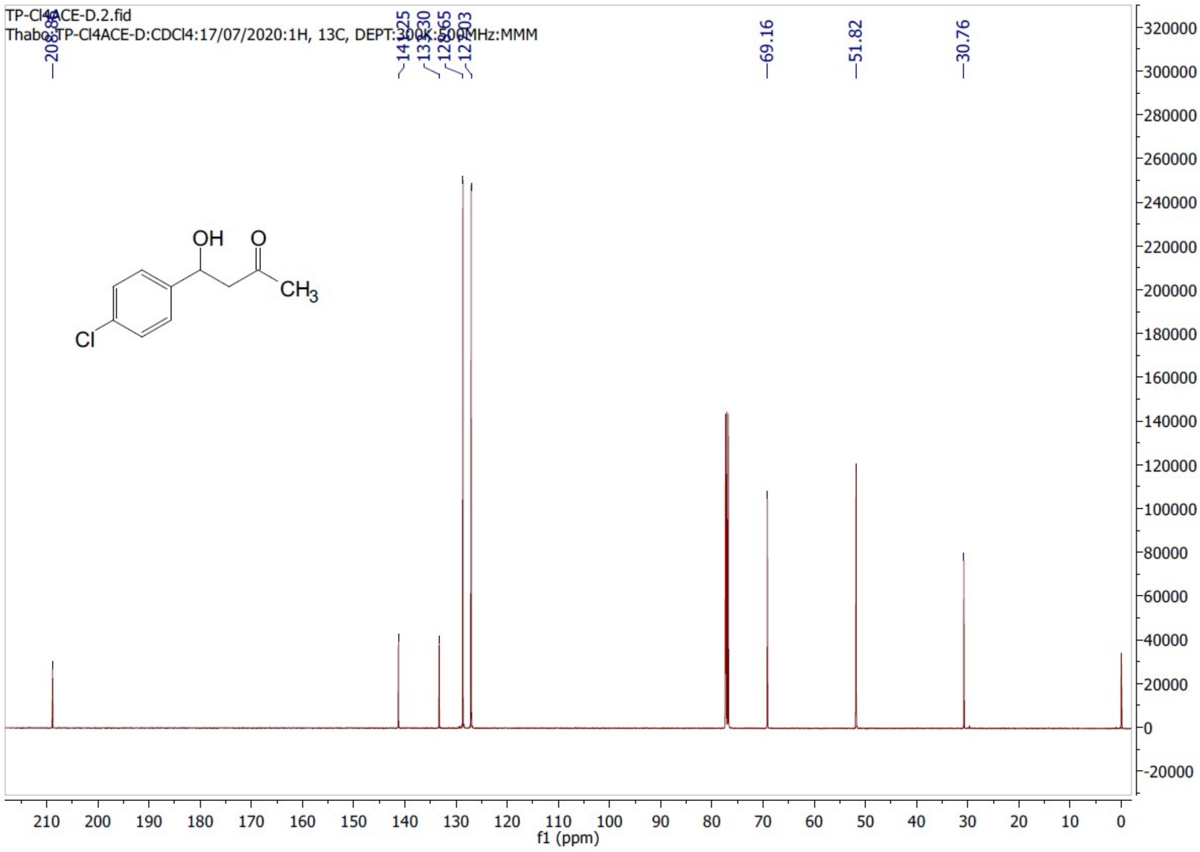

Figure S33: ^{1}H and ^{13}C NMR spectra of the aldol product **TP_A3Cl4ACE**

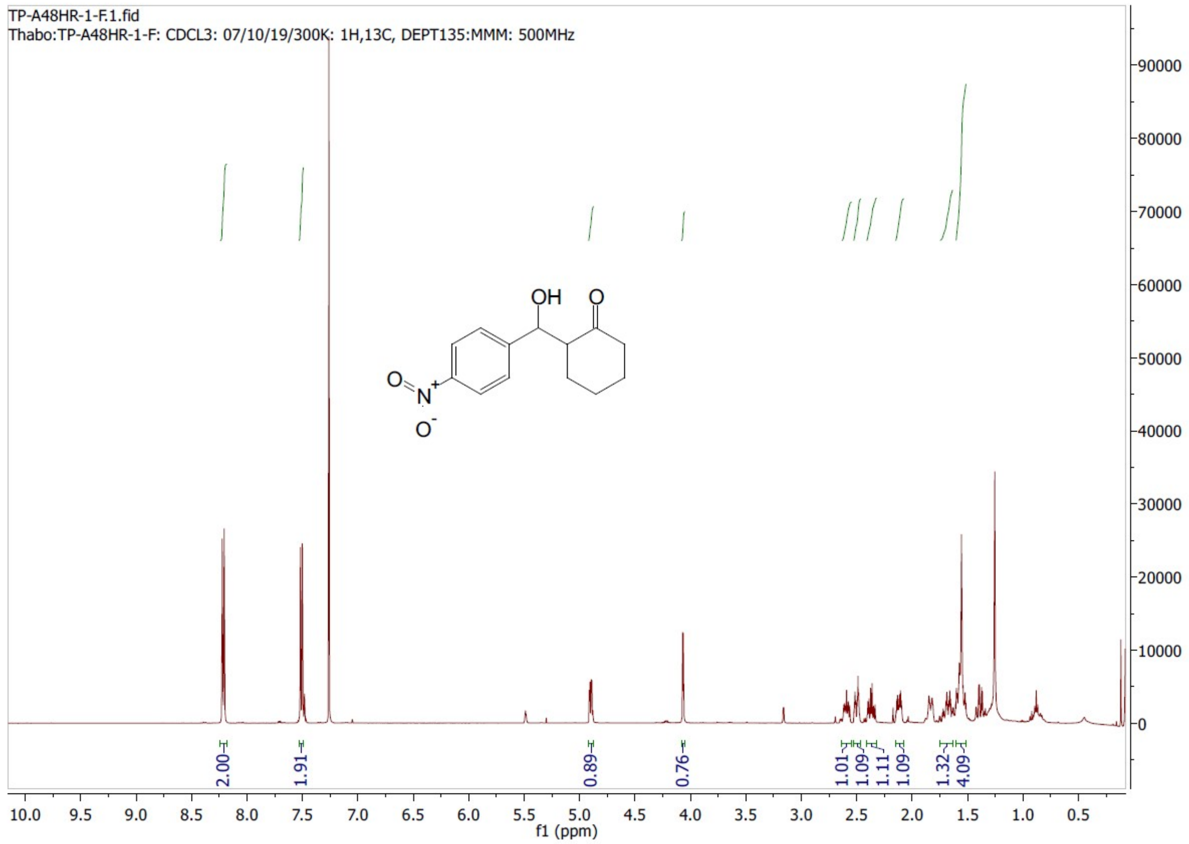

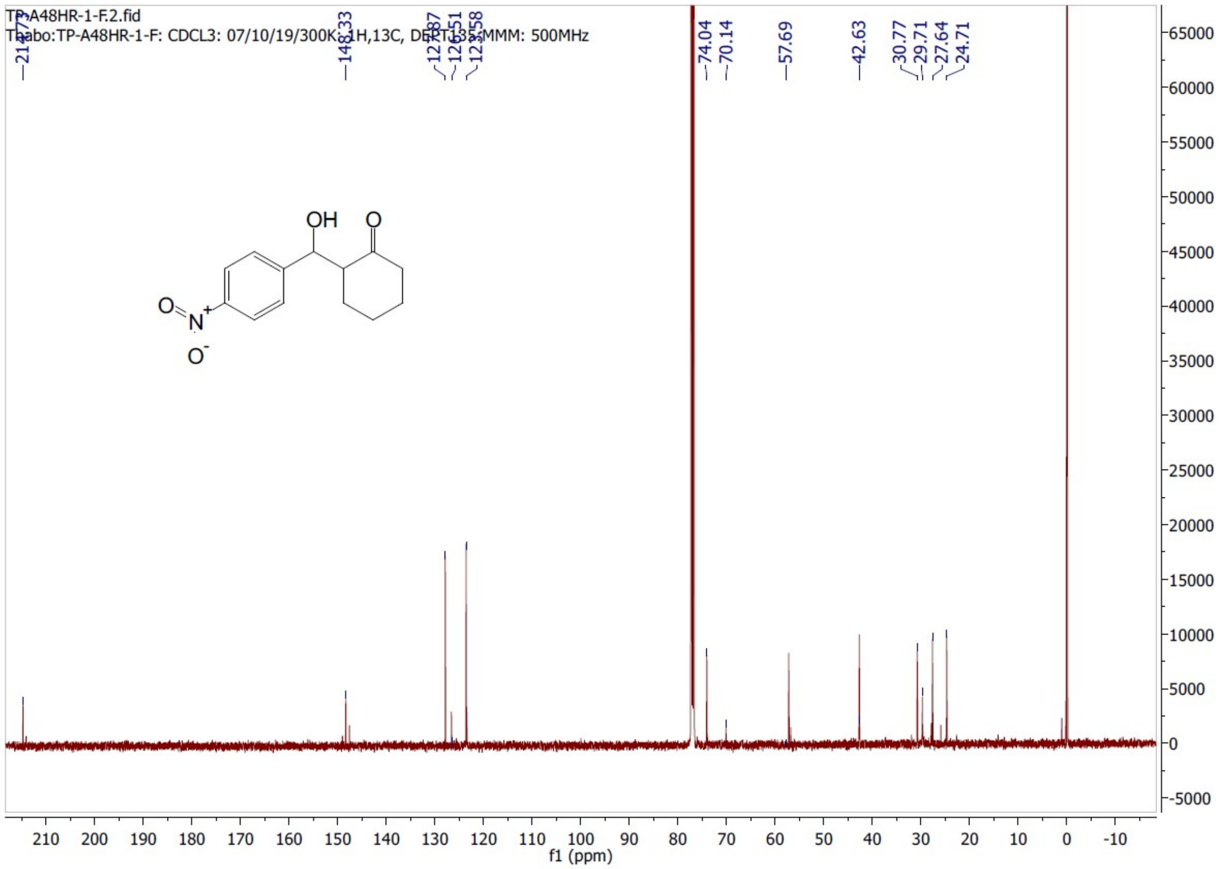

Figure S34: ^{1}H and ^{13}C NMR spectra of the aldol product **TP_A4N4CY**

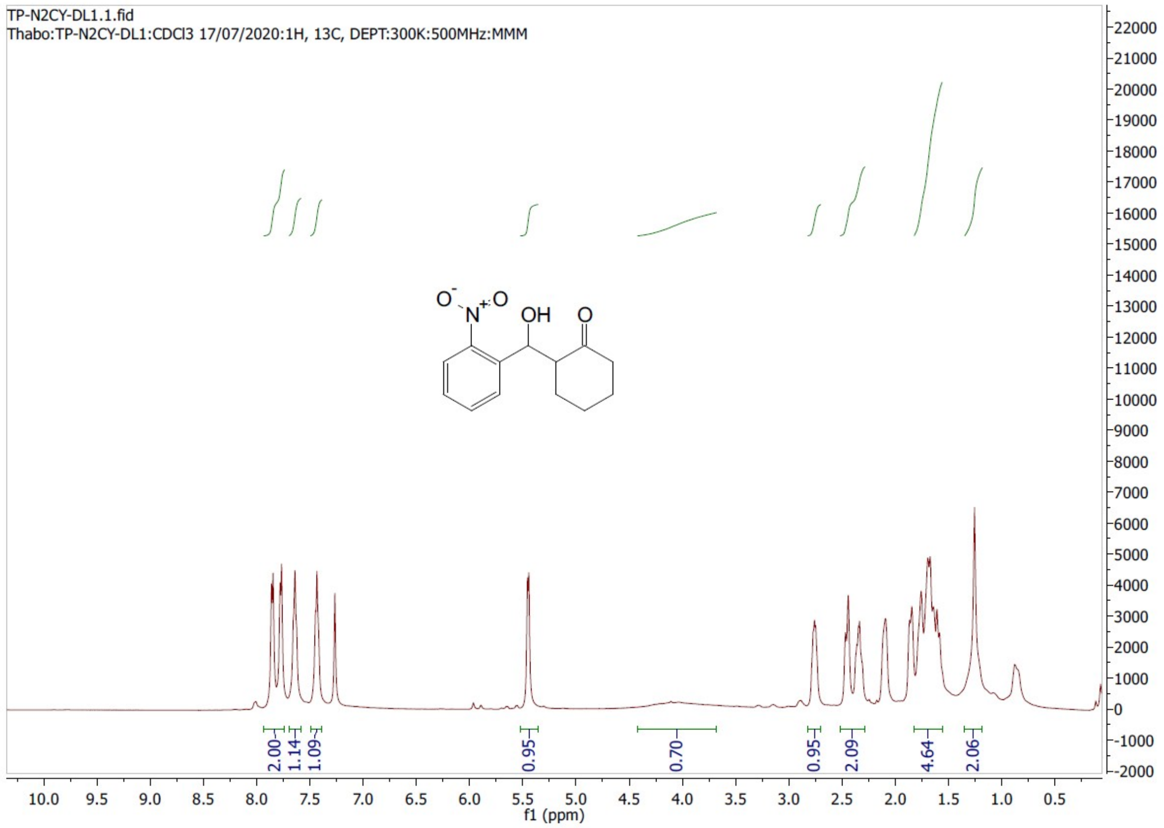

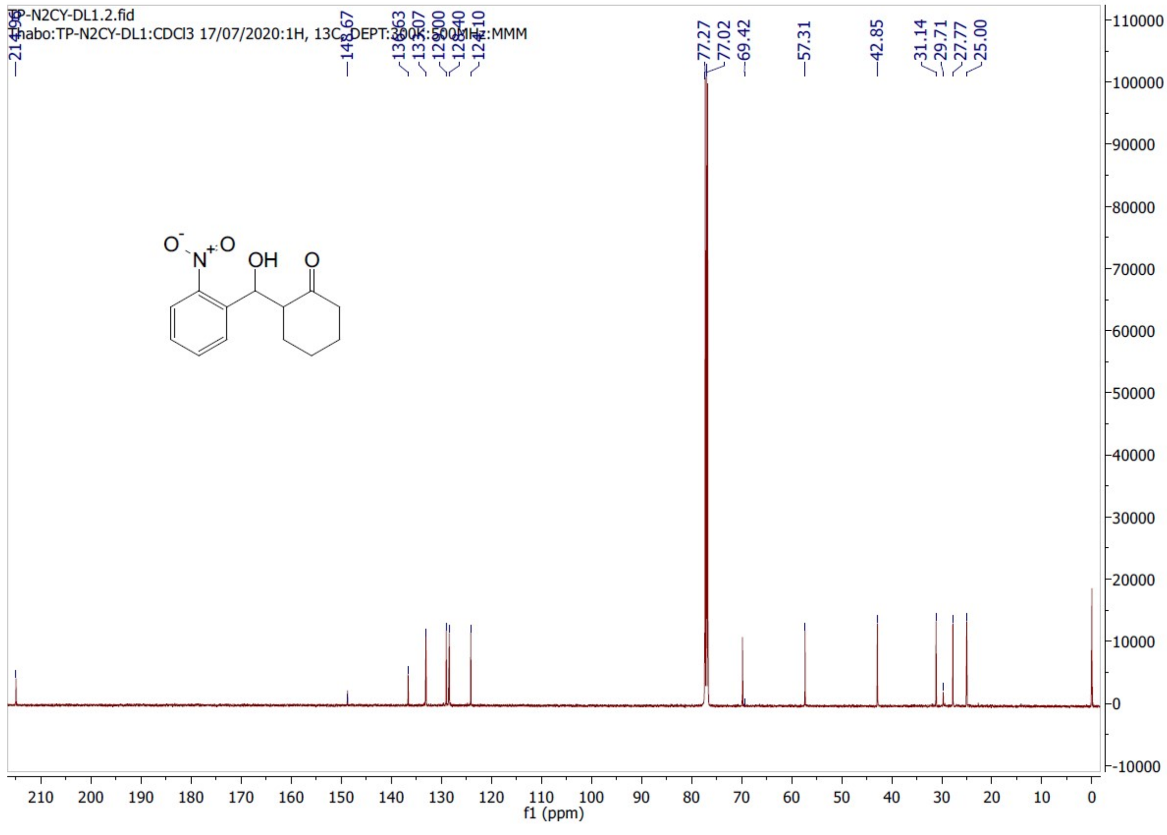

Figure S35: ^{1}H and ^{13}C NMR spectra of the aldol product **TP_A5N2CY**

Selected chiral HPLC chromatograms for aldol products

Using Acetone as a solvent and substrate **(Table 2)**

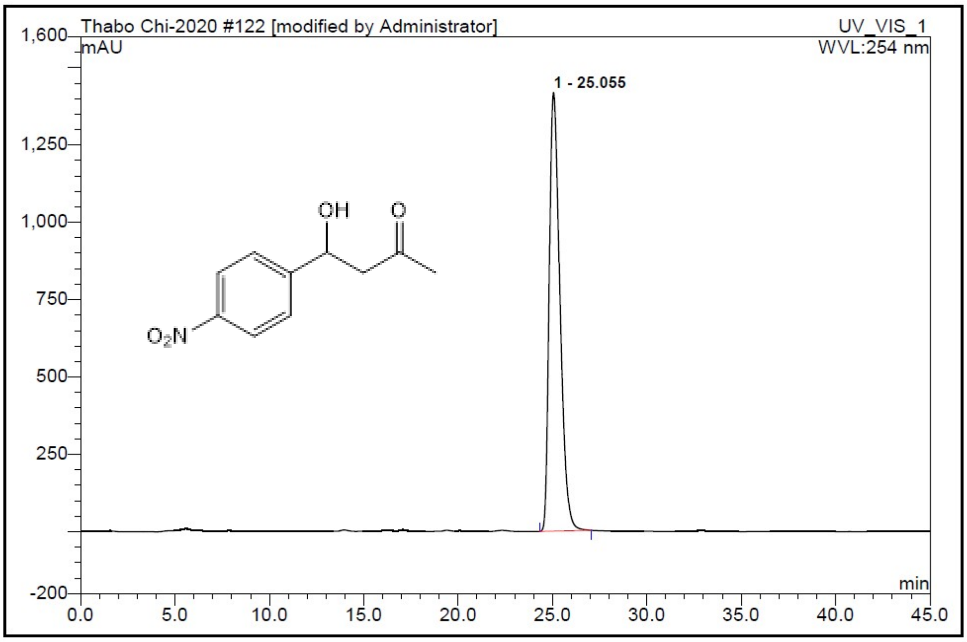

No.	Ret.Time min	Peak Name	Height mAU	Area mAU*min	Rel.Area %	Amount	Type
1	25.06	n.a.	1417.902	931.038	100.00	n.a.	BMB*
Total:			1417.902	931.038	100.00	0.000	

Figure SR: Chiral HPLC chromatogram of **TP_A1N4ACE** –**standard**

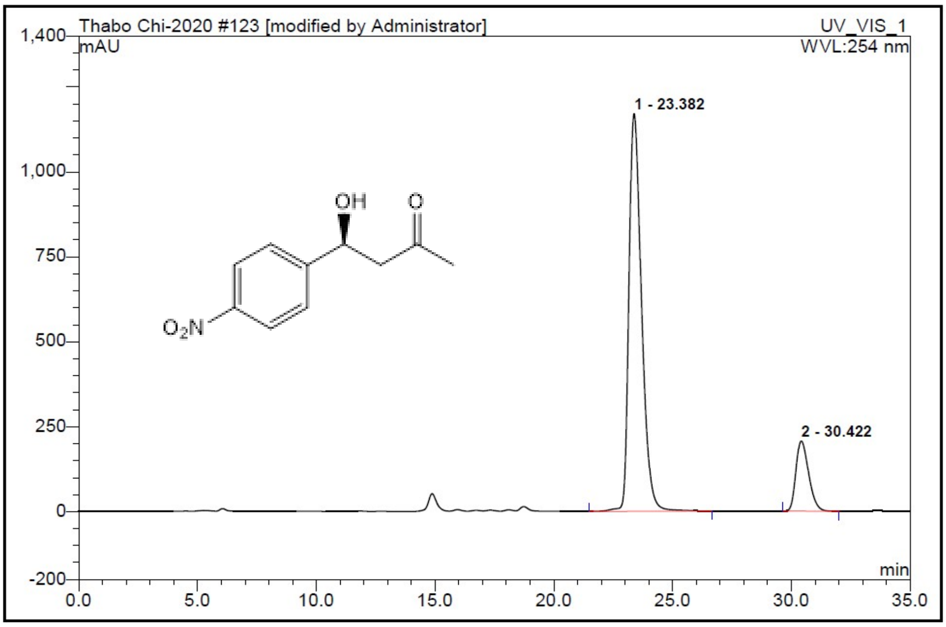

No.	Ret.Time min	Peak Name	Height mAU	Area mAU*min	Rel.Area %	Amount	Type
1	23.38	n.a.	1170.390	720.822	84.85	n.a.	BMB*
2	30.42	n.a.	206.183	128.699	15.15	n.a.	BMB
Total:			1376.573	849.521	100.00	0.000	

Figure S36: Chiral HPLC chromatogram of **TP_A1.1N4ACE** (entry1)

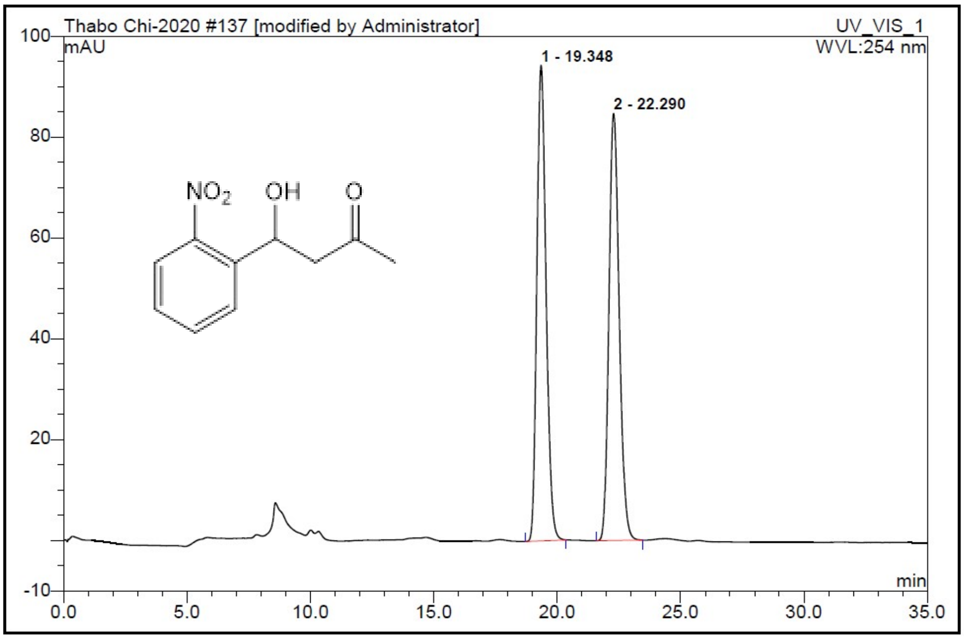

No.	Ret.Time min	Peak Name	Height mAU	Area mAU*min	Rel.Area %	Amount	Type
1	19.35	n.a.	94.329	40.831	49.99	n.a.	BMB*
2	22.29	n.a.	84.722	40.849	50.01	n.a.	BMB*
Total:			179.051	81.680	100.00	0.000	

Figure SR: Chiral HPLC chromatogram of **racemate TP_A2N2ACE-S2**

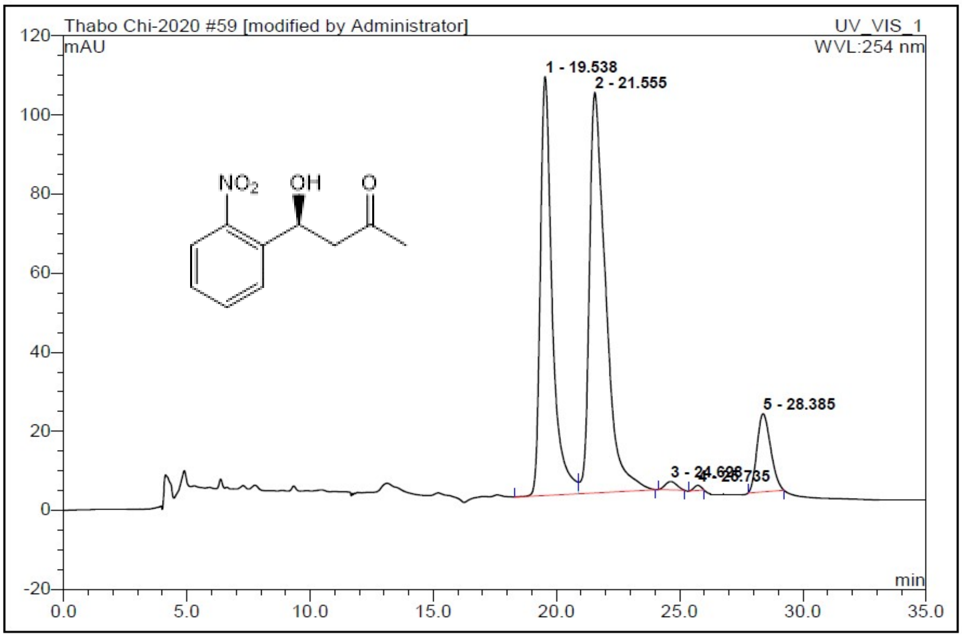

No.	Ret.Time min	Peak Name	Height mAU	Area mAU*min	Rel.Area %	Amount	Type
1	19.54	n.a.	105.908	59.370	39.25	n.a.	BM *
2	21.56	n.a.	101.309	77.648	51.34	n.a.	MB*
3	24.63	n.a.	2.125	1.156	0.76	n.a.	BMB*
4	25.74	n.a.	1.280	0.417	0.28	n.a.	BMB*
5	28.39	n.a.	19.739	12.653	8.37	n.a.	BMB*
Total:			230.360	151.244	100.00	0.000	

Figure S37: Chiral HPLC chromatogram of **TP_A2.1N2ACE** (entry 2)

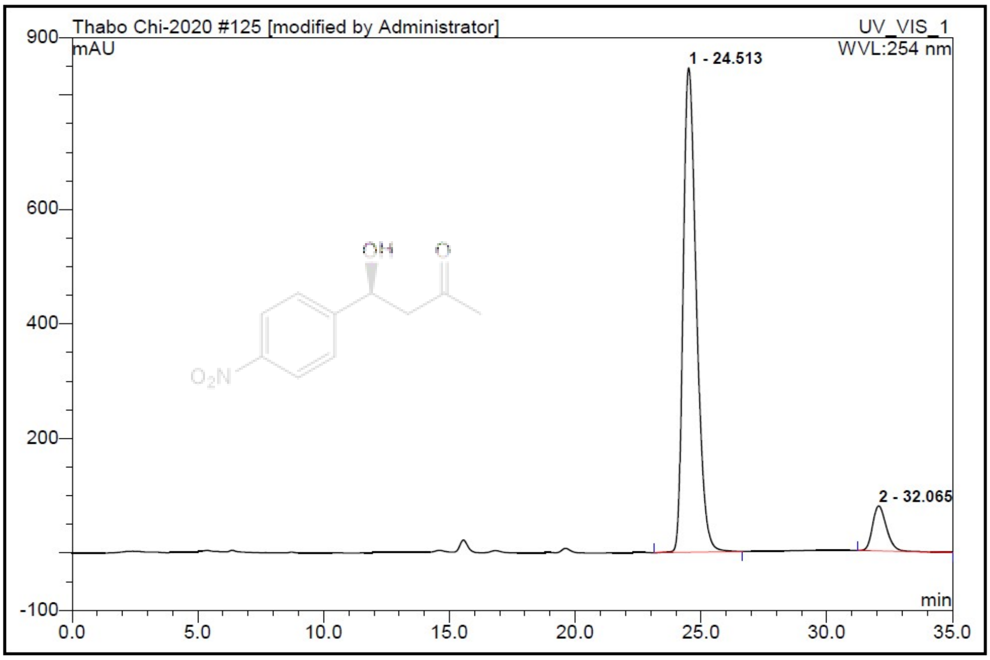

No.	Ret.Time min	Peak Name	Height mAU	Area mAU*min	Rel.Area %	Amount	Type
1	24.51	n.a.	846.014	515.043	91.14	n.a.	BMB*
2	32.07	n.a.	78.442	50.053	8.86	n.a.	BMB*
Total:			924.456	565.095	100.00	0.000	

Figure SR: Chiral HPLC chromatogram of **TP_A1.2N4ACE** (entry 4)

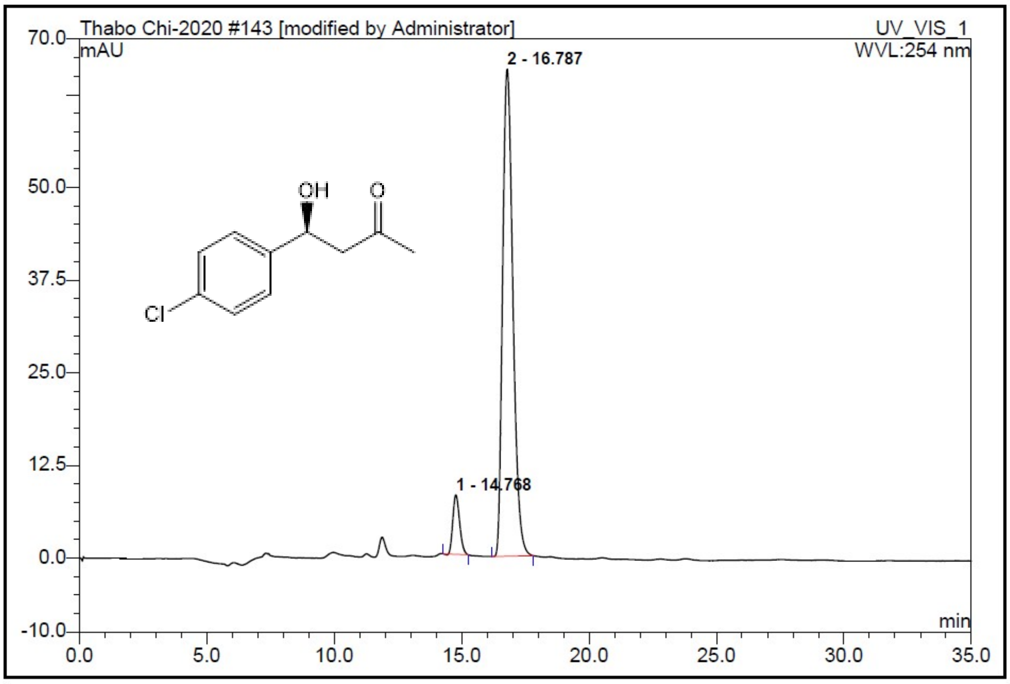

No.	Ret.Time min	Peak Name	Height mAU	Area mAU*min	Rel.Area %	Amount	Type
1	14.77	n.a.	8.036	2.462	7.43	n.a.	BMB*
2	16.79	n.a.	65.744	30.679	92.57	n.a.	BMB*
Total:			73.780	33.142	100.00	0.000	

Figure S38: Chiral HPLC chromatogram of **TP_A3Cl4ACE** (entry 6)

Using 8 mol% for reaction between Acetone and *p*-nitrobenzaldehyde **(Table 5)**

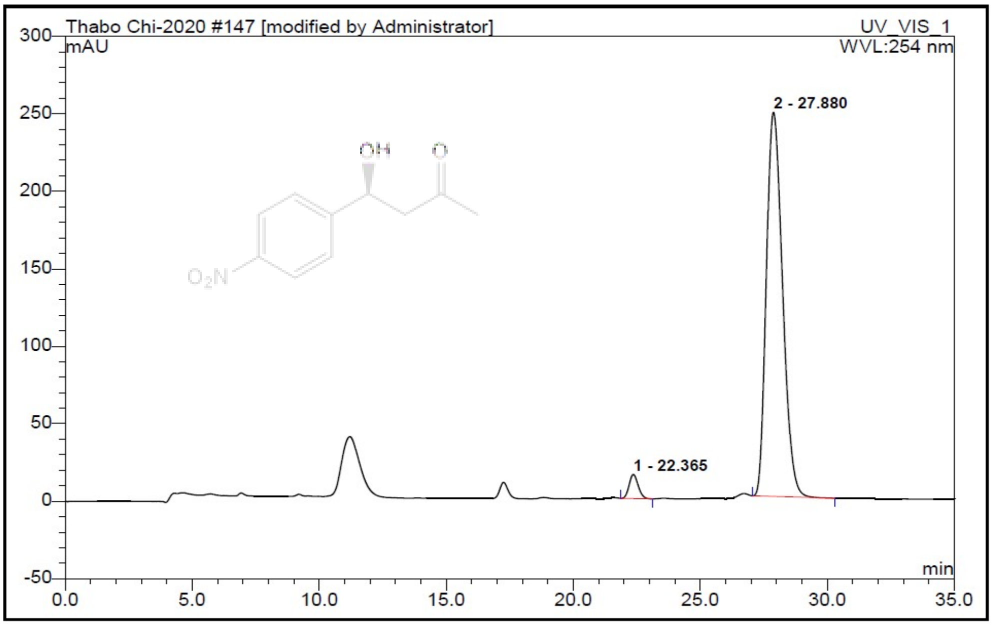

No.	Ret.Time min	Peak Name	Height mAU	Area mAU*min	Rel.Area %	Amount	Type
1	22.37	n.a.	15.513	6.386	3.37	n.a.	BMB*
2	27.88	n.a.	247.796	182.974	96.63	n.a.	BMB*
Total:			263.309	189.360	100.00	0.000	

Figure SR: Chiral HPLC chromatogram of **TP_A1.3N4ACE** (entry 11)

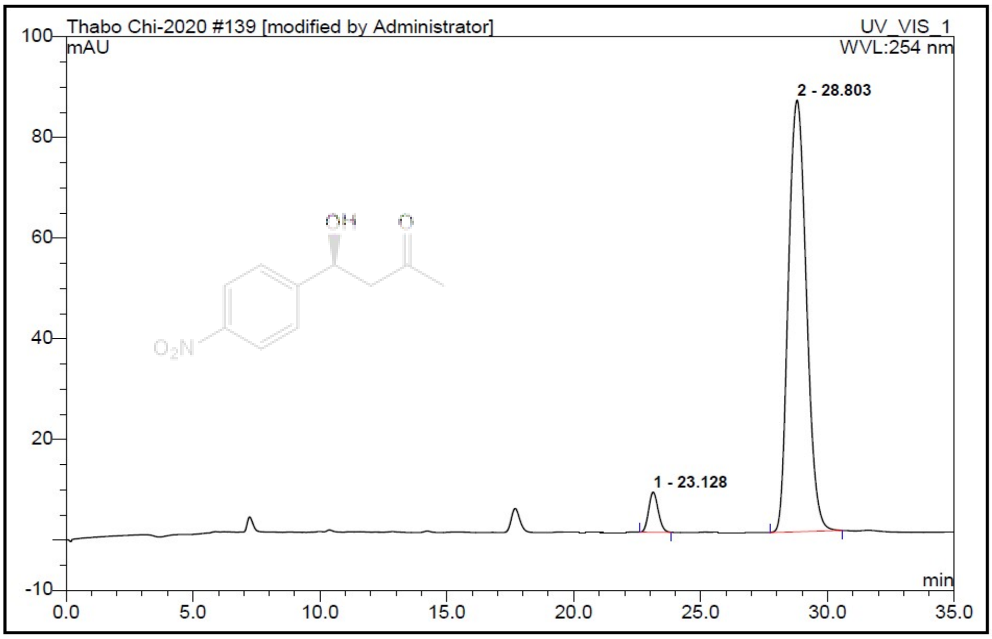

No.	Ret.Time min	Peak Name	Height mAU	Area mAU*min	Rel.Area %	Amount	Type
1	23.13	n.a.	7.962	3.480	4.71	n.a.	BMB*
2	28.80	n.a.	85.803	70.346	95.29	n.a.	BMB*
Total:			93.765	73.825	100.00	0.000	

Figure S.39: Chiral HPLC chromatogram of **TP-A1.4N4ACE** (entry 12)

Effect of substrate: Reaction between cyclohexanone and aromatic aldehydes **(Table 3)**

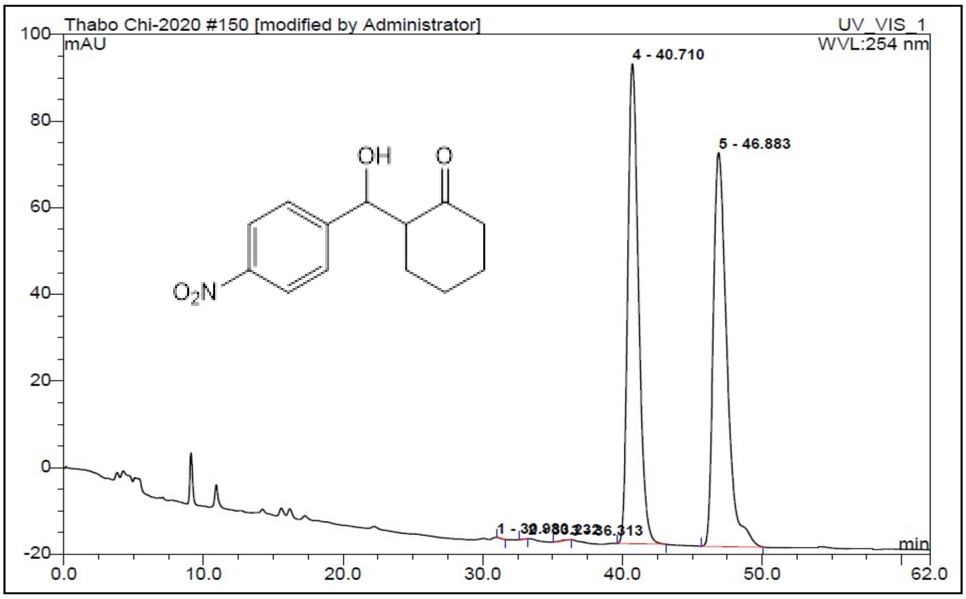

No.	Ret.Time min	Peak Name	Height mAU	Area mAU*min	Rel.Area %	Amount	Type
1	30.98	n.a.	0.011	0.002	0.00	n.a.	BMB*
2	33.23	n.a.	0.010	0.011	0.01	n.a.	BMB*
3	36.31	n.a.	0.008	0.046	0.02	n.a.	BMB*
4	40.71	n.a.	110.867	99.050	49.14	n.a.	BMB*
5	46.88	n.a.	90.970	102.454	50.83	n.a.	BMB
Total:			201.866	201.564	100.00	0.000	

Figure SR: Chiral HPLC chromatogram of **racemate TP_A4N4CY-R1**

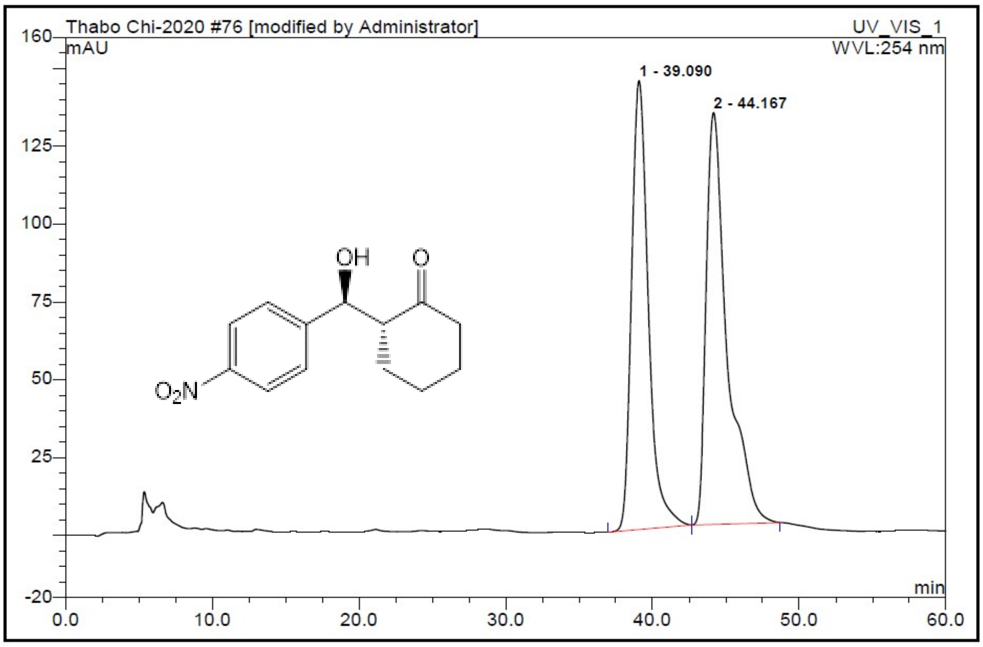

No.	Ret.Time min	Peak Name	Height mAU	Area mAU*min	Rel.Area %	Amount	Type
1	39.09	n.a.	144.164	191.099	46.64	n.a.	BMB*
2	44.17	n.a.	132.215	218.650	53.36	n.a.	BMB*
Total:			276.380	409.750	100.00	0.000	

Figure S40: Chiral HPLC chromatogram of **TP_A4.1N4CY** (entry 4)

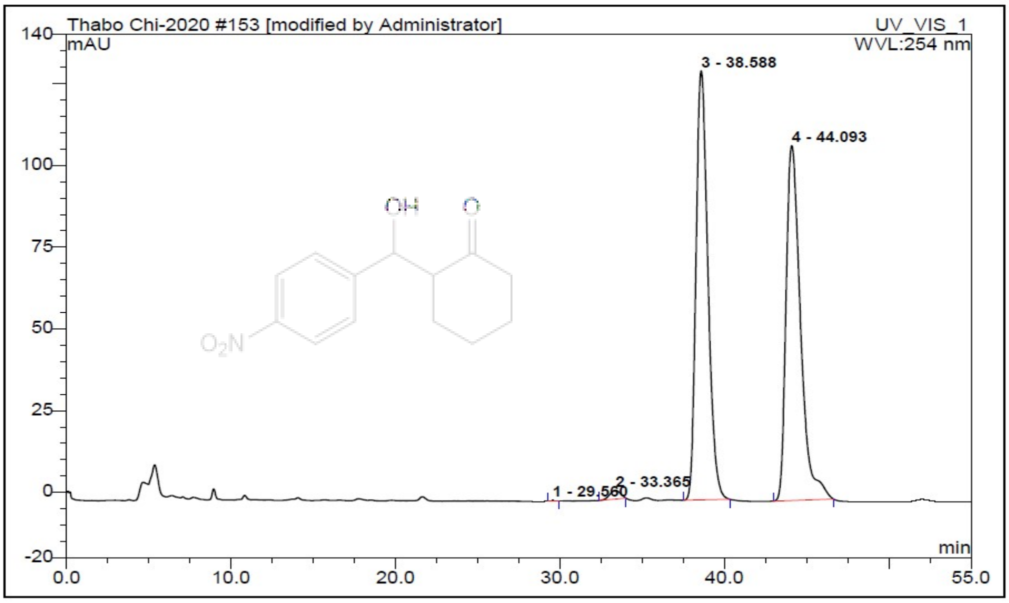

No.	Ret.Time min	Peak Name	Height mAU	Area mAU*min	Rel.Area %	Amount	Type
1	29.56	n.a.	0.181	0.069	0.03	n.a.	BMB*
2	33.37	n.a.	2.682	1.999	0.90	n.a.	BMB*
3	38.59	n.a.	131.271	109.853	49.38	n.a.	BMB*
4	44.09	n.a.	108.542	110.528	49.69	n.a.	BMB*
Total:			242.676	222.450	100.00	0.000	

Figure SR: Chiral HPLC chromatogram of **racemate TP_A4N4CY-R4**

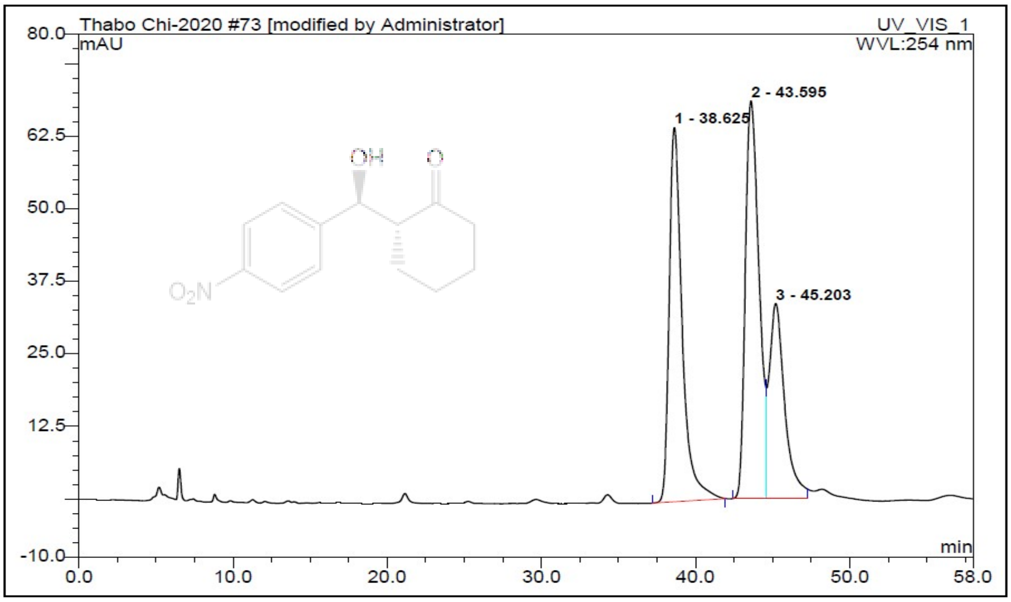

No.	Ret.Time min	Peak Name	Height mAU	Area mAU*min	Rel.Area %	Amount	Type
1	38.63	n.a.	64.448	62.770	36.38	n.a.	BMB*
2	43.60	n.a.	68.545	70.791	41.03	n.a.	BM *
3	45.20	n.a.	33.626	38.975	22.59	n.a.	M *
Total:			166.619	172.535	100.00	0.000	

Figure S41: Chiral HPLC chromatogram of **TP_A4.2N4CY** (entry 9)

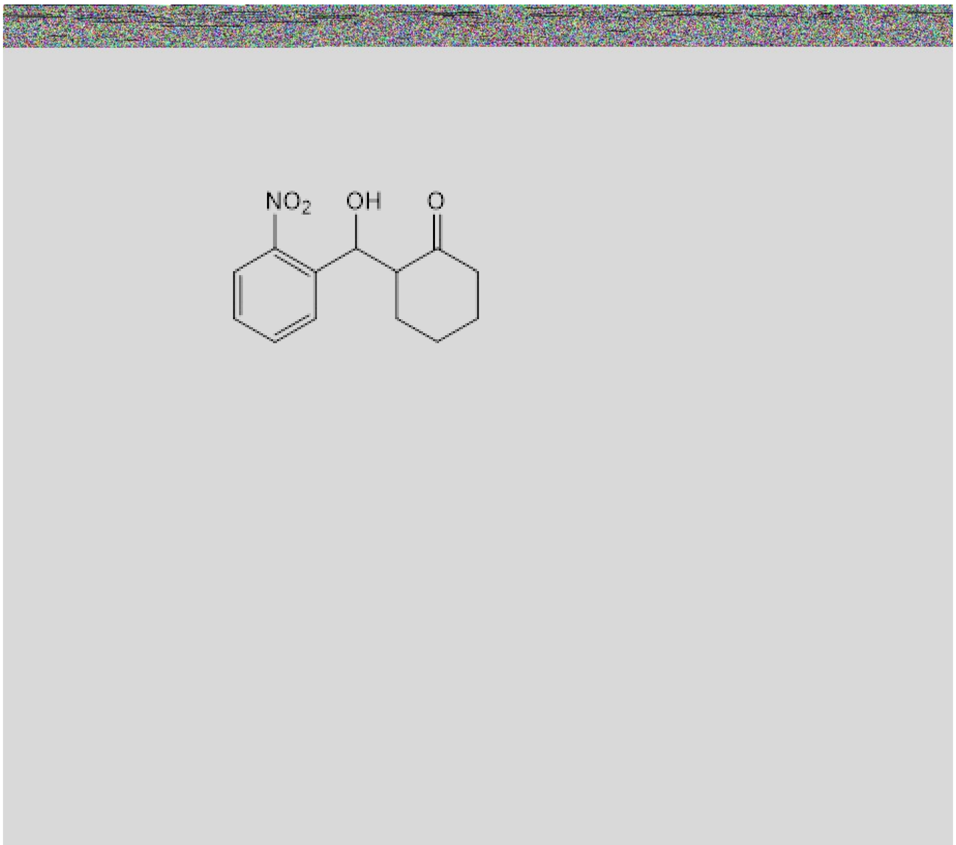

Figure SR: Chiral HPLC chromatogram of **racemate TP_A5N2CY-RA5**

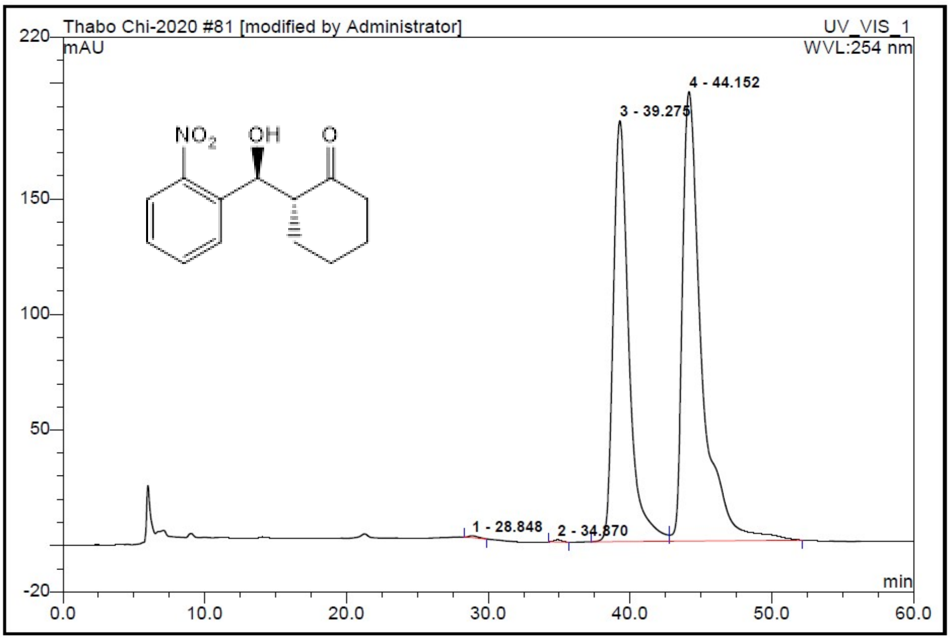

No.	Ret.Time min	Peak Name	Height mAU	Area mAU*min	Rel.Area %	Amount	Type
1	28.85	n.a.	0.923	0.717	0.13	n.a.	BMB
2	34.87	n.a.	0.962	0.630	0.12	n.a.	BMB*
3	39.28	n.a.	182.083	232.316	43.02	n.a.	BM *
4	44.15	n.a.	194.427	306.341	56.73	n.a.	MB*
Total:			378.395	540.004	100.00	0.000	

Figure S42: Chiral HPLC chromatogram of **TP_A5.1N2CY** (entry 10)

Effect of solvents -Reaction between cyclohexanone and *p*-nitrobenzaldehyde (**Table 4)**

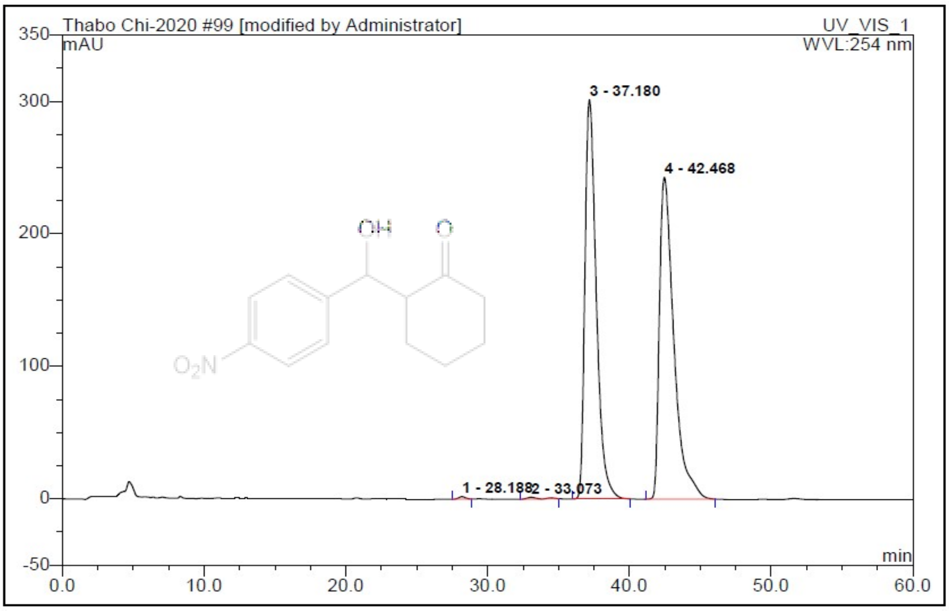

No.	Ret.Time min	Peak Name	Height mAU	Area mAU*min	Rel.Area %	Amount	Type
1	28.19	n.a.	1.891	1.030	0.18	n.a.	BMB*
2	33.07	n.a.	1.428	1.464	0.26	n.a.	BMB*
3	37.18	n.a.	301.322	280.012	49.20	n.a.	BMB*
4	42.47	n.a.	242.824	286.638	50.36	n.a.	BMB*
Total:			547.464	569.144	100.00	0.000	

Figure SR: Chiral HPLC chromatogram of **racemate TP_A4N4CY-R5**

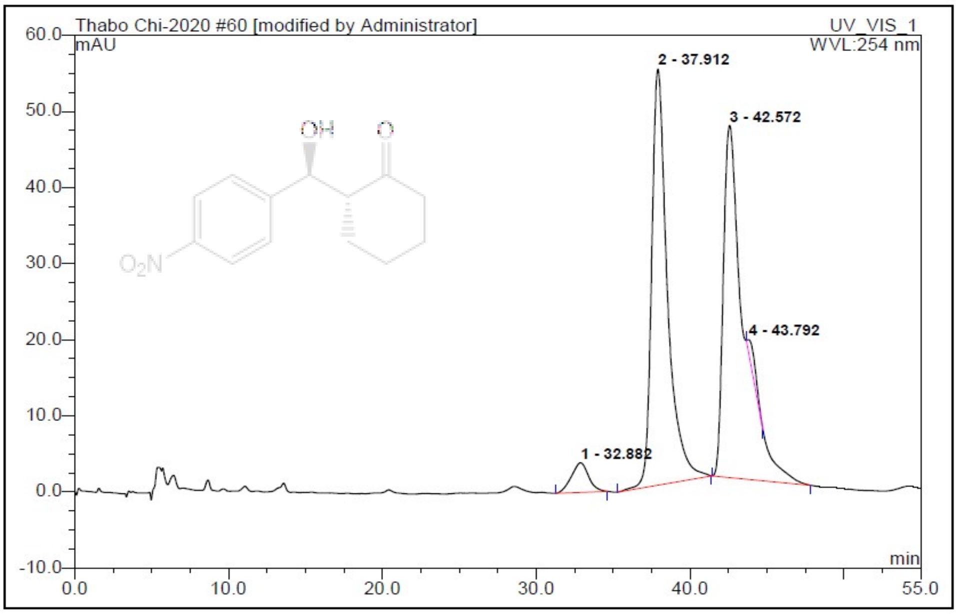

No.	Ret.Time min	Peak Name	Height mAU	Area mAU*min	Rel.Area %	Amount	Type
1	32.88	n.a.	3.885	5.043	3.48	n.a.	BMB*
2	37.91	n.a.	54.689	65.290	45.11	n.a.	BMB*
3	42.57	n.a.	46.283	72.571	50.14	n.a.	BMB*
4	43.79	n.a.	1.556	1.823	1.26	n.a.	Rd
Total:			106.413	144.727	100.00	0.000	

Figure S43: Chiral HPLC chromatogram of **TP_A4.3N4CY** (entry 4)

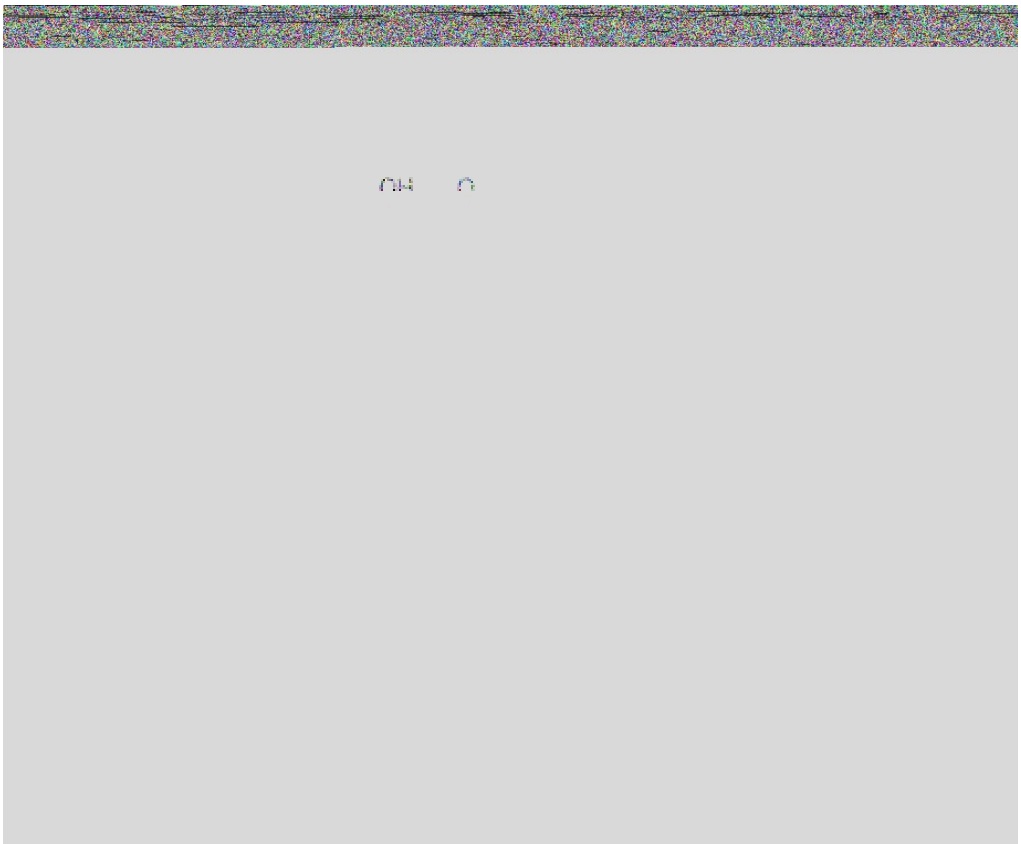

Figure SR: Chiral HPLC chromatogram of **racemate TP_A4N4CY-R6**

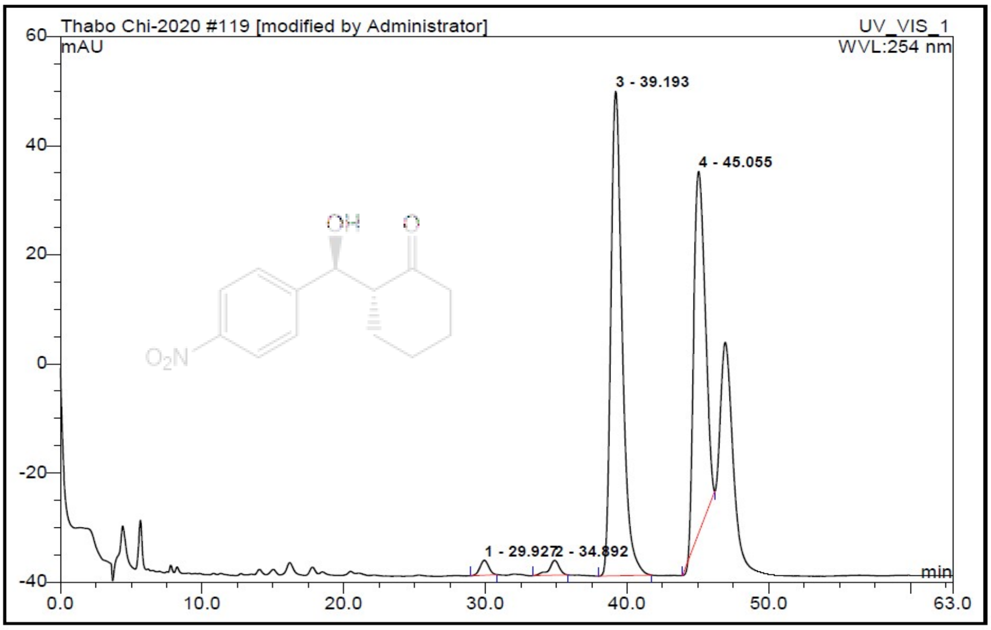

No.	Ret.Time min	Peak Name	Height mAU	Area mAU*min	Rel.Area %	Amount	Type
1	29.93	n.a.	2.726	1.960	1.32	n.a.	BMB*
2	34.89	n.a.	2.718	2.275	1.54	n.a.	BMB*
3	39.19	n.a.	88.765	82.427	55.70	n.a.	BMB*
4	45.06	n.a.	66.283	61.318	41.44	n.a.	BMB*
Total:			160.492	147.979	100.00	0.000	

Figure S44: Chiral HPLC chromatogram of **TP_A4.4N4CY** (entry 6)

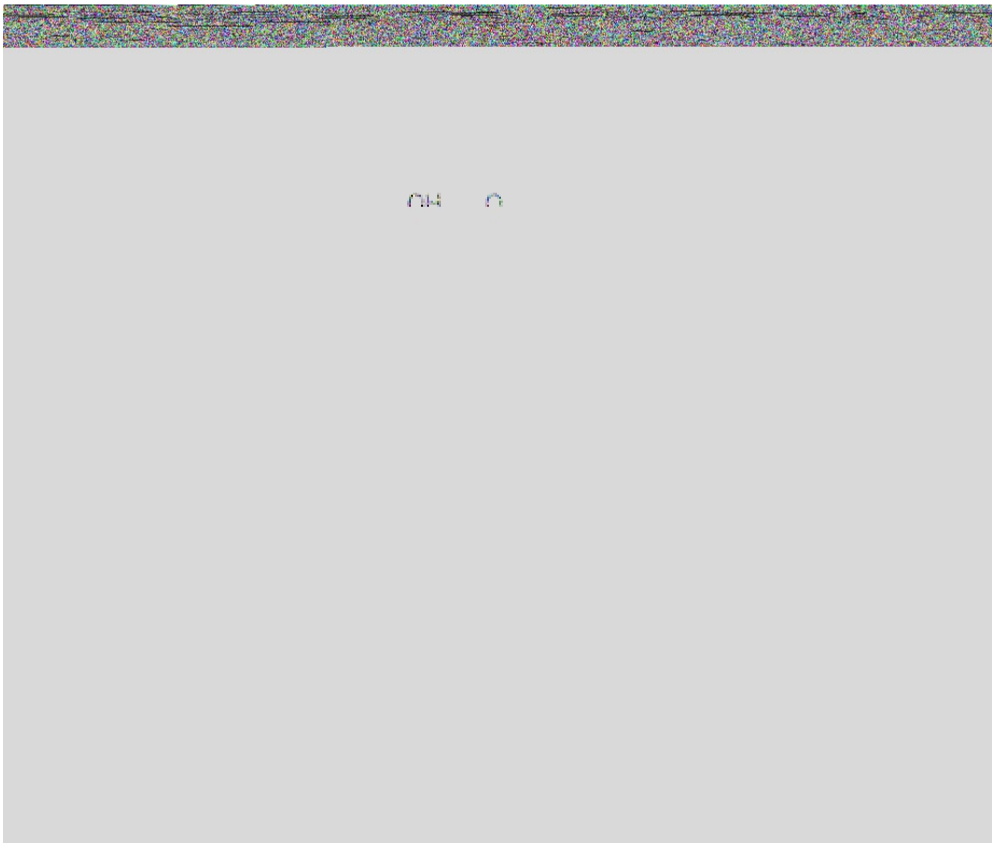

Figure SR: Chiral HPLC chromatogram of **racemate TP_A4N4CY-R7**

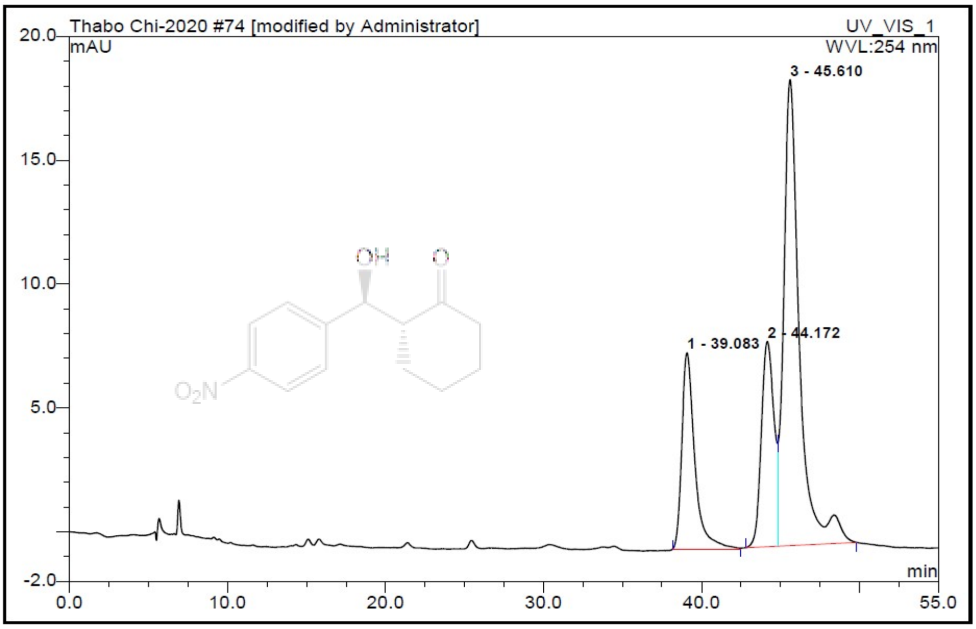

No.	Ret.Time min	Peak Name	Height mAU	Area mAU*min	Rel.Area %	Amount	Type
1	39.08	n.a.	7.925	7.582	20.41	n.a.	BM *
2	44.17	n.a.	8.280	7.602	20.46	n.a.	BM *
3	45.61	n.a.	18.812	21.974	59.14	n.a.	MB*
**Total:**			35.017	37.158	100.00	0.000	

Figure S45: Chiral HPLC chromatogram of **TP_A4.5N4CY** (entry 9)

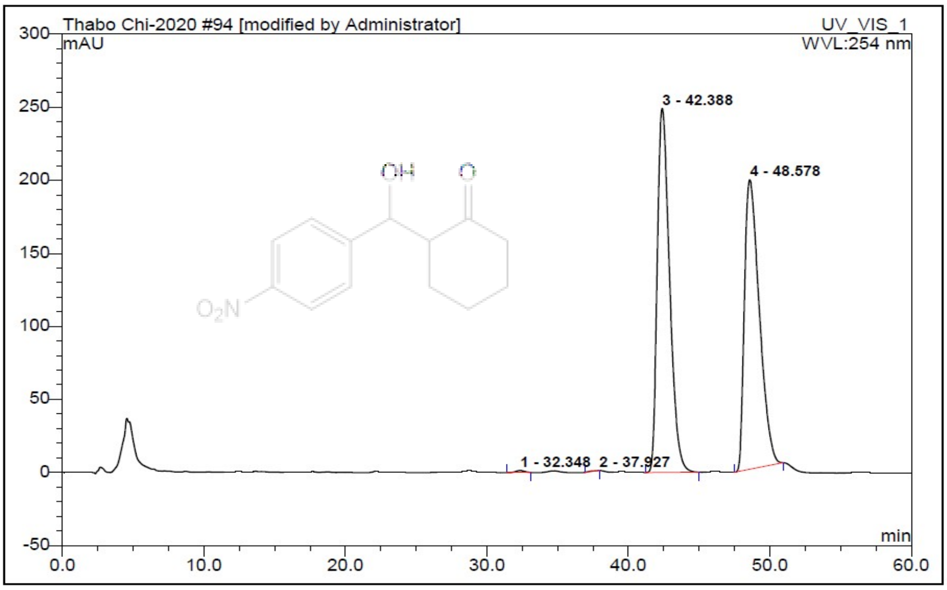

No.	Ret.Time min	Peak Name	Height mAU	Area mAU*min	Rel.Area %	Amount	Type
1	32.35	n.a.	1.387	0.998	0.20	n.a.	BMB*
2	37.93	n.a.	0.032	0.182	0.04	n.a.	BMB*
3	42.39	n.a.	249.159	251.603	50.91	n.a.	BMB*
4	48.58	n.a.	198.169	241.383	48.85	n.a.	BMB*
Total:			448.746	494.165	100.00	0.000	

Figure SR: Chiral HPLC chromatogram of **racemate TP_A4N4CY-R8**

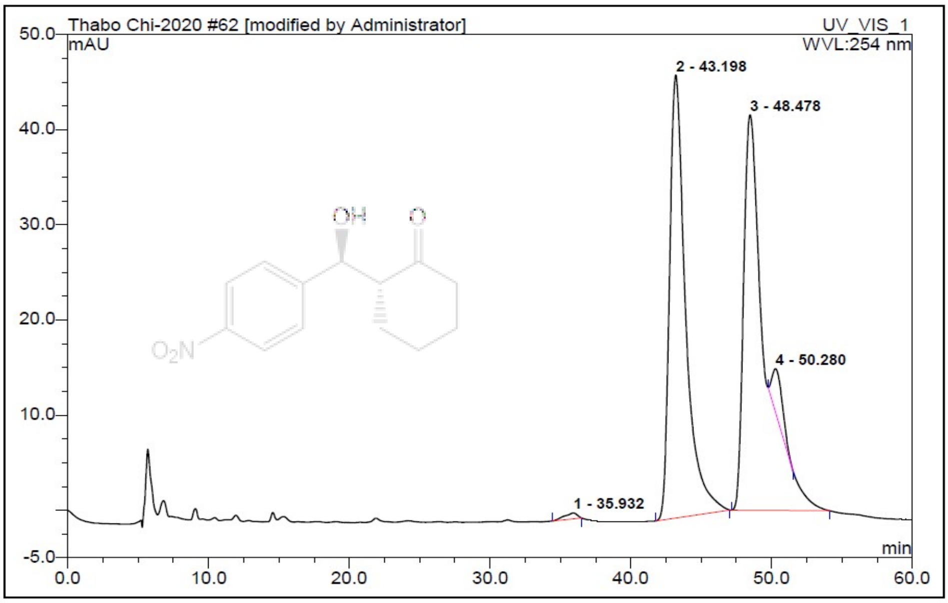

No.	Ret.Time min	Peak Name	Height mAU	Area mAU*min	Rel.Area %	Amount	Type
1	35.93	n.a.	0.625	0.676	0.48	n.a.	BMB*
2	43.20	n.a.	46.566	61.847	44.33	n.a.	BMB*
3	48.48	n.a.	41.579	72.831	52.20	n.a.	BMB*
4	50.28	n.a.	4.533	4.162	2.98	n.a.	Rd
Total:			93.304	139.516	100.00	0.000	

Figure S46: Chiral HPLC chromatogram of **TP_A4.6N4CY** (entry 13)

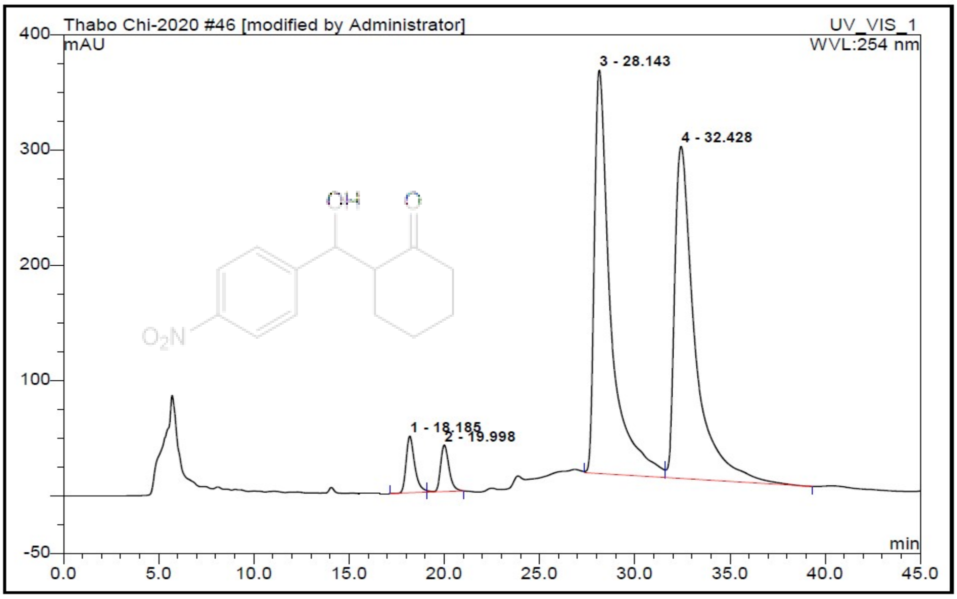

No.	Ret.Time min	Peak Name	Height mAU	Area mAU*min	Rel.Area %	Amount	Type
1	18.19	n.a.	49.255	25.866	3.44	n.a.	BM *
2	20.00	n.a.	40.366	20.758	2.76	n.a.	MB*
3	28.14	n.a.	350.081	350.136	46.53	n.a.	M *
4	32.43	n.a.	288.095	355.814	47.28	n.a.	MB*
Total:			727.798	752.574	100.00	0.000	

Figure SR: Chiral HPLC chromatogram of **racemate TP_A4N4CY-R10**

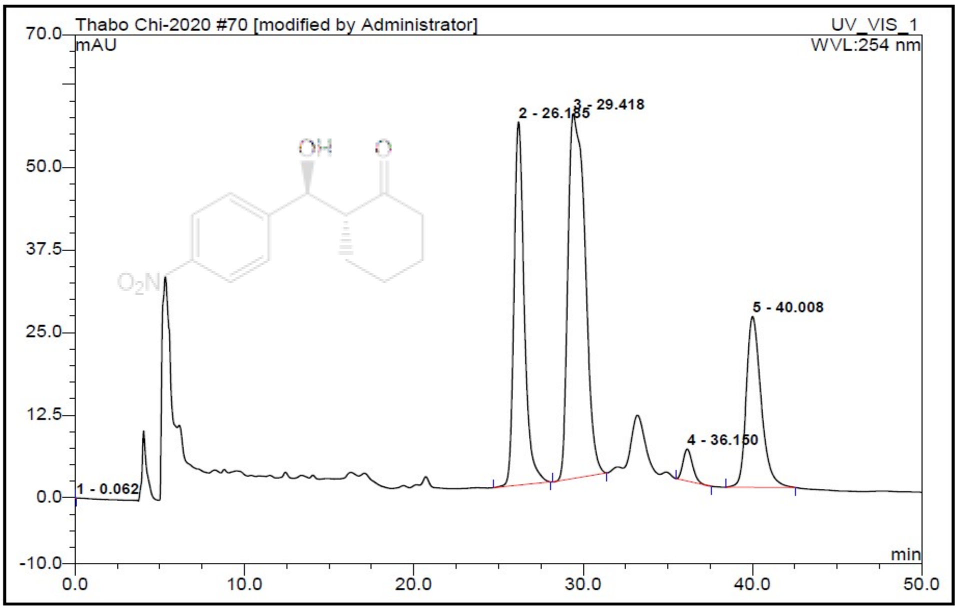

No.	Ret.Time min	Peak Name	Height mAU	Area mAU*min	Rel.Area %	Amount	Type
1	0.06	n.a.	0.005	0.000	0.00	n.a.	BMB
2	26.19	n.a.	54.929	38.662	29.15	n.a.	BMB*
3	29.42	n.a.	55.175	64.604	48.70	n.a.	BMB*
4	36.15	n.a.	4.826	3.142	2.37	n.a.	BMB*
5	40.01	n.a.	25.817	26.244	19.78	n.a.	BMB*
Total:			140.752	132.652	100.00	0.000	

Figure S47: Chiral HPLC chromatogram of **TP_A4.7N4CY** (entry 15)

Aldol products using 8 mol% between cyclohexanone and *p*-nitrobenzaldehyde (Table 5)

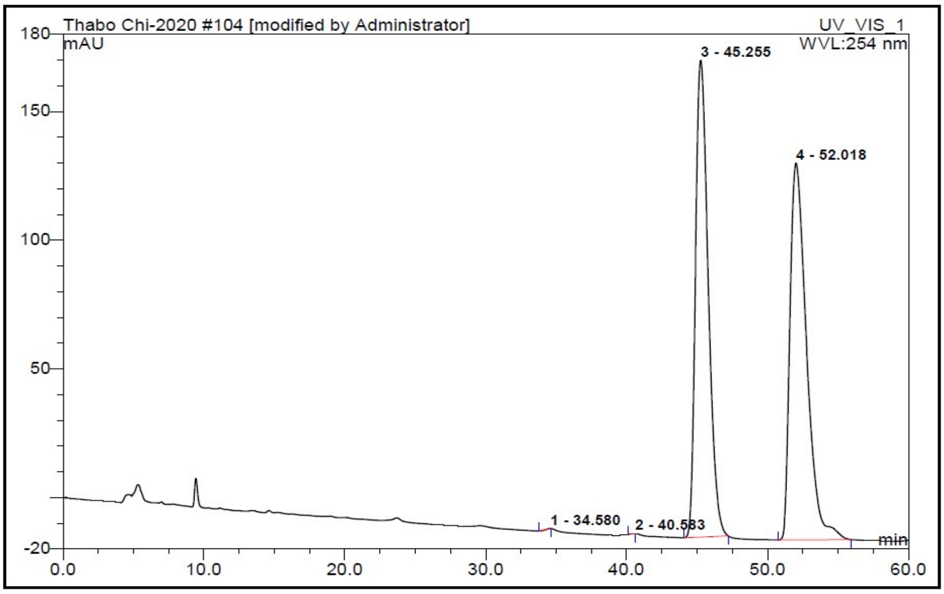

No.	Ret.Time min		Peak Name	Height mAU	Area mAU*min	Rel.Area %	Amount	Type
1	34.58	n.a.		0.066	0.017	0.00	n.a.	BMB*
2	40.58	n.a.		0.008	0.001	0.00	n.a.	BMB*
3	45.26	n.a.		185.230	193.103	49.21	n.a.	BMB*
4	52.02	n.a.		146.419	199.321	50.79	n.a.	BMB*
Total:				331.723	392.442	100.00	0.000	

Figure SR: Chiral HPLC chromatogram of **racemate TP_A4N4CY-R13**

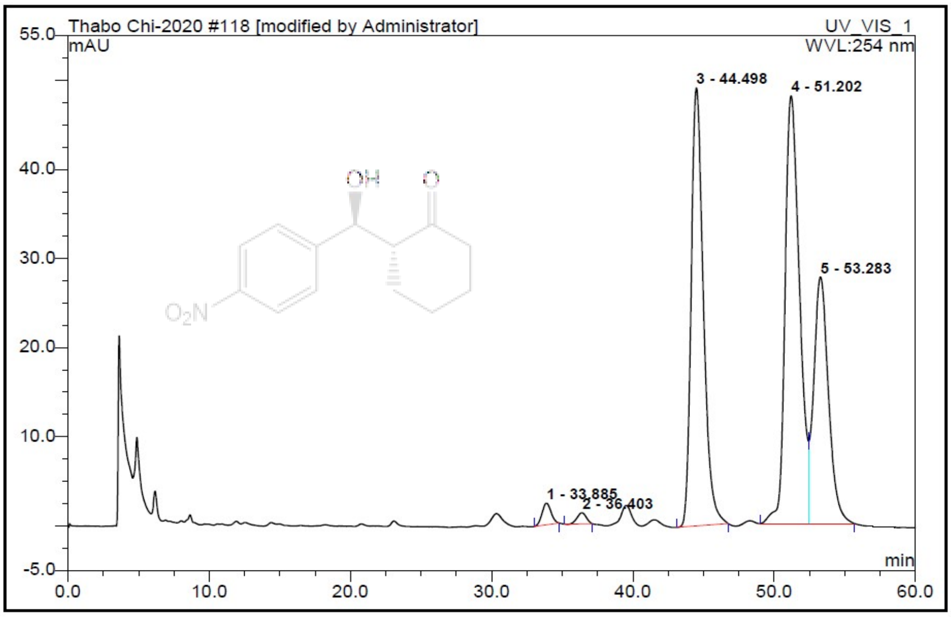

No.	Ret.Time min		Peak Name	Height mAU	Area mAU*min	Rel.Area %	Amount	Type
1	33.89	n.a.		2.422	1.802	1.26	n.a.	BMB*
2	36.40	n.a.		1.243	0.933	0.65	n.a.	BMB*
3	44.50	n.a.		49.146	49.059	34.41	n.a.	BMB*
4	51.20	n.a.		47.979	56.946	39.94	n.a.	BM
5	53.28	n.a.		27.664	33.849	23.74	n.a.	MB*
Total:				128.454	142.589	100.00	0.000	

Figure S48: Chiral HPLC chromatogram of **TP_A4.10N4CY** (entry 5)

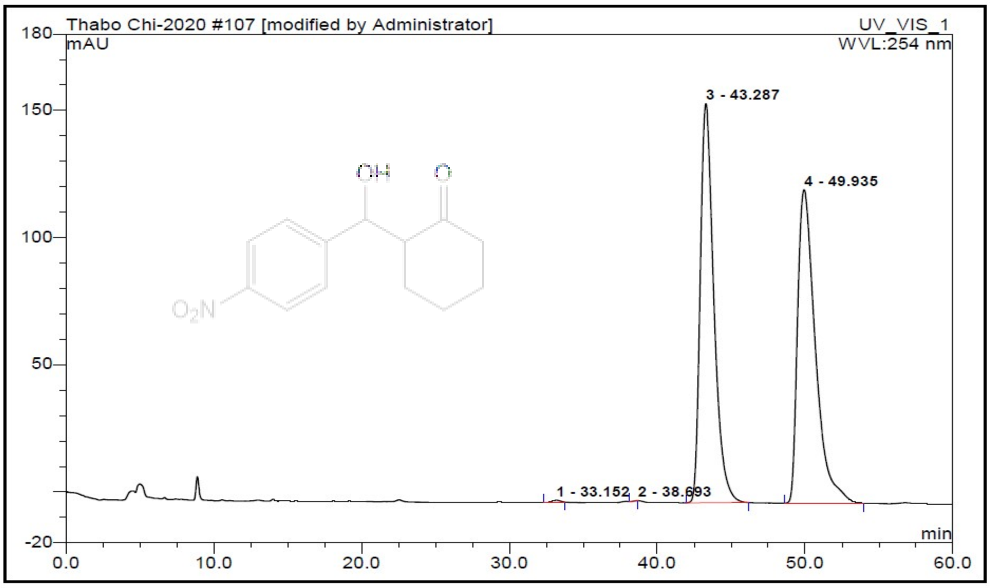

No.	Ret.Time min	Peak Name	Height mAU	Area mAU*min	Rel.Area %	Amount	Type
1	33.15	n.a.	0.855	0.598	0.18	n.a.	BM *
2	38.69	n.a.	0.010	0.003	0.00	n.a.	BMB*
3	43.29	n.a.	156.876	167.356	49.27	n.a.	BMB*
4	49.94	n.a.	123.206	171.693	50.55	n.a.	BMB
Total:			280.947	339.650	100.00	0.000	

Figure SR: Chiral HPLC chromatogram of **racemate TP_A4N4CY-R12**

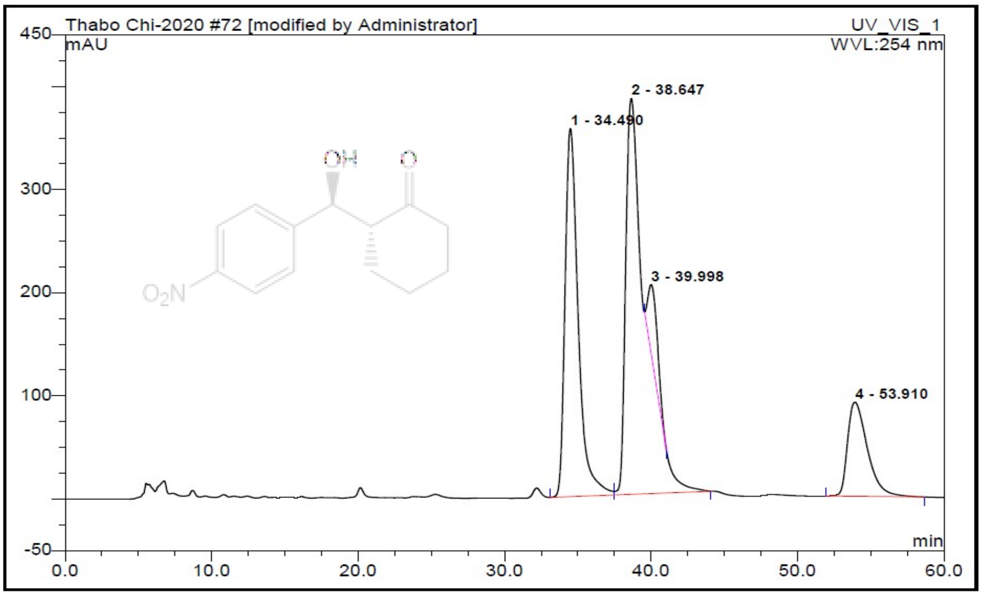

No.	Ret.Time min	Peak Name	Height mAU	Area mAU*min	Rel.Area %	Amount	Type
1	34.49	n.a.	356.697	372.865	31.78	n.a.	BM *
2	38.65	n.a.	383.506	603.075	51.40	n.a.	MB*
3	40.00	n.a.	66.234	53.515	4.56	n.a.	Rd
4	53.91	n.a.	91.311	143.847	12.26	n.a.	BMB*
Total:			897.748	1173.302	100.00	0.000	

Figure S49: Chiral HPLC chromatogram of **TP_A4.9N4CY** (entry 1)

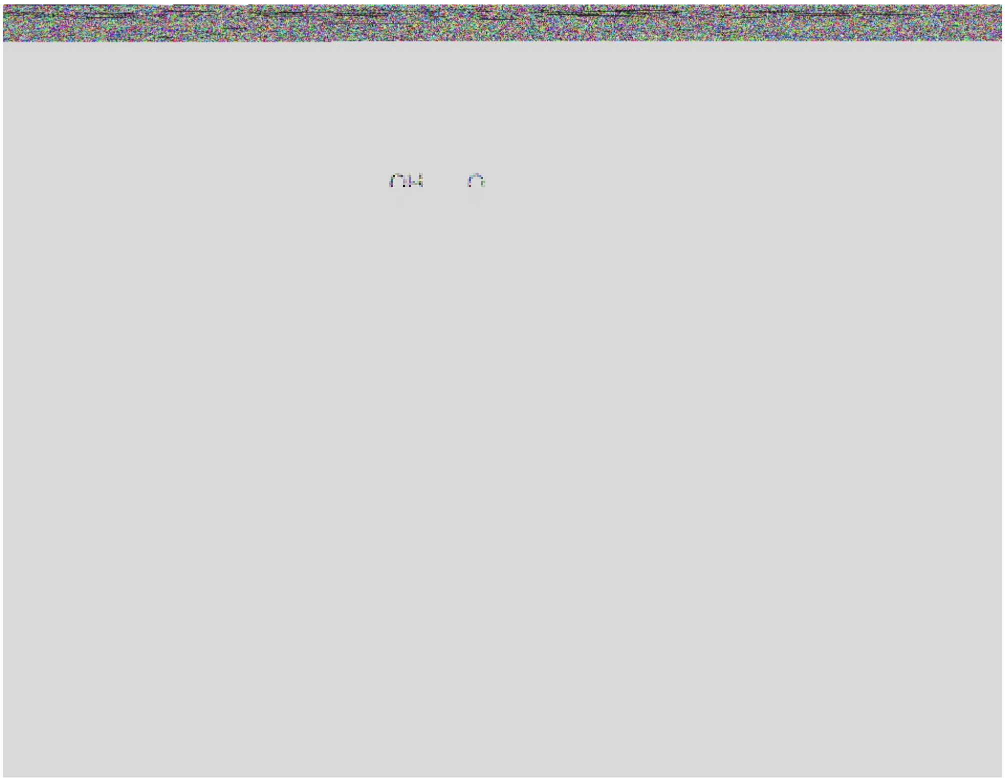

Figure SR: Chiral HPLC chromatogram of **racemate TP_A4N4CY-R15**

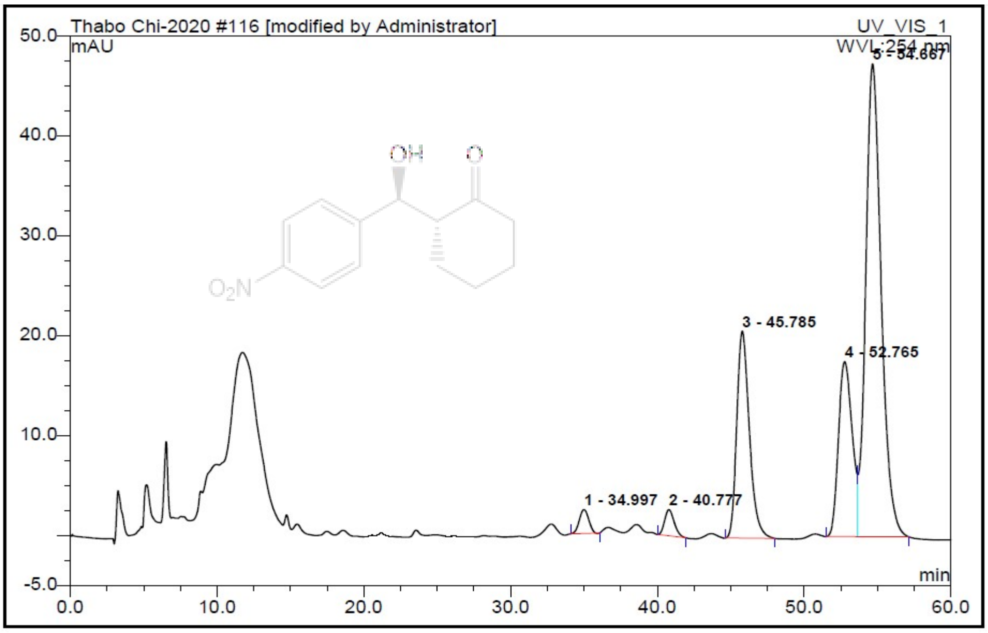

No.	Ret.Time min	Peak Name	Height mAU	Area mAU*min	Rel.Area %	Amount	Type
1	35.00	n.a.	2.391	1.759	1.73	n.a.	BMB*
2	40.78	n.a.	2.615	1.975	1.95	n.a.	BMB*
3	45.79	n.a.	20.731	20.635	20.34	n.a.	BMB*
4	52.77	n.a.	17.529	19.034	18.77	n.a.	BM *
5	54.67	n.a.	47.354	58.025	57.21	n.a.	MB*
Total:			90.620	101.428	100.00	0.000	

Figure S50: Chiral HPLC chromatogram of **TP_A4.13N4CY** (entry 4)

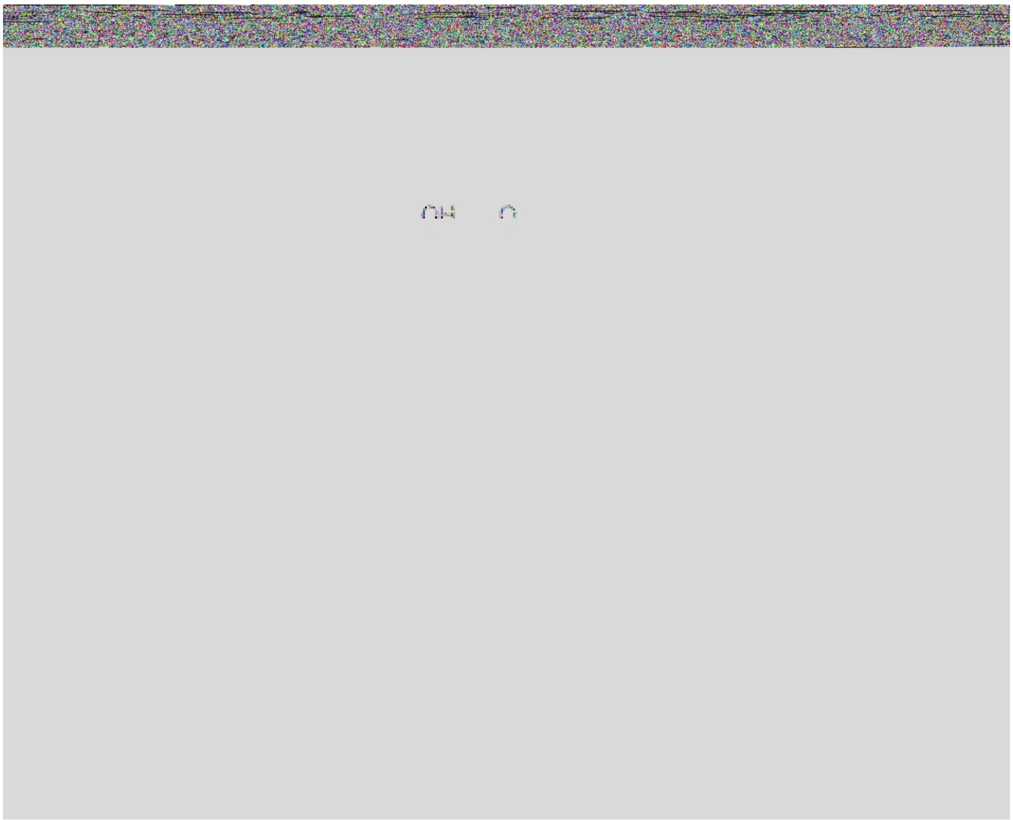

Figure SR: Chiral HPLC chromatogram of **racemate TP_A4N4CY-R14**

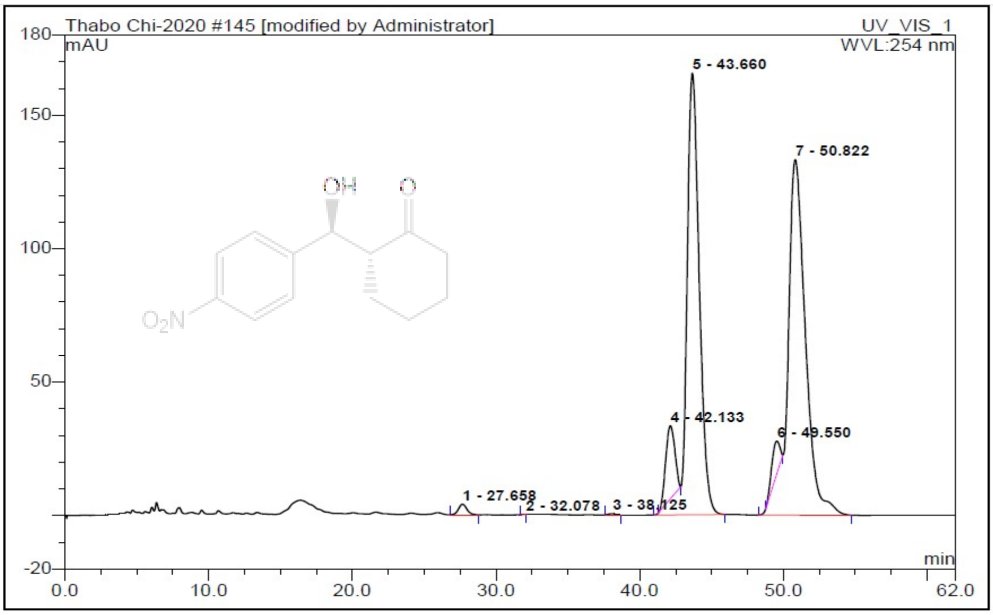

No.	Ret.Time min	Peak Name	Height mAU	Area mAU*min	Rel.Area %	Amount	Type
1	27.66	n.a.	3.954	2.686	0.69	n.a.	BMB*
2	32.08	n.a.	0.008	0.003	0.00	n.a.	BMB*
3	38.13	n.a.	0.484	0.284	0.07	n.a.	BMB*
4	42.13	n.a.	27.400	20.285	5.18	n.a.	Ru
5	43.66	n.a.	165.417	168.513	43.02	n.a.	BMB*
6	49.55	n.a.	12.085	8.313	2.12	n.a.	Ru
7	50.82	n.a.	133.210	191.609	48.92	n.a.	BMB*
Total:			342.558	391.692	100.00	0.000	

Figure S51: Chiral HPLC chromatogram of **TP_A4.14N4CY** (entry 6)

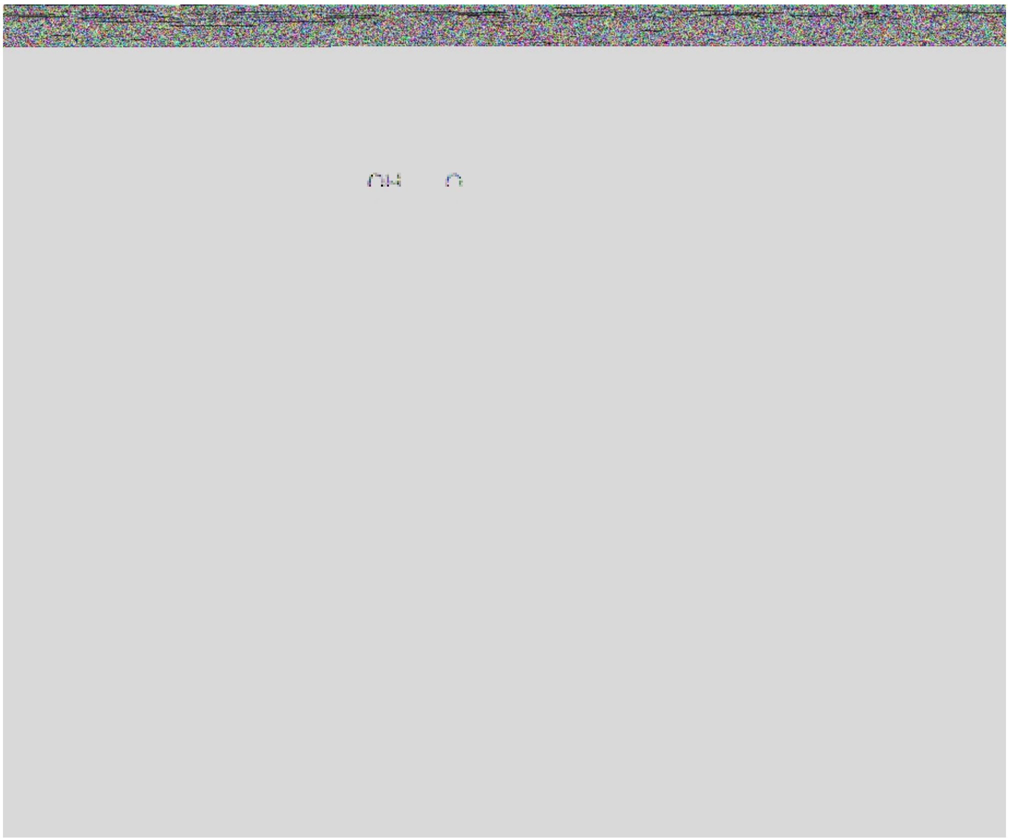

Figure SR: Chiral HPLC chromatogram of **racemate TP_A4N4CY-15**

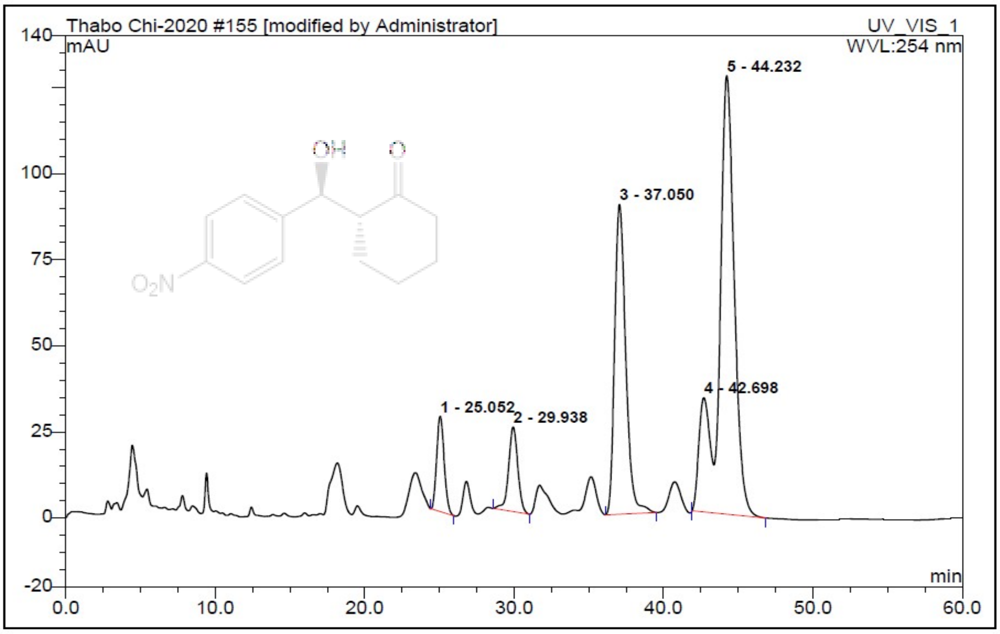

No.	Ret.Time min	Peak Name	Height mAU	Area mAU*min	Rel.Area %	Amount	Type
1	25.05	n.a.	27.625	16.077	5.92	n.a.	BMB*
2	29.94	n.a.	24.568	17.622	6.48	n.a.	BMB*
3	37.05	n.a.	89.999	77.484	28.51	n.a.	BMB*
4	42.70	n.a.	33.210	0.000	0.00	n.a.	BMb*
5	44.23	n.a.	127.454	160.564	59.09	n.a.	bMB*
Total:			302.857	271.747	100.00	0.000	

Figure S52: Chiral HPLC chromatogram of **TP_A4.15N4CY** (entry 7)

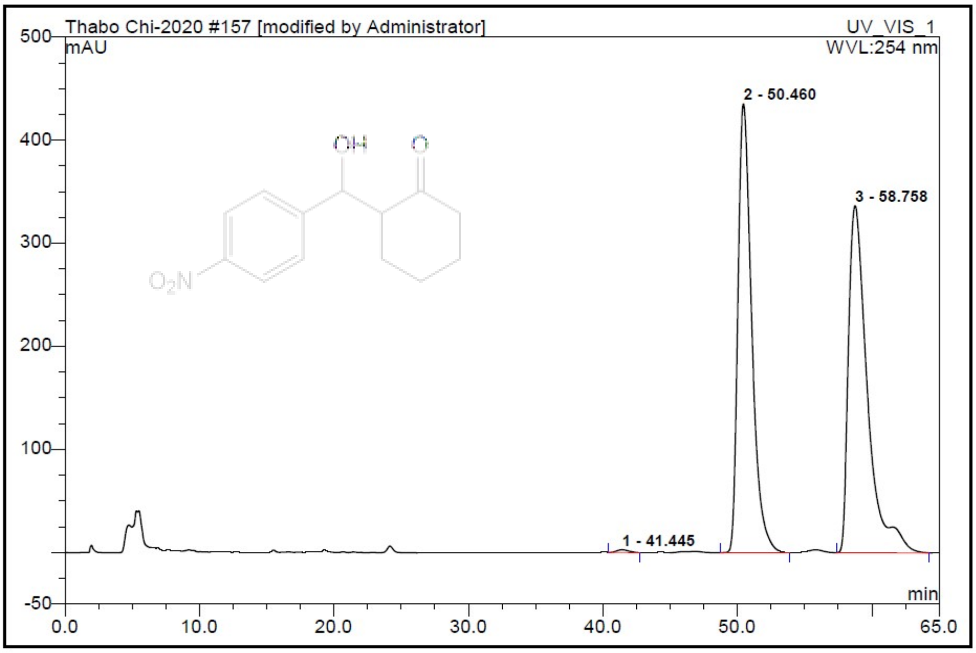

No.	Ret.Time min	Peak Name	Height mAU	Area mAU*min	Rel.Area %	Amount	Type
1	41.45	n.a.	2.604	2.643	0.25	n.a.	BMB*
2	50.46	n.a.	435.305	517.082	48.38	n.a.	BMB
3	58.76	n.a.	336.497	549.047	51.37	n.a.	BMB*
Total:			774.406	1068.772	100.00	0.000	

Figure SR: Chiral HPLC chromatogram of **racemate TP_A4N4CY-R16**

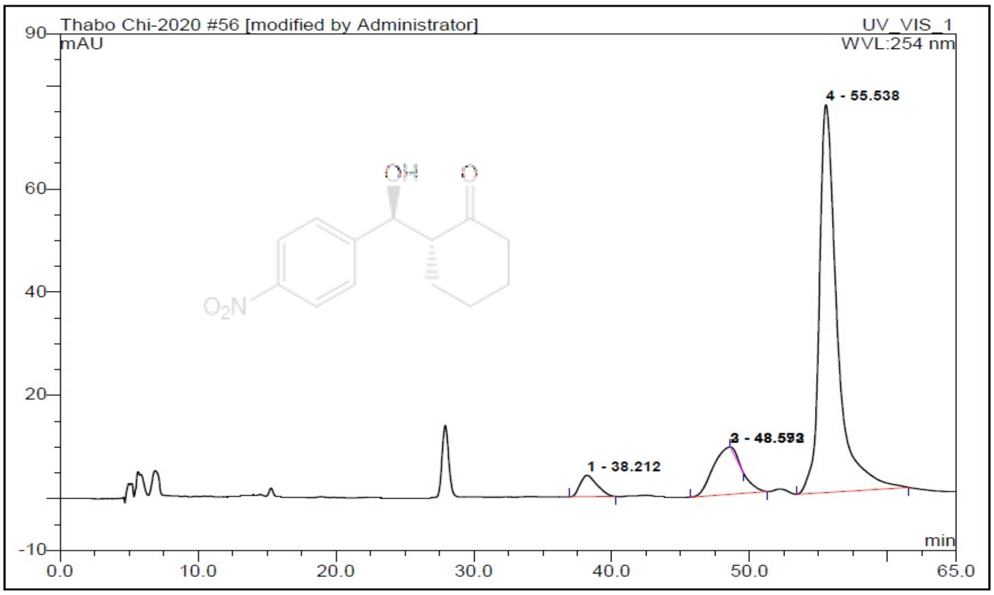

No.	Ret.Time min	Peak Name	Height mAU	Area mAU*min	Rel.Area %	Amount	Type
1	38.21	n.a.	4.138	5.849	4.16	n.a.	BMB*
2	48.57	n.a.	9.164	21.210	15.08	n.a.	BMB*
3	48.59	n.a.	0.036	0.496	0.35	n.a.	Rd
4	55.54	n.a.	75.124	113.102	80.41	n.a.	MB*
Total:			88.461	140.657	100.00	0.000	

Figure S53: Chiral HPLC chromatogram of **TP_A4.16N4CY** (entry 8)

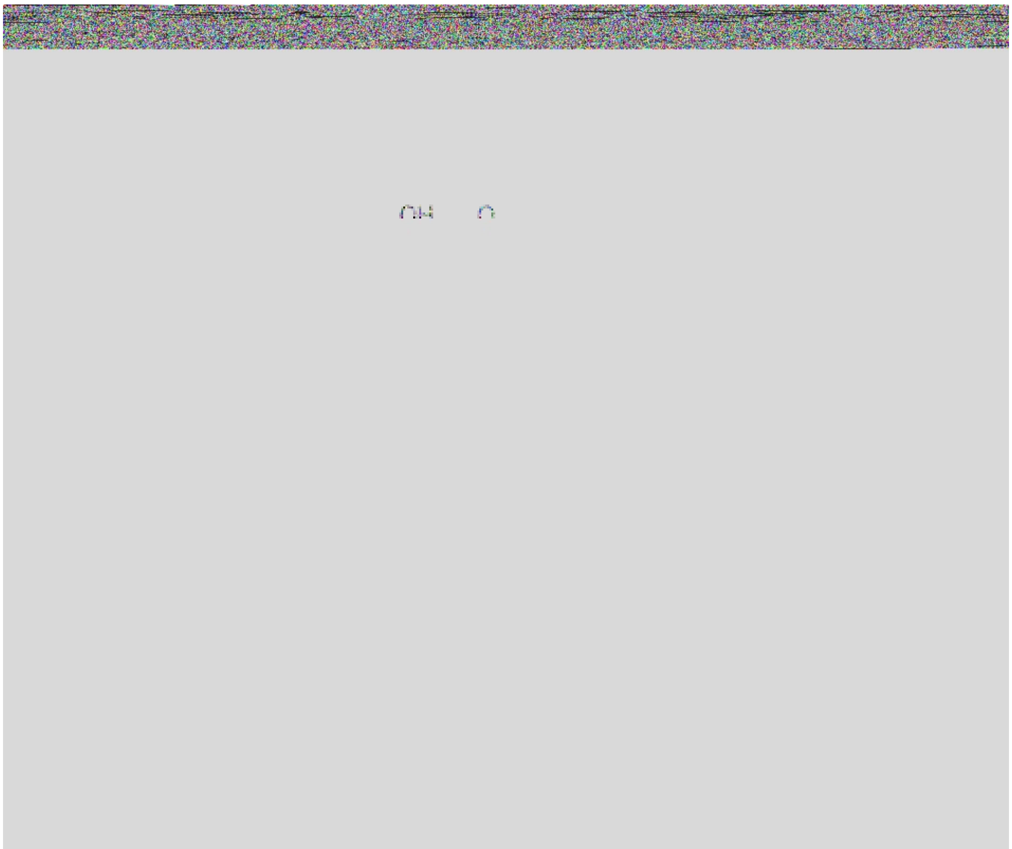

Figure SR: Chiral HPLC chromatogram of **racemate TP_A4N4CY-R17**

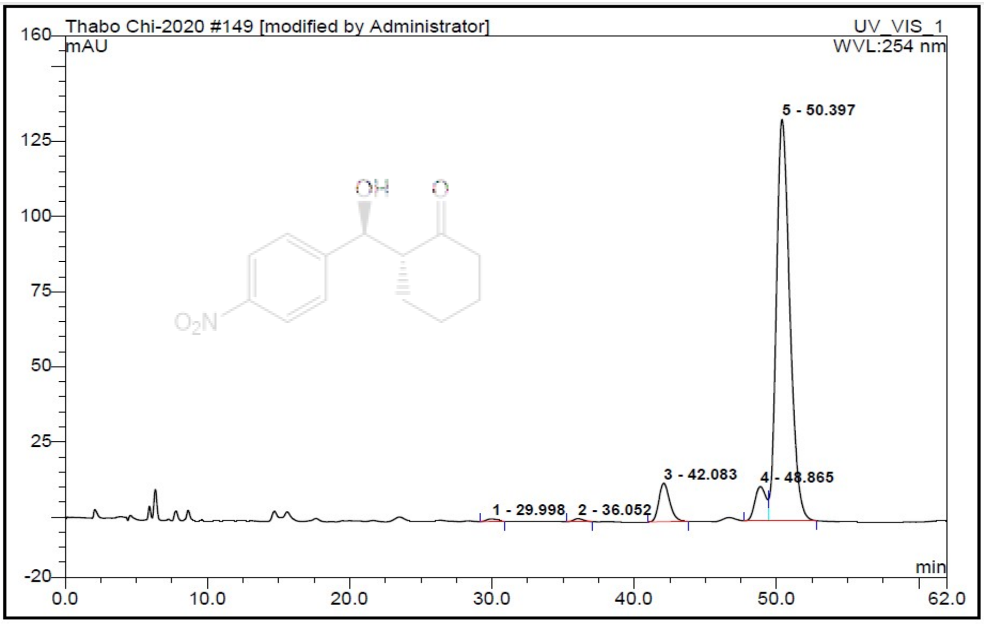

No.	Ret.Time min	Peak Name	Height mAU	Area mAU*min	Rel.Area %	Amount	Type
1	30.00	n.a.	0.908	0.784	0.46	n.a.	BMB*
2	36.05	n.a.	1.048	0.807	0.48	n.a.	BMB*
3	42.08	n.a.	12.788	11.323	6.68	n.a.	BMB*
4	48.87	n.a.	11.390	9.960	5.87	n.a.	BM *
5	50.40	n.a.	133.447	146.715	86.51	n.a.	MB*
Total:			159.581	169.589	100.00	0.000	

Figure S54: Chiral HPLC chromatogram of **TP_A4.17N4CY** (entry 9)

Catalyst Recycling: Reaction between cyclohexanone and *p*-nitrobenzaldehyde (**Table 6**)

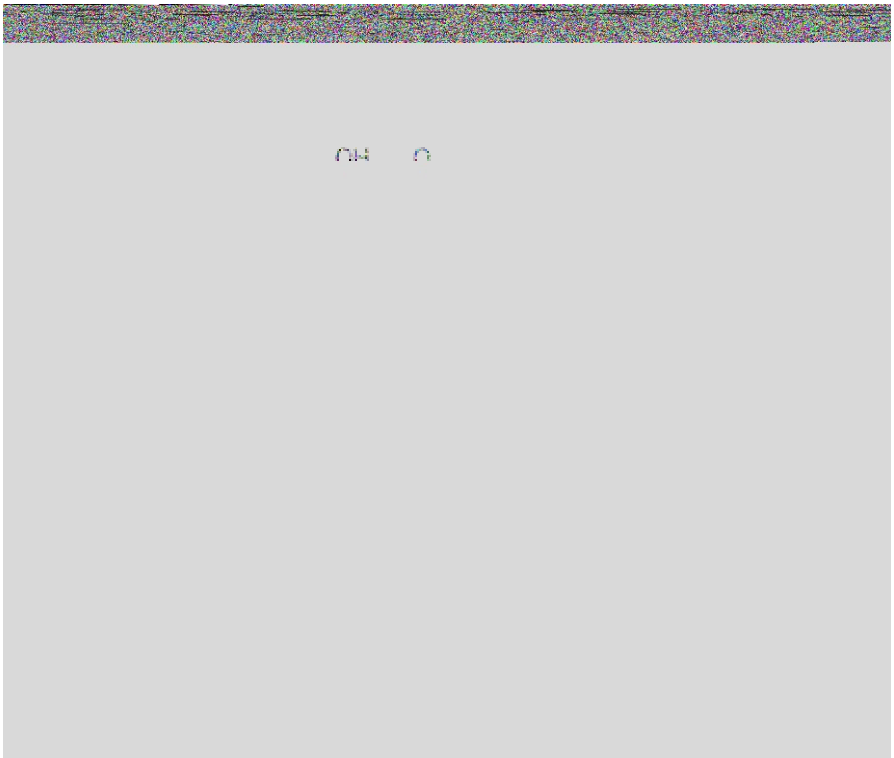

Figure SR: Chiral HPLC chromatogram of **racemate TP_A4N4CY-R18**

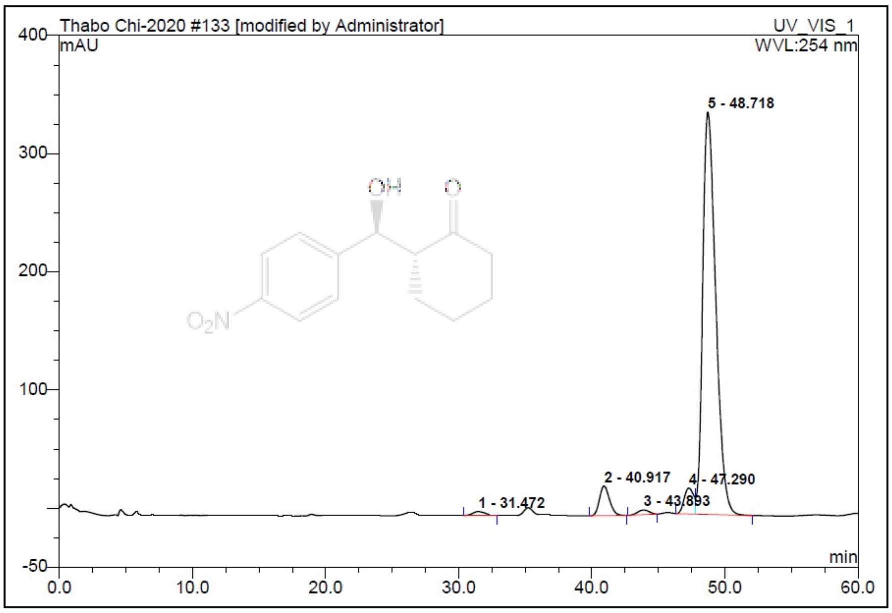

No.	Ret.Time min	Peak Name	Height mAU	Area mAU*min	Rel.Area %	Amount	Type
1	31.47	n.a.	3.412	3.731	0.86	n.a.	BMB*
2	40.92	n.a.	25.122	21.977	5.07	n.a.	BMB*
3	43.89	n.a.	3.977	3.971	0.92	n.a.	BMB*
4	47.29	n.a.	21.839	17.770	4.10	n.a.	BM *
5	48.72	n.a.	340.588	386.386	89.06	n.a.	MB*
Total:			394.937	433.835	100.00	0.000	

Figure S55: Chiral HPLC chromatogram of **TP_A4.18N4CY** (entry 5)

Table A: Analytical data and Chiral-phase HPLC spectra analysis for selected aldol products using Lux 5μm Cellulose-1 chiral column

Structure	Aldol product	Eluent; **%ratio**	Flow rate; **Wavelength (nm)**	Retention time (min)
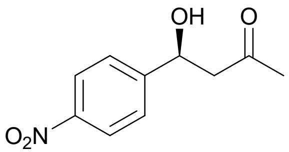	TP_A1.1N4ACE	Hexane/IPA; **85/15**	0.6 mL; **254**	t_{Rmajor} = 23.38; t_{Rminor} = 30.42
	TP_A1.2N4ACE	Hexane/IPA; **85/15**	0.6 mL; **254**	t_{Rmajor} = 24.52; t_{Rminor} = 32.07
	TP_A1.3N4ACE	Hexane/IPA; **85/15**	0.6 mL;**254**	t_{Rminor} = 22.37; t_{Rmajor} = 27.88
	TP_A1.4N4ACE	Hexane/IPA; **85/15**	0.6 mL; **254**	t_{Rminor} = 23.13; t_{Rmajor} = 28.80
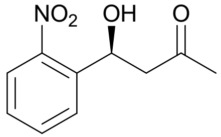	TP_A2.1N2ACE	Hexane/IPA; **90/10**	0.6 mL; **254**	t_{Rmajor} = 22.75; t_{Rmajor} = 26.31
	TP_A2.2N2ACE	Hexane/IPA; **90/10**	0.6 mL; **254**	t_{Rmajor} =19.31; t_{Rminor} = 22.29
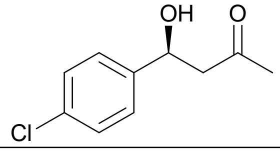	TP_A3Cl4ACE	Hexane/IPA; **85/15**	0.7 mL; **254**	t_{Rminor} = 14.77; t_{Rmajor} = 16.79
	Using cyclohexanone as solvent for reaction between cyclohexanone and aromatic aldehydes **(Table 4-2)**			
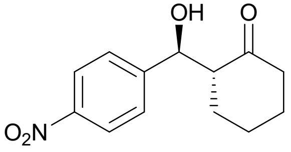	TP_A4.1N4CY	Hexane/IPA; **94/6**	0.6 mL; **254**	t_{Rminor} = 39.09; t_{Rmajor} = 44.17
	TP_A4.2N4CY	Hexane/IPA; **94/6**	0.6 mL; **254**	t_{Rmajor} = 38.63; t_{Rminor}= 43.60
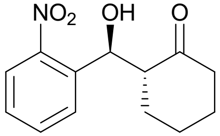	TP_A5.1N4CY	Hexane/IPA; **95/5**	0.5 mL;**254**	t_{Rmajor} = 39.28; t_{Rminor} = 44.15
	TP-A5.2N4CY	Hexane/IPA; **95/5**	0.5 mL; **254**	t_{Rmajor} =38.63; t_{Rminor}=43.60

	Selected Aldol products using **organic solvents (Table 4-3)**			
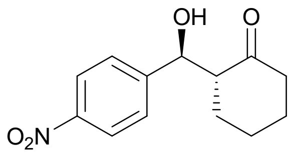	TP_A4.3N4CY	Hexane/IPA; **95/5**	0.7mL, **254**	t_{Rmajor} = 37.91; t_{Rminor} = 42.57
	TP_A4.4N4CY	Hexane/IPA; **95/5**	0.6mL, **254**	t_{Rmajor} = 39.19 t_{Rminor} = 45.06
	TP_A4.5N4CY	Hexane/IPA; **94/6**	0.7mL; **254**	t_{Rminor} = 39.08; t_{Rmajor} = 45.61
	TP_A4.6N4CY	Hexane/IPA; **95/5**	0.6mL; **254**	t_{Rminor} = 43.19; t_{Rmajor} = 48.48
	TP_A4.7N4CY	Hexane/IPA; **96/4**	0.8mL; **254**	t_{Rmajor} = 29.42; t_{Rminor} = 26.19
	Using **8 mol%** catalyst loading **(Table 4-5)**			
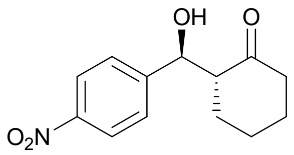	TP_A4.10N4CY	Hexane/IPA; **94/6**	0.6mL; **254**	t_{Rmajor} = 44.50; t_{Rminor} = 51.20
	TP_A4.9N4CY	Hexane/IPA; **94/6**	0.7mL; **254**	t_{Rminor} = 34.49; t_{Rmajor} = 38.65
	TP_A4.13N4CY	Hexane/IPA; **95/5**	0.6mL; **254**	t_{Rminor} = 45.79; t_{Rmajor} = 54.67
	TP_A4.14N4CY	Hexane/IPA; **95/5**	0.6mL; **254**	t_{Rminor} = 43.66; t_{Rmajor} = 50.82
	TP_A4.15N4CY	Hexane/IPA; **94/6**	0.6mL; **254**	t_{Rmajor} = 37.05; t_{Rmajor} = 44.23
	TP_A4.16N4CY	Hexane/IPA; **95/5**	0.7mL; **254**	t_{Rminor} = 48.57; t_{Rmajor} = 55.54
	TP_A4.17N4CY	Hexane/IPA; **95/5**	0.8mL; **254**	t_{Rminor} =42.08; t_{Rmajor} = 50.40
	TP_A4.18N4CY	Hexane/IPA; **95/5**	0.8mL; **254**	t_{Rminor} = 40.92; t_{Rmajor} = 48.72

Selected NMR spectra for determination of the syn/anti (dr) for aldol products by ^{1}H NMR

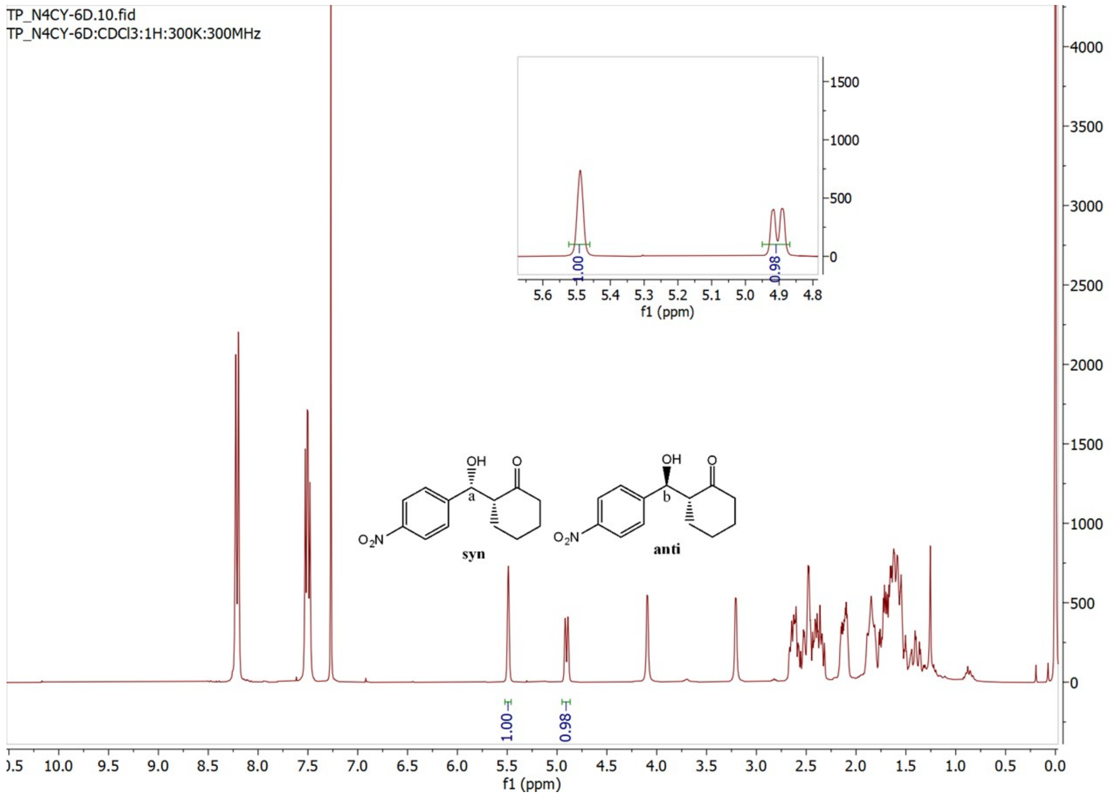

Figure S56: ^{1}H NMR spectrum of the crude product of **TP_A4.2N4CY**

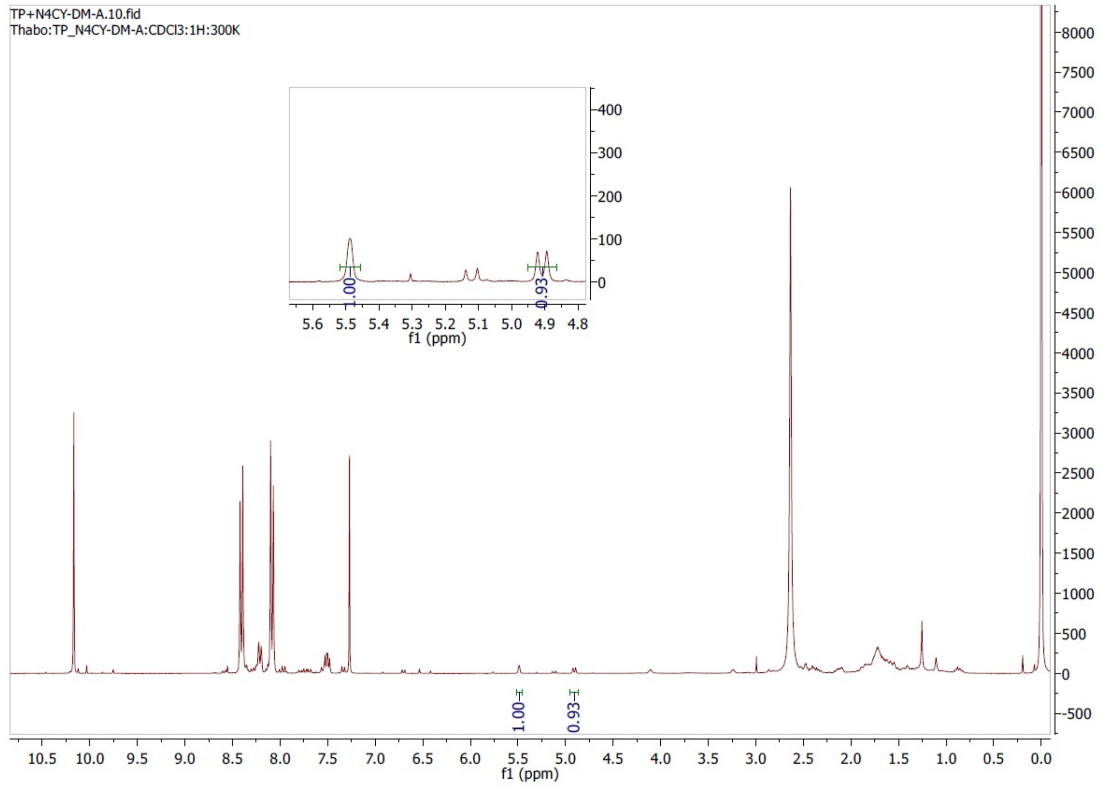

Figure S57: ^{1}H NMR spectrum of the crude product of **TP_A4.3N4CY**

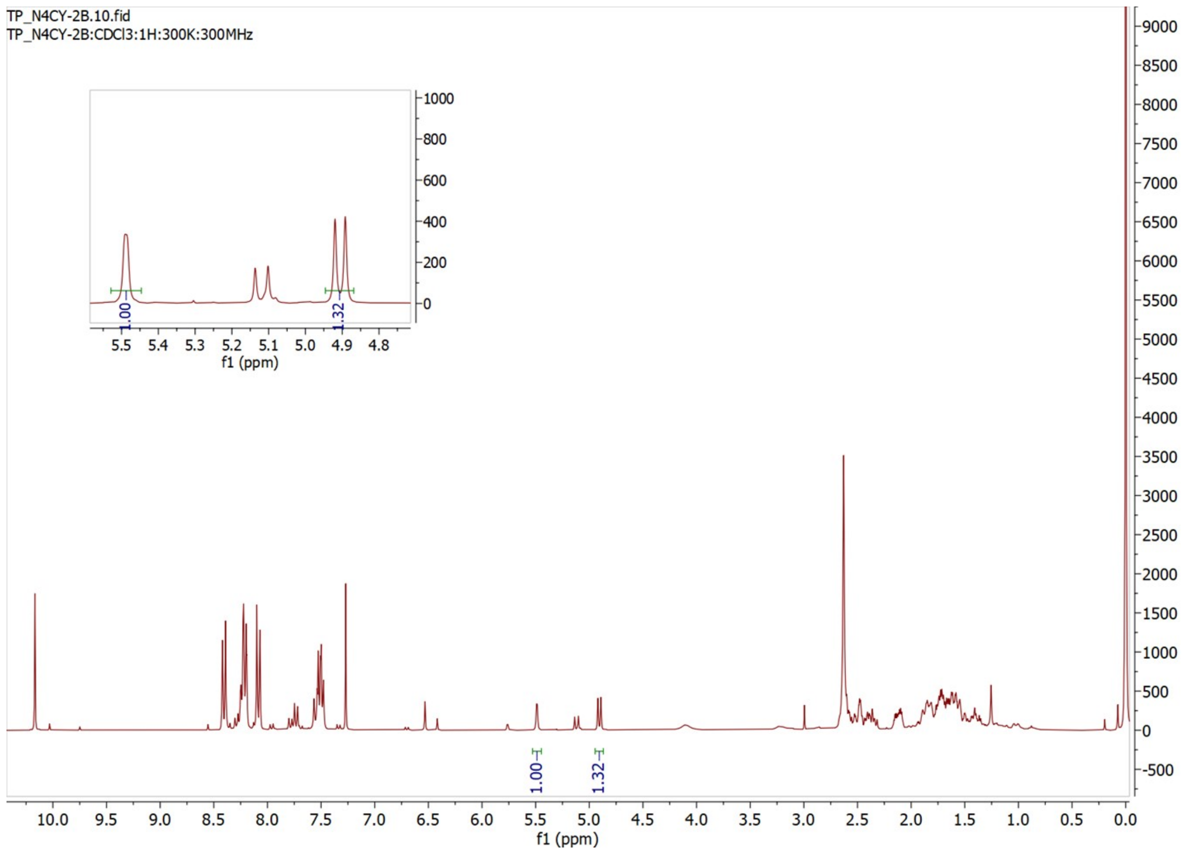

Figure S58: ^{1}H NMR spectrum of the crude product of **TP_A4.4N4CY**

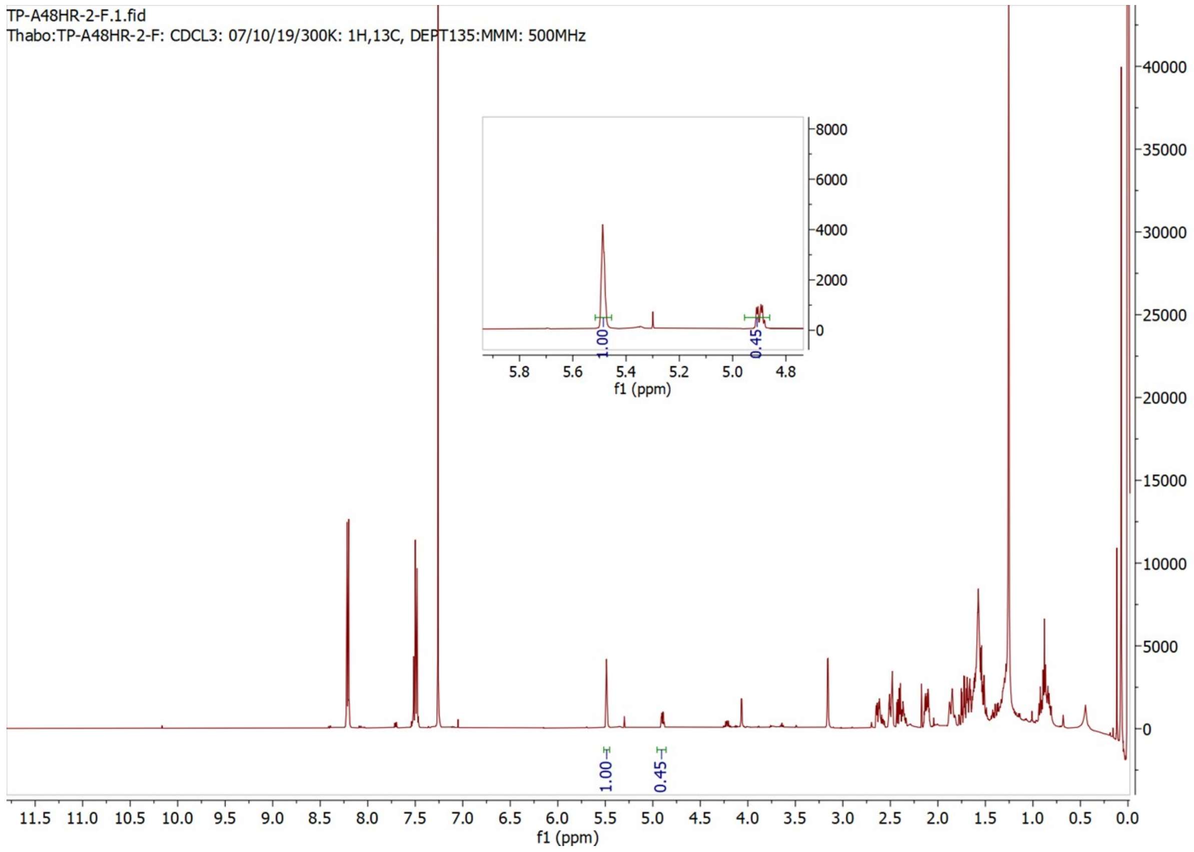

Figure S59: ^{1}H NMR spectrum of the crude product **TP_A4.5N4CY**

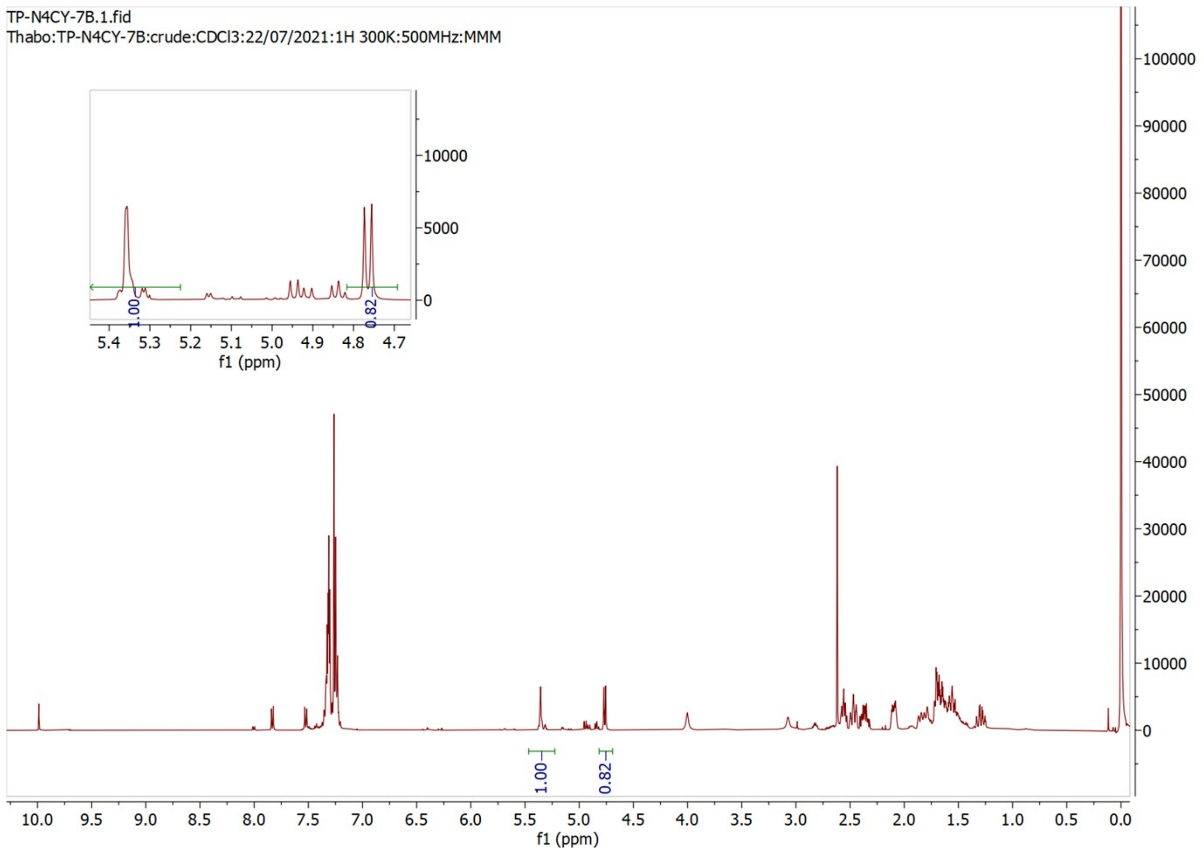

Figure S60: ^{1}H NMR spectrum of the crude product of **TP_A4.6N4CY**

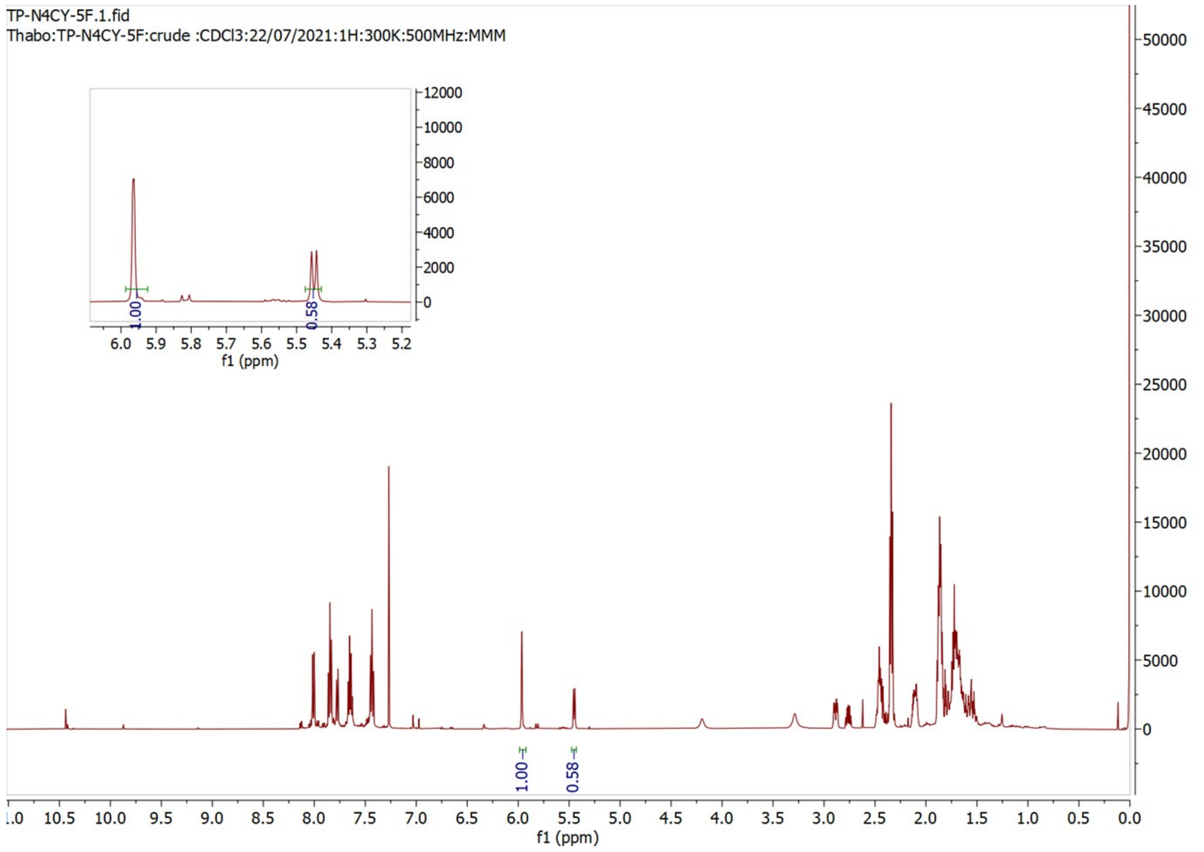

Figure S61: ^{1}H NMR spectrum of the crude product of **TP_A4.8N4CY**

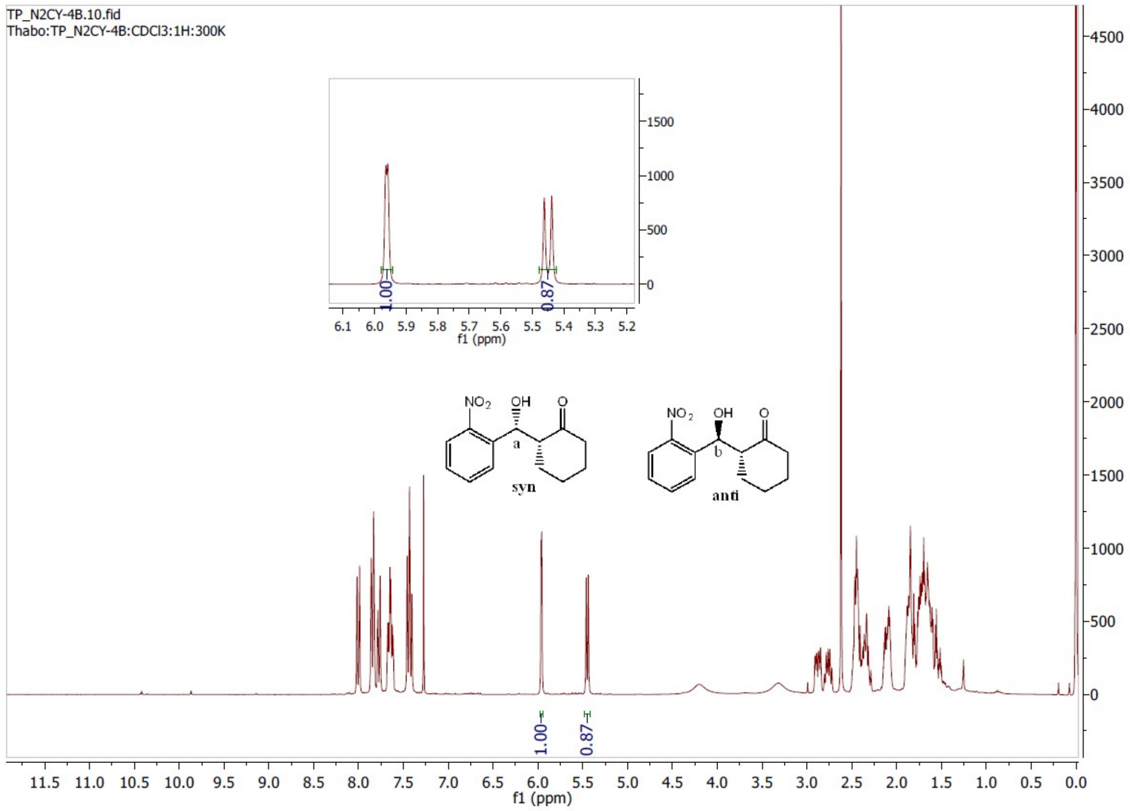

Figure S62: ^{1}H NMR spectrum of the crude product of **TP_A5.1N2CY**

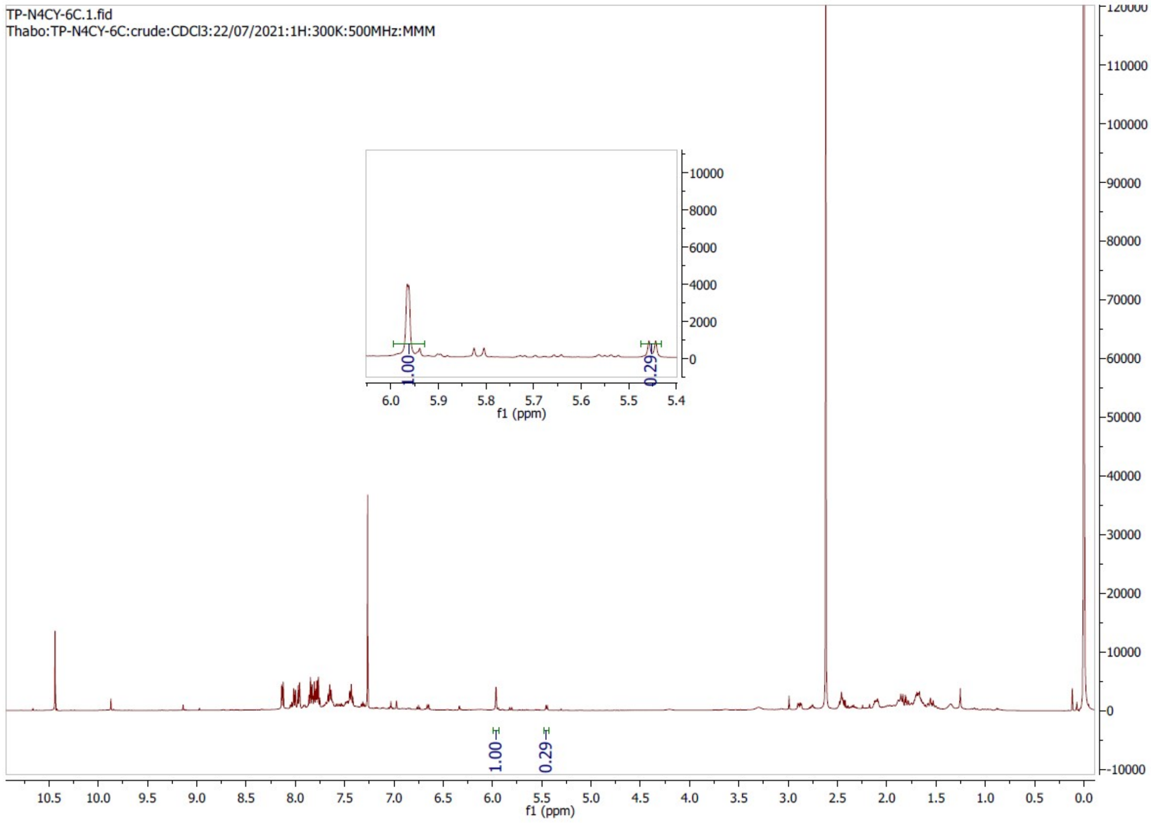

Figure S63:^{1}H NMR spectrum of the crude product of **TP_A5.2N2CY**

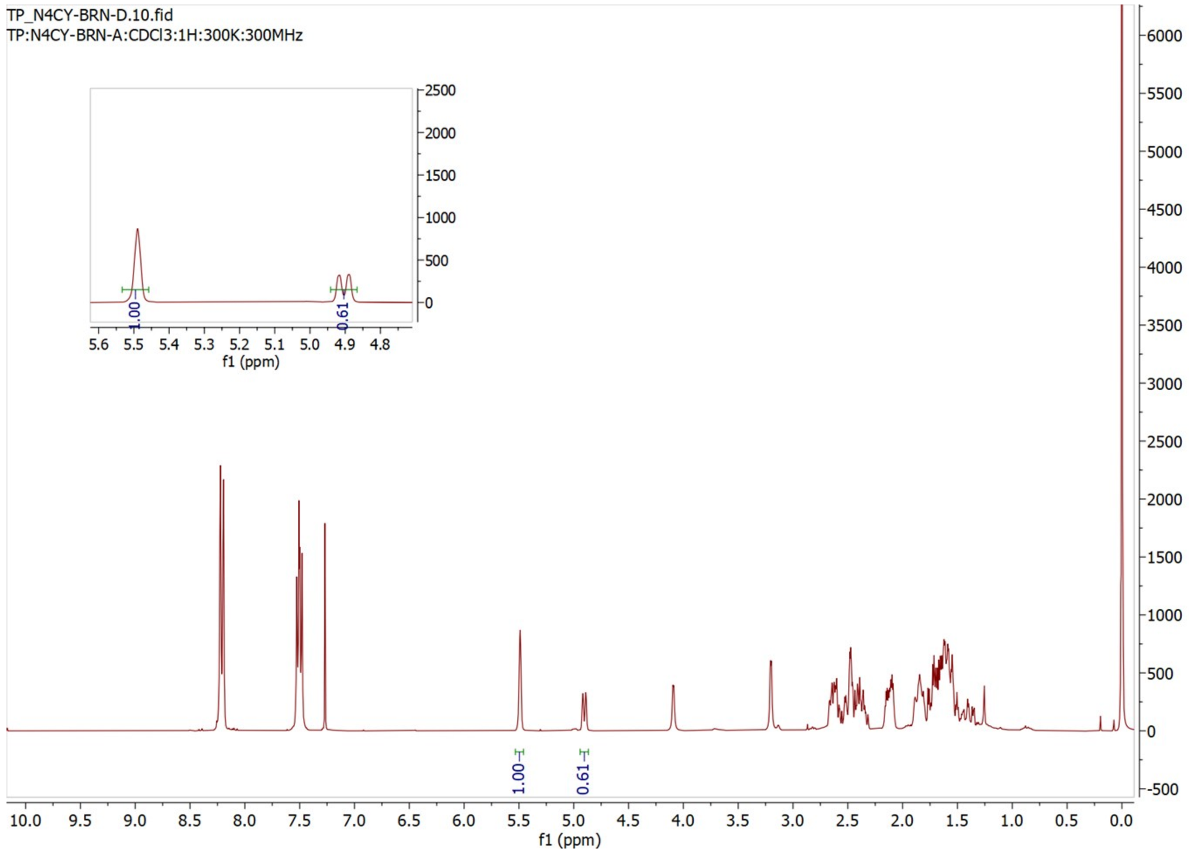

Figure S64: ^{1}H NMR spectrum of the crude product **TP_A4.9N4CY**

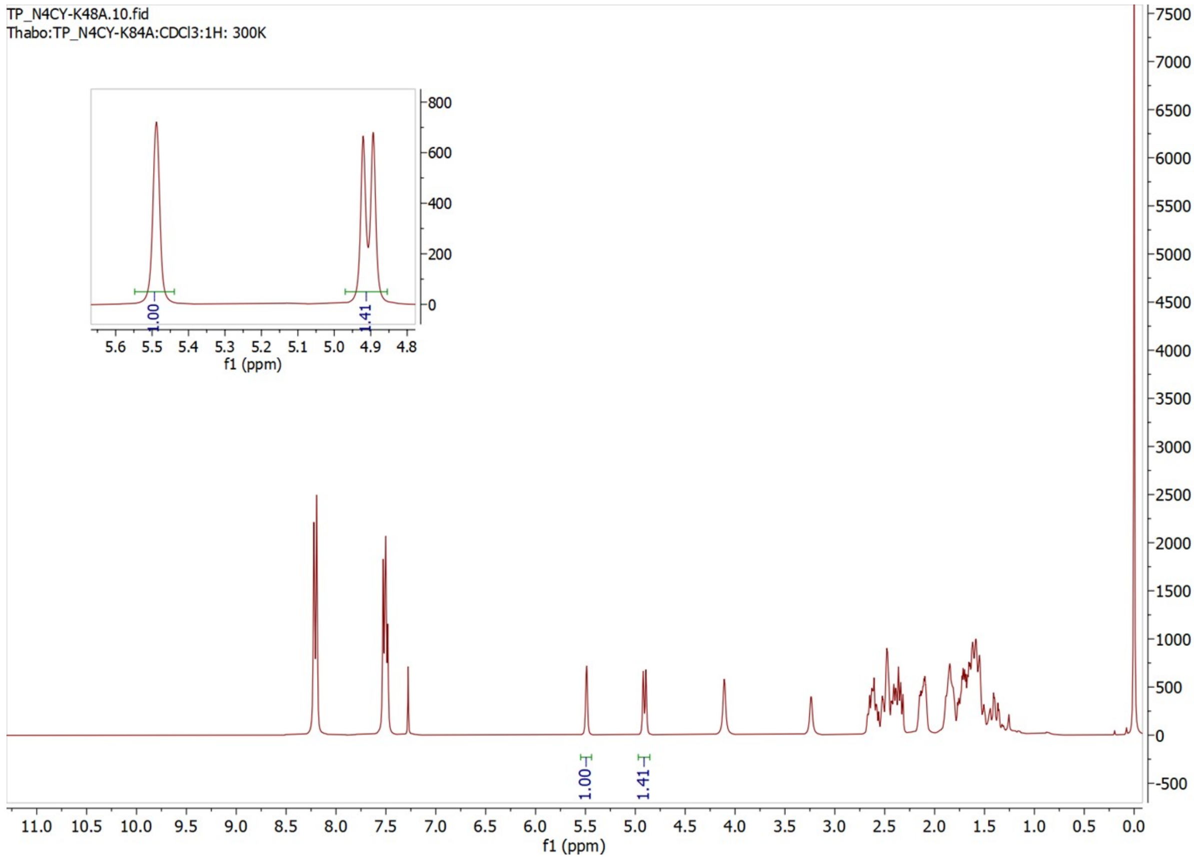

Figure S65: ^{1}H NMR spectrum of the crude product **TP_A4.10N4CY**

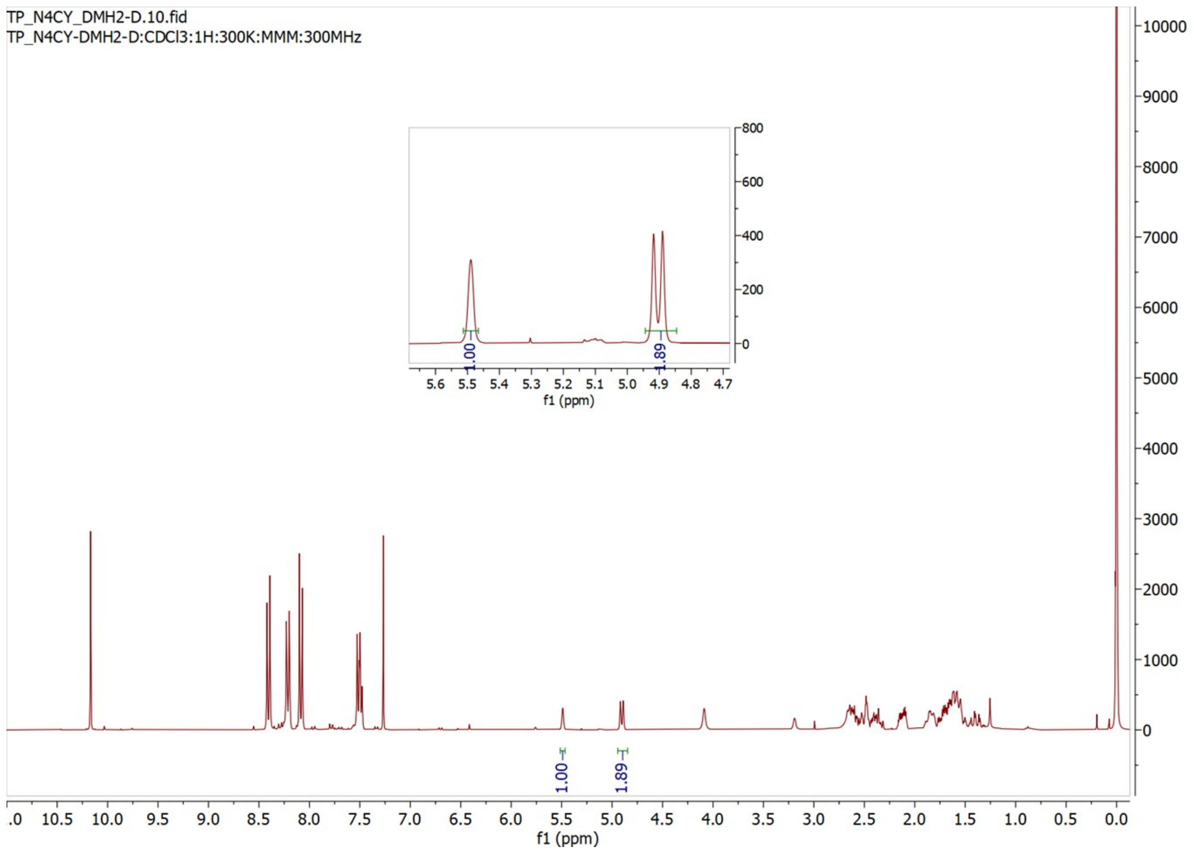

Figure S66: ^{1}H NMR spectrum of the crude product **TP_A4.12N4CY**

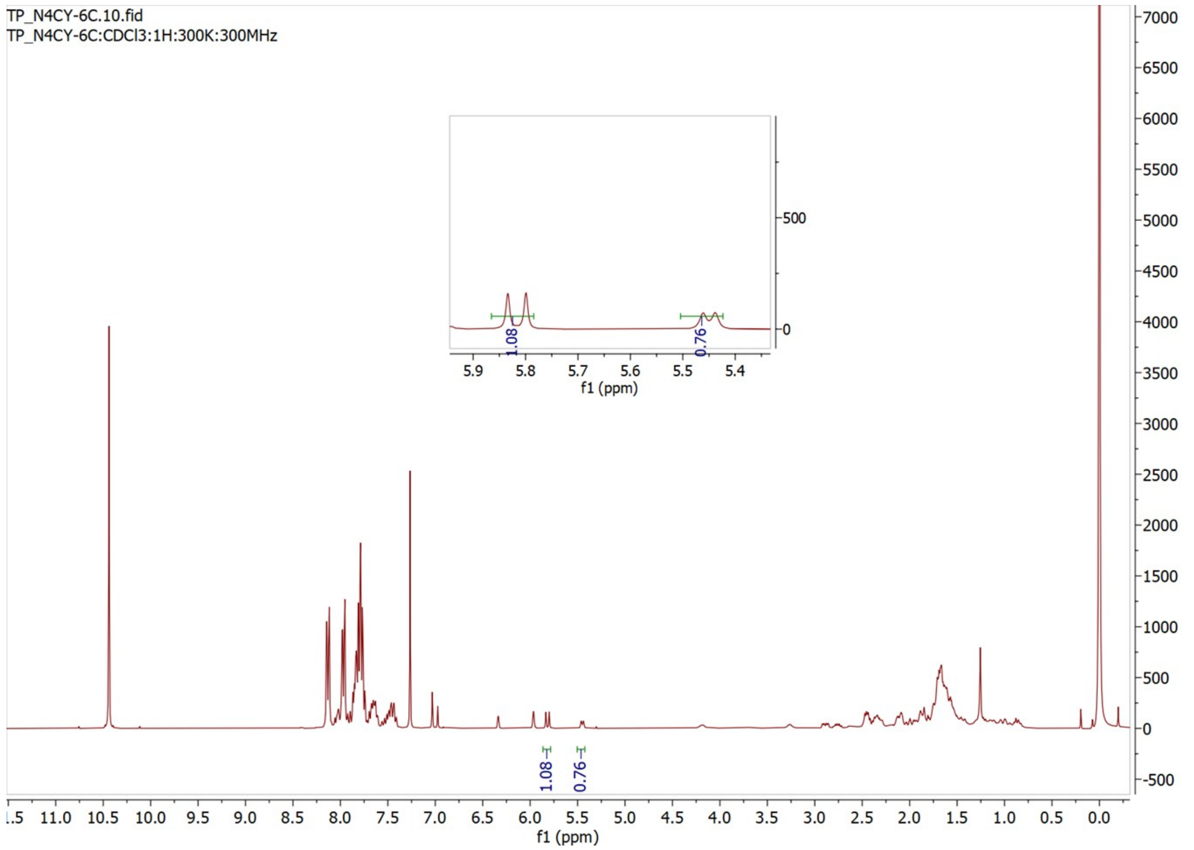

Figure S67: ^{1}H NMR spectrum of the crude product of **TP_A4.14N4CY**

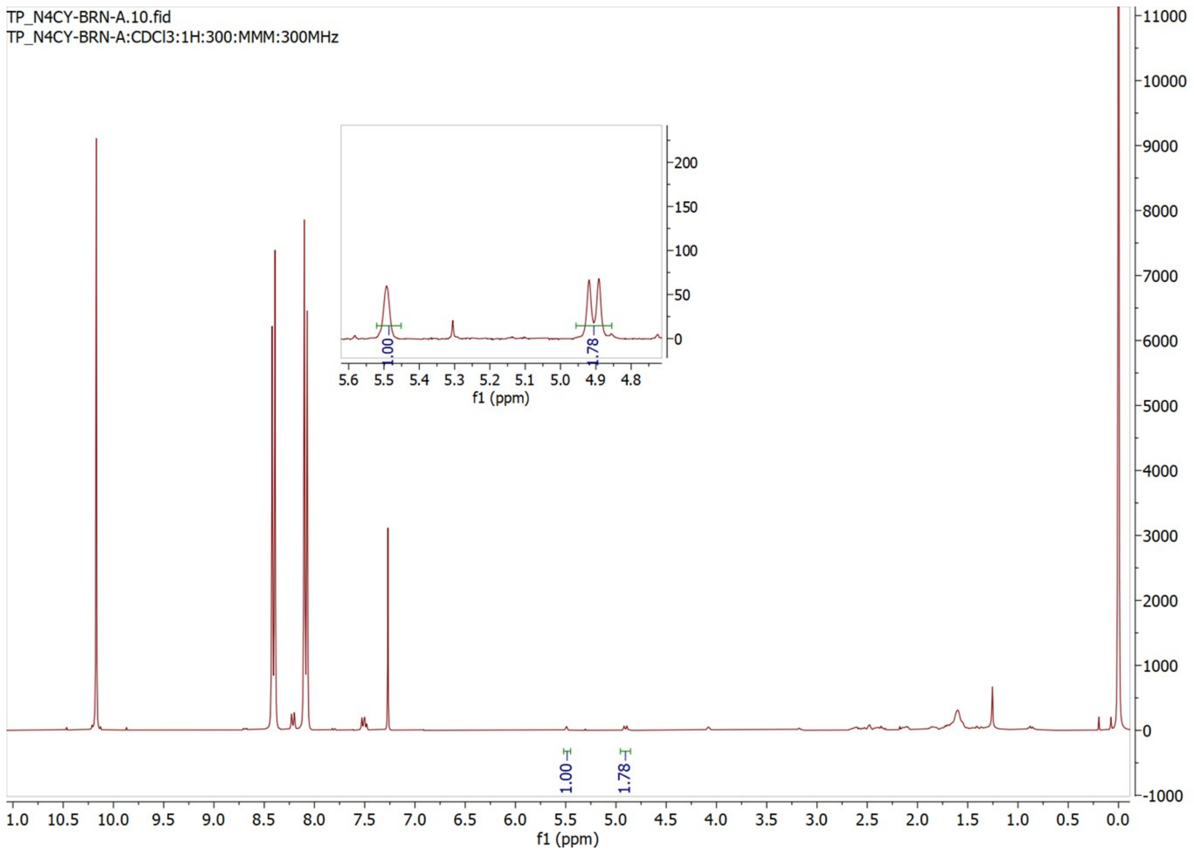

Figure S68: ^{1}H NMR spectrum of the crude product of **TP_A4.16N4CY**

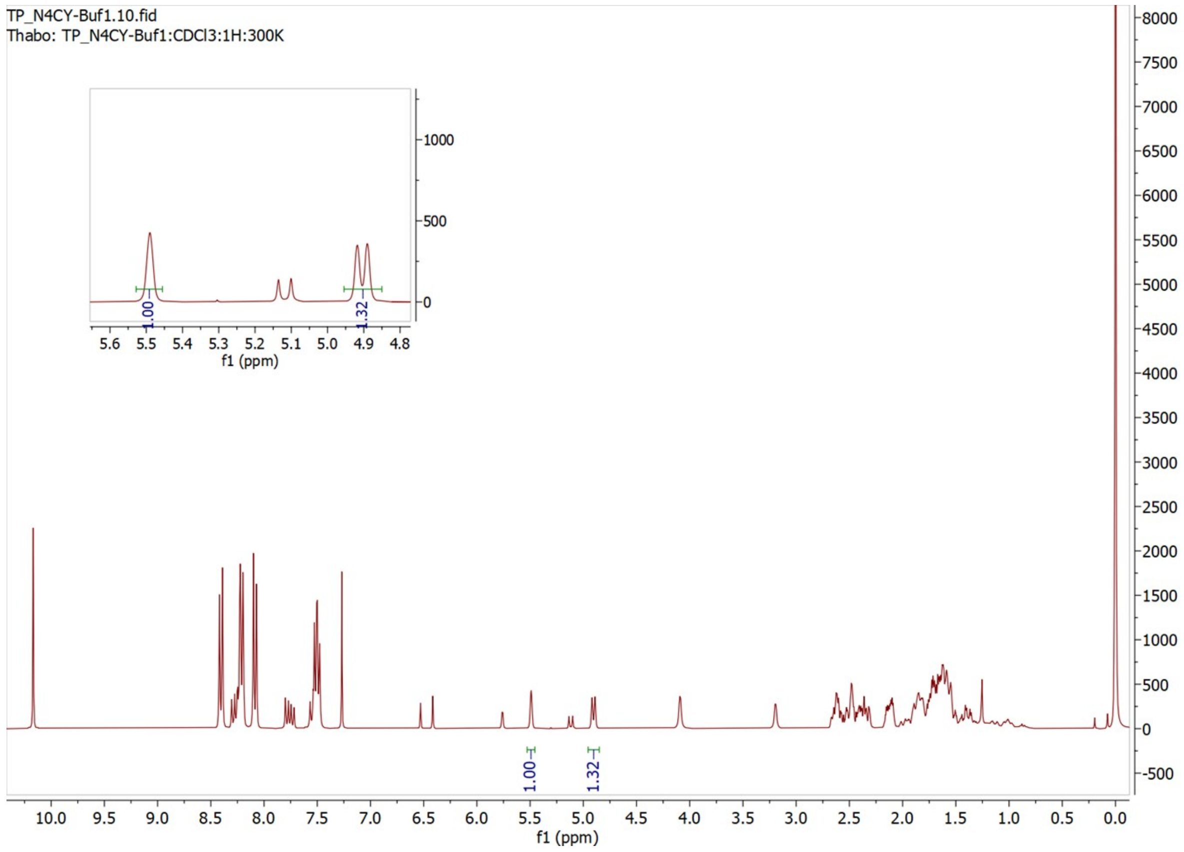

Figure S69: ^{1}H NMR spectrum of the crude product **TP_A4.17N4CY**
